# Resolving Indigenous village occupations and social history across the long century of European permanent settlement in Northeastern North America: The Mohawk River Valley ~1450-1635 CE

**DOI:** 10.1371/journal.pone.0258555

**Published:** 2021-10-15

**Authors:** Sturt W. Manning, Brita Lorentzen, John P. Hart

**Affiliations:** 1 Cornell Tree-Ring Laboratory, Department of Classics, and Cornell Institute of Archaeology and Material Studies, Cornell University, Ithaca, NY, United States of America; 2 The Cyprus Institute, Nicosia, Cyprus; 3 Research and Collections Division, New York State Museum, Albany, NY, United States of America; University at Buffalo - The State University of New York, UNITED STATES

## Abstract

The timeframe of Indigenous settlements in Northeast North America in the 15^th^-17^th^ centuries CE has until very recently been largely described in terms of European material culture and history. An independent chronology was usually absent. Radiocarbon dating has recently begun to change this conventional model radically. The challenge, if an alternative, independent timeframe and history is to be created, is to articulate a high-resolution chronology appropriate and comparable with the lived histories of the Indigenous village settlements of the period. Improving substantially on previous initial work, we report here high-resolution defined chronologies for the three most extensively excavated and iconic ancestral Kanienʼkehá꞉ka (Mohawk) village sites in New York (Smith-Pagerie, Klock and Garoga), and a fourth early historic Indigenous site, Brigg’s Run, and re-assess the wider chronology of the Mohawk River Valley in the mid-15^th^ to earlier 17^th^ centuries. This new chronology confirms initial suggestions from radiocarbon that a wholesale reappraisal of past assumptions is necessary, since our dates conflict completely with past dates and the previously presumed temporal order of these three iconic sites. In turn, a wider reassessment of northeastern North American early history and re-interpretation of Atlantic connectivities in the later 15^th^ through early 17^th^ centuries is required. Our new closely defined date ranges are achieved employing detailed archival analysis of excavation records to establish the contextual history for radiocarbon-dated samples from each site, tree-ring defined short time series from wood charcoal samples fitted against the radiocarbon calibration curve (‘wiggle-matching’), and Bayesian chronological modelling for each of the individual sites integrating all available prior knowledge and radiocarbon dating probabilities. We define (our preferred model) most likely (68.3% highest posterior density) village occupation ranges for Smith-Pagerie of ~1478–1498, Klock of ~1499–1521, Garoga of ~1550–1582, and Brigg’s Run of ~1619–1632.

## Introduction

The 15^th^-17^th^ century CE history of North America is primarily, and until recently almost exclusively, explained, dated, and viewed in terms of European exploration, interests, and subsequent invasion and settlement (all dates in this paper CE). This early history is predominantly informed and told from the evidence of European documentary history [1–5]. In turn, the presence or absence of types of European material culture have formed the basis of archaeological chronology (e.g. [6–8]). Accounts typically start with, and are based around, the brief initial Norse settlement in Newfoundland at L’Anse aux Meadows around 1000, subsequent exploration and likely coastal interactions up to Cabot in 1497, leading to the landing of Cartier (French) in the St. Lawrence in 1534, the settlement at St. Augustine, Florida (Spanish) from 1565, the failed (British) colony of Roanoke in North Carolina in 1585 and 1587, and then the first settlements of Arcadia (French) in modern Nova Scotia from 1604, Jamestown (British) in modern Virginia from 1607 and Fort Nassau (Dutch) in New York from 1614 [1, 5, 9–14]. This complex but very one-sided history has been described in terms of a European timeline and ingrained perspective [15]. The world of North America before, and the world the Europeans invaded, is primarily known from archaeology [16]. There is an established but inherently ethnocentric narrative and timeframe.

In reverse, knowledge of the Indigenous viewpoint is unfortunately limited and primarily indirect—read often ‘against the grain’ from early European sources [6, 17–21]. Efforts to include Indigenous ethnohistoric sources (e.g. [15, 22]) cannot easily reach back directly and specifically to the initial 16^th^ to early 17^th^ century era and beyond. The lack of, and necessity for, an Indigenous oral history perspective for American archaeology and history has been regularly noted (e.g. [23]) and has been argued to provide important information for key socio-political transformations, for example: the creation of the Haudenosaunee League (later Confederacy: of Six Nations including the Mohawk) [24–29]. Attempts to identify specific pre-17^th^ century dates (e.g. [30]) are usually dismissed (e.g. [27] at p.287 n.33). The challenge and complexity, generally, remains around how to introduce such oral history resources into archaeology and history, and whether it can have well-defined, rigorous, chronological value (in the absolute, calendar, versus cultural, sense) (e.g. [31, 32]). The Haudenosaunee comprise a prime example, where attention to oral history can importantly revise and inform the existing narrative derived and constrained by the well-known but very ethnocentric 1851 book by Lewis Henry Morgan ([33], see [34]).

One critical step towards a history that is less affected by ethnocentric biases and assumptions is an accurate independent (neutral) timeframe. Here there is work to be done (despite the best efforts of previous researchers using the materials and approaches that were available to them). The archaeology of northeastern North America during the last several centuries before European entradas has been the subject of intense research and is known in detail in terms of numerous sites of village occupations in various investigated areas [e.g., 6, 35–44]. The spatial dimension of site occupation is thus moderately well-defined. A clear timeline of settlement, however, has long been a problem, and especially in the period from the later 15^th^ through early 17^th^ centuries. Sites and cultural assemblages were typically dated according to the absence or presence of certain types of European trade goods. Absence meant an early site, rare and fragmentary indications denoted sites somewhere from around the time of Cartier onwards (although other earlier interactions with European fishermen or travelers clearly also occurred). More evidence of European items, and especially the appearance of various types of glass beads and specific metal forms (e.g. kettles, knives) placed sites into the late 16^th^ and early 17^th^ centuries. Among sites, comparison of changes in some artefact types were used to suggest apparent social and temporal relationships via seriation analysis (e.g. [8, 9, 45–48]) and more recently via social network analysis [49, 50]. All these methods could yield dates or claimed temporal order or coefficients of similarity, but in terms of timescale they were inherently not independently derived; at best approximate; and, worse, necessarily assumed that all Indigenous societies accepted and used material culture traits and European trade goods and connections as soon as they could and in similar ways—overlooking the rich complications involved in object production, trade, exchange and consumption observed the world over that likely negate simple or transparent step-wise assumptions from object production, through social practice, to final deposition [51–56]. Even our ethnographic assessments and understandings of what constituted value and interest in material culture by Indigenous peoples during the period of early European contacts in America are derived solely from European observers [57] and are thus at best partial and filtered. The nature of human social groupings, their differences, individual and wider human agency, values and beliefs, were all compressed and flat-lined into what was very much a two-dimensional assessment. Independent absolute dating via radiocarbon (^14^C) dating was long deemed not useful because a plateau/reversal in the ^14^C calibration curve created temporal ambiguity ~1480–1620, producing widely spread, or multiple, possible date range(s), and thus a lack of resolution. Dendrochronology was rarely practical on Indigenous sites in northeastern North America as recovered wood remains (primarily charcoal) typically had no more than a few decades of total tree-ring growth preserved.

Recently this entire set of circumstances has changed. The application of accelerator mass spectrometry (AMS) ^14^C dating, which enables analysis of small samples and thus production of larger and relevant datasets (e.g. dating especially short-lived plant materials or specific tree-rings of direct relevance to specific contexts); improved ^14^C calibration curve resolution; and, in particular, the use of Bayesian chronological methods (e.g., [58–61]) enabling the incorporation of archaeological and other information about sample and site temporal relationships, have collectively transformed the ability to resolve dates in archaeology including for later 15^th^ through earlier 17^th^ century Northeast North America [62–65]. In particular, two methods, ideally combined, have been transformational, enabling a revolution in temporal resolution for archaeological sites in the region: (i) ^14^C dating fixed time series of specific tree-rings, even from wood samples preserving short tree-ring sequences (<50 rings), and placing these into calendar time via correlation against the ^14^C calibration curve (so-called ^14^C ‘wiggle-matching’ [66, 67]); and (ii) integration of prior archaeological or ethnohistoric information about stratigraphic and temporal relationships within a site’s history and the temporal constraints on the likely overall maximum duration of such village sites, to greatly constrain and refine ^14^C dating using Bayesian methods (e.g. [62, 68, 69]).

**Fig 1:**
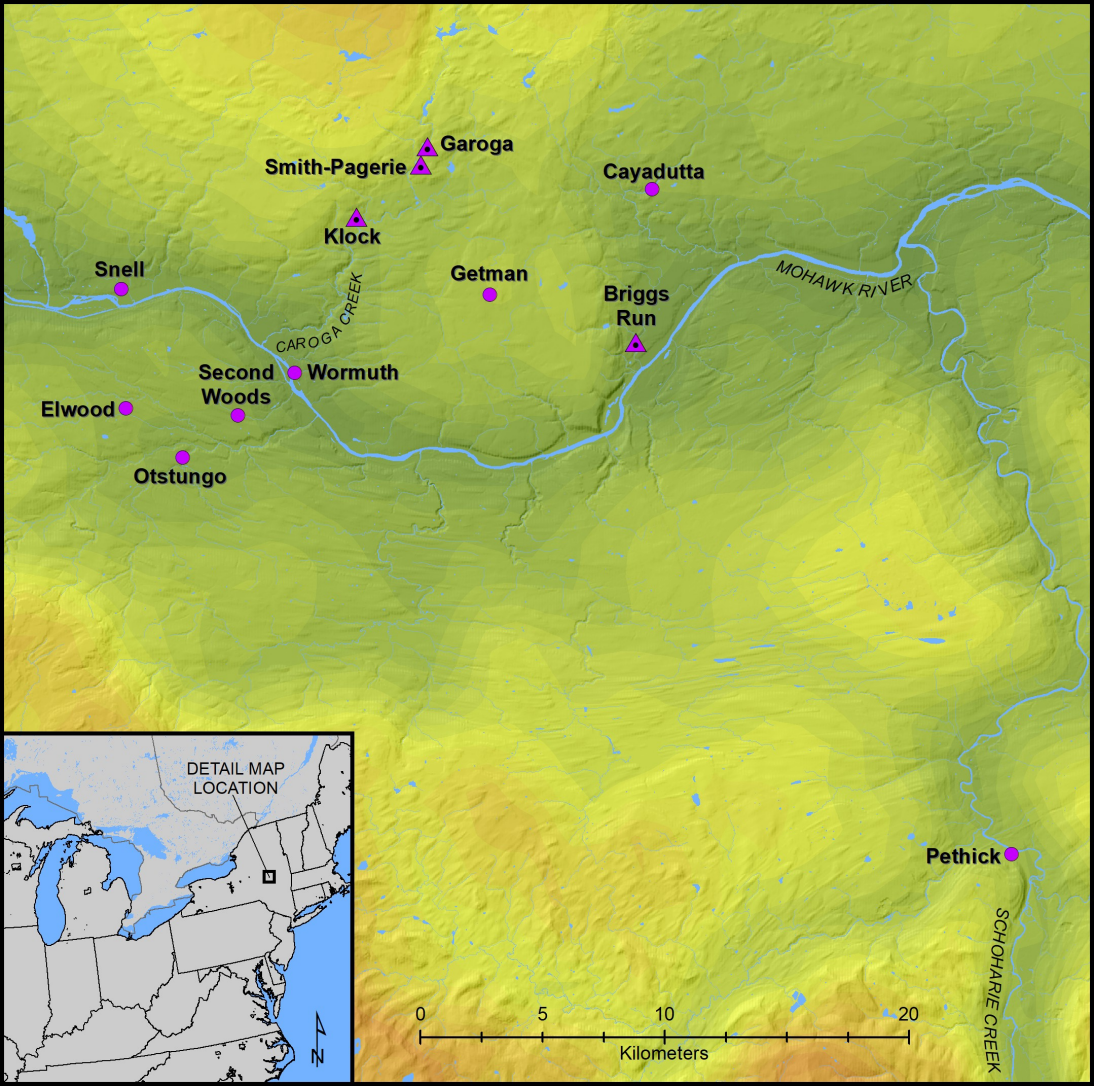
A map showing the Mohawk Valley region in northeast North America and showing the four sites analyzed in this study (Brigg’s Run, Garoga, Klock and Smith-Pagerie shown as triangles) and another eight sites re-dated in this paper and revising previous published work [65]. This map was produced in ArcGIS v 10.6 at the New York State Museum in Albany by compiling GIS shapefiles obtained from open access, publicly available, sources including Statistics Canada, the United States Census, and the United States Geological Survey.

The Mohawk River Valley in eastern New York forms one of the most intensively studied areas of Indigenous settlement in northeastern North America. The name refers to the association with the Kanienʼkehá꞉ka, or Mohawk Nation, one of the original Five Nations of the Haudenosaunee (Iroquois) Confederacy [25, 29, 70]. This Mohawk region is also then associated with the area occupied, after initial exploration in 1609, by the Dutch (from 1614 until it passed into British hands in 1664) [11, 12, 69]. Archaeological excavations in New York in the 1960s and 1970s (e.g. [36, 71–75]), provided information on a number of sites in the region and formed the basis of considerable further work since—including the first substantial AMS ^14^C dating effort in the region [39]. Notable was the work at what became three iconic village sites excavated by crews from the New York State Museum. Located in the Caroga Creek drainage, a tributary of the Mohawk River, the Garoga, Klock, and Smith-Pagerie sites are the most extensively excavated ancestral Mohawk village sites to date [72, 76]: Fig 1.

These three sites had been subjects of avocational excavations and collecting during the 19^th^ and 20^th^ centuries. Results of the excavations at Garoga were first summarized by Ritchie and Funk [72]. Funk and Kuhn [76] provided extensive overviews and analyses of the sites, including revised interpretations of the village plan of Garoga. Ritchie and Funk [72] suggested that the three sites were a sequence of villages occupied by the same community during the 16^th^ century. They did not suggest a chronological sequence for the sites, although based on the amount of European-provenance metal found at Smith-Pagerie, they thought it might be more recent than Garoga. They proposed the sites could not have been occupied contemporaneously because one interpretation of ethnohistoric data suggested the Mohawk River basin was divided between clan territories, and they assumed that only three or four villages were contemporaneously occupied in the (wider) valley ([72] at p.332). Additionally, they believed that resources in the Caroga Creek drainage would not support more than one village community at a time. “It seems unlikely that the two sites [Garoga and Smith-Pagerie] coexisted for any appreciable period, as the drain on local resources of game, firewood, and soil fertility would have been considerable” ([72] at p.332).

Since Ritchie and Funk’s publication, the three sites have been considered a single community sequence during the 16^th^ century (e.g. [77]). Based largely on pottery type and European trade metal frequencies, efforts were made to place the three sites into a chronological sequence. Funk and Kuhn [76] performed formal frequency seriation analyses using pottery attributes that they believed were chronologically sensitive. Consistent with Snow’s [39] and earlier interdigitized seriations of Mohawk Valley sites including all or two of the Caroga Creek sites [45, 77, 78], Funk and Kuhn ([76] at p.142) concluded the order of habitation (from oldest to most recent) was Garoga, Klock, and Smith-Pagerie (cf. [79]). Conversely, recent results of a Bayesian analysis of a large suite of new AMS ^14^C dates on maize and white-tailed deer bone from 12 key Mohawk Valley sites, suggested a habitation sequence (oldest to most recent) of Smith-Pagerie, Klock, and Garoga with the Smith-Pagerie occupation initiating in the last two decades of the fifteenth century [65].

A social network analysis of Mohawk Valley sites meanwhile demonstrated that the three sites are in fact unlikely to represent a single community [80]. The results of this analysis suggested that ancestral Mohawk settlement systems were more flexible than previously proposed, with multiple communities able to establish villages outside of any clan-based landscape divisions (see [50] for a similar analysis of contemporaneous sites in southern Ontario). Given the importance of these sites in understanding ancestral Mohawk landscape use and history, it is therefore imperative to (i) ascertain if the results of the earlier Bayesian chronological analysis are robust; and (ii) attempt to better resolve the chronology of these three iconic sites. To that end we investigated the archaeological excavation and laboratory records from these three sites in detail and obtained additional radiocarbon dates from them, including dating short tree ring sequences, to obtain *terminus ante quem* (TAQ), *terminus ad quem*, and/or *terminus post quem* (TPQ) dates for each of the sites and refine the Bayesian models. We further obtained new dates on the Brigg’s Run site (Fig 1), which, based on the extensive material recovered, should be largely contemporaneous with the historically documented early Dutch period [39], both as a test of dating, and to aid resolution given there were few ^14^C dates available in the earlier Bayesian study [65]. Our results considerably refine the date ranges of the four sites. When considered in conjunction with the data from the previous study [65], they provide support for the earlier-modelled chronological order of site habitation and offer a highly time-resolved chronology for settlement and history in the region.

### Defining space and time in Northeast American archaeology–history of work

Since the 1960s a major theme of regional archaeological studies has been the reconstruction of settlement patterns—how contemporaneous sites of various functions were distributed across the landscape, and of settlement systems—how activities at the various sites formed networks of function and interaction [81, 82]. Subsumed today under the rubric of landscape archaeology (e.g. [83]), these fundamental concepts have guided regional archaeological research for decades. Key is establishing both contemporaneity and distinctions of sites across the landscape both to reconstruct contemporaneous patterns and systems and modeling how those patterns and systems changed over time.

From the early twentieth century, archaeologists in North America developed various methods and techniques to control for the passage of time. These approaches formed at a time when there was little understanding of time depth of Native American occupations in North America (e.g. [84]). Methods and techniques such as frequency seriation and percentage stratigraphy, based strictly on archaeological artifact assemblages, most often pottery [85], tried to create order among site datasets. Combining seriations from multiple sites through interdigitation, it was possible to create regional relative chronologies [85], although the temporal scale was not known (and therefore the validity of the ordering only assumed, not demonstrated). Coincident with this was the development of regional culture historical schemes with the goals of controlling and bounding temporal and spatial variations in artifact assemblages [86, 87]. Contemporaneity was determined by assignment to the same taxonomic unit (artefact category). Because a goal of culture historical taxonomic systems was to minimize variation within taxa, change could occur only at taxonomic boundaries. Taxonomic units (developed regionally) such as Willey and Phillip’s [87] phases, became the units of analysis in attempts to build understandings of the past and were frequently, mistakenly, identified as the equivalent of ethnic groups [88]. However, this is evidently not an issue in the current case given the continuity in the archaeological record in the Mohawk River basin from the earliest time under consideration here to the early 17^th^ century and the historical identification of this region with the Mohawk Nation. However, below this general level, both social network analysis and direct dating via radiocarbon in several cases further challenge attempts at more refined assumed associations and chronological placements solely from such material-ethnic associations [50, 62–65, 80].

The advent of radiocarbon dating and its archaeological application beginning in the late 1950s provided a means, independent of the assumptions derived from the archaeological record or other sources, to establish chronologies directly from the dating of organic material selected from the sites or objects of interest [89]. In early applications one or a few radiocarbon dates were used to anchor seriations and taxa in time, while culture historical taxa continued to be the units of analysis, summary, and narration in spite of prominent critiques of the practice (e.g. [90]). The continued development and refinement of radiocarbon dating and its expanded use in archaeology over the intervening decades provides new opportunities to independently control regional chronologies for landscape research. Dendrochronological calibration of radiocarbon dates to account for fluctuations in atmospheric radiocarbon concentrations; accelerator mass spectrometry (AMS) dating, allowing analysis of small samples while increasing both accuracy and precision of dates; and, as noted above, Bayesian analyses of large suites of radiocarbon dates have all combined to make the building of site and regional chronologies less subjective and more robust in recent decades (e.g. [58–61, 91–94]). While widespread in several areas of world archaeology for over two decades, applications of these developments to the 15^th^ to 17^th^ century Indigenous archaeological record in eastern North America is only in its initial stages (e.g. [95, 96]). The largest concentration of work so far has been in present-day New York (USA) and Ontario (Canada), focusing on the northern Iroquoian region (e.g. [62–65, 97]).

Research on Native American settlement patterns in present-day New York was carried out in two extensive research programs in the 1960s through 1980s [72, 74]. These research programs sought to understand settlement patterns over the full chronological extent of Native American occupations of the state. Critical to both was the use and establishment of chronological control. Ritchie and Funk [72], whose research encompassed most of the state, relied primarily on their culture-historic framework combined with interpretations of the few available radiocarbon dates (which at this time yielded at best relatively wide and only approximate age estimates—these authors were also operating before the general adoption of tree-ring calibrated radiocarbon dating [89]). Funk [74] focused on the upper Susquehanna River basin and also framed his settlement pattern analyses in the culture-historical framework but made a concerted effort to obtain large series of radiocarbon dates to bolster the chronological placement of sites within the culture historic framework.

Snow [39] conducted more chronologically constrained research focused on the late prehistoric to early historic Native American occupations (ca. AD 900–1776) of the Mohawk River basin. This research included the first large-scale AMS ^14^C dating of annual plant remains (maize) in northeastern North America. Combined with traditional relative-dating practices, including pottery type frequencies and the presence, amounts, and categories of European exchange items, this work enhanced understandings of the basin’s occupational histories, work that was later critiqued and clarified by Lenig [79].

An initial Bayesian modelling investigation of a set of Mohawk River Valley sites challenged several of the conventionally assigned dates for sites [65]. This was the first effort to build a chronology for ancestral Mohawk sites entirely independent of traditional approaches to chronological control based on artifact analysis. However, in several cases, and in particular for the important sites of Garoga, Klock and Smith-Pagerie, the available data were less than perfect, and some ambiguity remained (and for Brigg’s Run there were very few ^14^C measurements). Thus, in this present study, we have obtained a number of new radiocarbon measurements on samples from these four sites (S1 Table), and, in particular, as a methodological development, we have focused on available wood-charcoal samples offering even short, annually-defined tree-ring time series ending in waney edge/bark—whose dates correspond with the wood’s cutting and use by humans. We then employ tree-ring sequenced ^14^C ‘wiggle-matching’ on these samples to obtain more precise calendar date estimates for these episodes [66–68, 98]. For Garoga, Klock and Smith-Pagerie we were able to identify samples which can be tied closely to specific points in each site’s stratigraphic record based on re-examination of the original records from the excavation of each site (see Methods, S1 File). In several cases, on careful examination of these original site records, we identified wood charcoal from contexts which appear associated with the late, final, or even likely post-occupation use of the relevant feature, and likely the overall site, that provide therefore *terminus ante quem* (TAQ) constraints for the main site occupation phases of interest. Our aim was to achieve chronological resolution for each of these four sites in isolation—rather than from assumed site sequences or approximate dates—employing the ^14^C data from each site integrated with the prior information from site taphonomy and ethnohistoric and archaeological observations. This work permits us to resolve the date of each of the sites closely and clarifies their temporal relationships. We then further re-considered the chronological placements and relationships between these sites and a wider set of Mohawk River Valley sites (Fig 1).

## Materials and methods

### Organic sample identification

All wood charcoal fragments larger than 2 mm were fractured by hand or with a steel razor blade to create fresh transverse, radial, and tangential planes, in order to examine the wood anatomical structure and identify the taxon. After fracturing, wood samples were supported in a sand bath or modeling clay and examined under a Motic K-400P stereo microscope at x6–x50 magnification and an Olympus Bx51 polarizing microscope at x50–x500 magnification. Seeds were examined under the same set of microscopes. The macro- and micro-anatomical features of wood sections and seed samples were documented, photographed, and compared with those from modern reference collection materials in the Cornell Tree-Ring Laboratory, standard reference texts [99, 100], and the InsideWood (http://insidewood.lib.ncsu.edu) and USDA Plants (https://plants.usda.gov/) online databases. A LEO 1550 field emission scanning electron microscope (FESEM) was used for high magnification observation of anatomical micro-features and high-quality image capture.

### Radiocarbon samples, dating, and site contexts

The ^14^C dates employed are either new dates, all UCIAMS, published here (S1 Table), or previously published dates [39, 65]. No permits were required for the described study, which complied with all relevant regulations. The new UCIAMS samples were analyzed by the W.M. Keck Carbon Cycle Accelerator Mass Spectrometry Laboratory (KCCAMS) at the University of California-Irvine [101]. At KCCAMS, bone samples were decalcified in 0.5N HCl, gelatinized at 60°C and pH 2, and ultrafiltered to select a high molecular weight fraction (>30kDa) [102]. Charcoal and maize samples were subjected to the standard acid-base-acid (1N HCl and 1N NaOH, 75°C) pretreatment. δ^13^C values were measured at KCCAMS to a precision of <0.1‰ relative to standards traceable to PDB with a Thermo Finnigan Delta Plus stable isotope ratio mass spectrometer (IRMS) with Gas Bench input. Details on KCCAMS dating protocols are available on the facility’s website (https://www.ess.uci.edu/group/ams/facility/ams).

The new samples for dating from the Garoga, Klock, and Smith-Pagerie sites were recovered from hearths or large pit features. The size and shape of the large pit features (S1 Table and S1 File) and in many cases the presence of charred or partially charred grass and/or bark linings suggest their original function was storage of agricultural crops [103]. The linings and any crop macrobotanical remains located on and/or immediately above the linings most likely represent the original uses of the pits. Therefore, radiocarbon dates on these elements are likely to represent time during each feature’s original period of use. Fill above these elements represent subsequent activities including purposeful filling episodes, fill from sheetwash and erosion of pit walls, as well as subsequent secondary use [103, 104]. Unburned bone recovered from hearths is unlikely to have been deposited while the hearths were in primary use. As such, radiocarbon dates on material recovered from these known pit contexts represent *terminus ad quem* for the pits’ original functions. We focused especially on identifying wood charcoal samples from specific loci with a number of tree-rings preserved out to original waney edge/bark. The terminal tree-ring in these cases therefore provides a close date estimate for the human use of this sample in the episode represented in the context. The availability of a series of tree-rings leading to this event means that we can employ the ^14^C ‘wiggle-matching’ approach [66, 67] to constrain and better define the likely date. Even short-sequence wiggle-matches can usually usefully inform chronological analysis [63, 64, 68, 98, 105].

We note that cases of reported inaccuracies for short-sequence wiggle-matches at high-precision [106, 107] are in fact relatively minor in scale in most cases and typically occur when the time-intervals of the samples dated (i.e., the specific number of tree-rings dated, and so calendar window comprising the dated samples) are much shorter than the integrated (smoothed) IntCal ^14^C Calibration curve resolution available. Inaccuracies may also occur at certain periods when the total length of the time series is sufficiently short that it may not be able to permit discrimination between multiple periods with similar atmospheric ^14^C levels and trajectories (i.e. reversals or wiggles in the calibration curve). Over the last 1000 years, such parallel instances of similar ^14^C levels and trajectories are typically distinguishable once time-series are greater than ~50 years. The latter issue of potential ambiguity is potentially relevant in the context of this article especially to the periods around ~1490–1535 and ~1600–1635. In such cases, additional constraints may be necessary to achieve satisfactory discrimination for very short time series. The former issue applies when, for example, single, annual, tree-rings only are dated (potentially capturing high-frequency annual-scale variation in atmospheric ^14^C—attested in available annual resolution ^14^C time series: e.g. [108–110]), whereas the calibration record, while largely based on annual data for the second millennium CE, is in fact used as a smoothed curve removing very high-frequency (i.e., annual-scale) variation [111, 112]. For this reason in our study, we sought to date samples comprising several tree-rings in most cases where possible (5 rings in six cases, 2 or 3 rings in 4 cases each, 6 or 9 rings in 2 cases, and a single ring in just 2 cases: see S1 Table), in order to damp-down any very high-frequency variation and so achieve data likely more similar to the smoothed IntCal curve. Nonetheless, we suggest that we do observe an issue in the case of the Klock *Ulmus* sp. wiggle-match (see below and Fig 3). Here short-term variation or another offset may apply. The consistency of results across different analyses of the same/similar samples in this case suggests perhaps the relevance of real short-term higher-frequency atmospheric variation–especially noticeable in the relevant mid-later 16^th^ century period where there is a plateau or moderate reversal in the calibration curve accentuating any ^14^C offset/difference. As noted by [113], any instances of inter-laboratory variation/offsets will further exacerbate any such problems at high-precision and where only a short sequence with limited data is wiggle-matched. This observation suggests that care needs to be taken with high-resolution dating in the mid-later 16^th^ century, especially if considering annual resolution samples or short sequences. But, in our specific case, it is important to observe that this apparent high-frequency variation (or other offset) is *not* occurring at a time where there is a potential alternative calendar placement (contrast the case if the series was lying around either ~1490–1535 and/or ~1600–1635 when there could be an ambiguity). Instead, the mid to later 16^th^ century calendar placement for this Klock time series is the only available placement for the series regardless of the evident variation, noise or offset.

Tree-ring sequences are described in terms of Relative Years (RY): the first measured (innermost) tree-ring (after pith, if present) is RY 1001. In this case we sought to have more than one such sequence for each site with the view that achieving multiple compatible results reinforces confidence in the dates obtained. The samples from Brigg’s Run, in contrast, are from an avocational archaeologist’s collection donated to the New York State Museum with minimal associated documentation. Thus we make no intra-site history assumptions in this case. The key issue for this site, rich in European trade goods including types that, according to all understanding, can only date from the early 17^th^ century ([39] at pp.252-259), is whether the ^14^C dates are consistent with placement in the first decades of the 17^th^ century.

In this study, aimed at high-resolution dating of the Garoga, Klock and Smith-Pagerie sites, we were specifically interested in whether samples reflected original functions or subsequent fill, and, if subsequent fill, whether this could be related to the closure or end of use of the feature and likely the wider site occupation, or even post-occupation activities. To that end, site-specific excavation and laboratory records in the New York State Museum’s archaeological archives were consulted to determine the origin of samples dated from within the various pits (S1 File). Written records, often made by field-school students, were of varying quality and detail. However, profile drawings combined with these notes made it possible in some cases to either determine specifically where samples were recovered or their most likely location of recovery within a feature’s fill. Determinations of likely location of recovery are provided in S1 Table with discussion and additional information in S1 File. Here we provide descriptions of the pits from Garoga, Klock, and Smith-Pagerie from which the wood charcoal samples we selected for dating were recovered.

Garoga site Feature 36 was a cylindrical pit measuring 122 cm in diameter and 147 cm deep within Longhouse 9 ([76] at p.99). The pit’s profile indicates a complex series of fill units (S1 File). Field notes indicate that a “lobe” of fill was present at the top of the feature along the south edge. This fill contained fire-cracked rock, charcoal, ash, and a few sherds. This is the only fill unit from which charcoal is specifically mentioned and is the most likely source of the charcoal samples. We therefore regard this context as after the main use of the pit–a *terminus ante quem* (TAQ) for the earlier use and for the site as a whole.

Klock Feature 84 was a large pit measuring 107 x 152 cm in plan and 122 cm deep (S1 File) within Longhouse 1 (or House 1 = H1). A sample of the charred grass lining was taken from the bottom of the pit, which was AMS dated for the current project (UCIAMS-239714). We regard this lining sample as dating the construction or start of use of the pit. Immediately above the grass lining was a lens of charred maize including maize cobs with kernels attached. Maize kernels (UCIAMS-239713, UCIAMS-218474) and a maize cob fragment (UCIAMS-239712) were AMS dated. These samples, all subsequent to the lining, date the primary use of the pit. The charcoal samples most likely come either from a small lens labeled “burned wood” or a thin lens near the top of the pit that indicates charcoal. We therefore interpret the date of the waney edge of the charcoal sample as defining the closing use/end of use of the pit. It is thus a TAQ for the earlier use of the pit and for House 1 generally—and likely for much of the site occupation, since such deliberate final pit closure is likely from the end of use/abandonment of the site. Indeed, given that the cutting and use date available for a sample of this charcoal (see below) appears to be a few decades at least after the rest of the Klock dating evidence, there is reason to suspect that this charcoal was in fact from the lens right at the top of the Feature 84 pit and so may in fact even post-date the main Klock site occupation period.

The maize sample dated by ISGS-A0326 needs comment. This sample was reported in [65] as coming from Feature 84. This is incorrect. It is in fact sample 45157-A and is from Feature 50. Feature 50 is the other pit within Longhouse 1 with a large maize deposit. However, unlike Feature 84, the maize deposit in the Feature 50 pit is not from the bottom (and first or early use) of the pit, and so likely represents use and fill of the pit after it was no longer serving its primary purpose. Hence we assume that this maize deposit reflects later use of Longhouse 1, in contrast to the initial/earlier use represented by the material from the bottom of the Feature 84 pit.

Klock Feature 135 was a pit measuring 123 cm in diameter located between Longhouses 3 and 5. Field notes indicate there was a lining of charred “vegetal material” at the base of the pit in which maize was present. This is the only mention of maize in the field notes and is the likely source of the AMS-dated maize kernel (UCIAMS-218475) reported in [65] representing the original function of the pit. Above this lens was a massive deposit of large fire cracked rocks, which filled the pit. The field notes do not mention charcoal samples, but it is likely that the charcoal AMS dated for the present project came from the fire-cracked rock deposit—wood charcoal is not mentioned in the description of the bark lining.

Smith-Pagerie Feature 54 was a pit measuring 122 x 152 cm in plan and 135 cm deep within Longhouse 1. A dark, charcoal-stained lens extending across the pit at depths of 63.5–72.4 cm, 61 cm above the bottom of the pit, contained abundant maize. A maize kernel from this lens was AMS dated (UCIAMS-218490) as reported in [65]. AMS dates on two maize kernels also apparently from this lens (AA-6419, AA-7405) were reported by [39]. Field notes indicate that charcoal samples selected for dating were recovered from the same lens as the maize. The pit profile drawing suggests that the upper half of the pit was filled in, partially re-excavated, and refilled (S1 File). This second fill was evidently excavated into and then refilled. The dark lens was at the base of the first posited re-excavation. A 19^th^ century source indicates that pits were visible at sites like this at the time and would presumably therefore have been visible to ancestral Mohawk people after having been filled ([114] at p.23): “These strange aboriginal magazines or storehouses can be seen in numbers even at the present day. Some of them are in the dense forests, others lie in open fields. They are looked upon with curiosity by the country people who regard them as the graves of Indians.” Such visibility, and the knowledge of the presence of these features (with differing Indigenous versus European/settler interpretation) may well have encouraged at least occasional activity at these loci—potentially for very different reasons (Indigenous versus European/settler)—which could explain instances of apparent late or post-use pits and high-up and near plow-soil charcoal material. We therefore interpret the samples we date as belonging to activity or activities ending with the firing represented by the date of the waney edge of the charcoal samples (thus a TAQ for the earlier use of the pit) and latest maize samples, with some older material likely from earlier (to initial) use of the pit.

Smith-Pagerie Feature 60 Longhouse 5 was a small and relatively shallow pit (36" diameter and only 24" deep: [76] at p.61). Thus, we assume that this pit likely represented just one real use episode. We therefore assume the maize dated (UCIAMS-218493) gives a date for the first use of the pit. The ^14^C however is much older than any other dated element from the site (even treating this as an initial site context). It is a ~98% probability outlier if assumed to be the first date from the site, and a 100% probability outlier if included in the subsequent main site Phase. This date is thus excluded from the dating models, whether as an unexplained outlier, or evidence of distinct and earlier human activity at the site locus.

### Radiocarbon modelling

We employed the OxCal Bayesian chronological modelling software [92] using version 4.4.4 with the IntCal20 Northern Hemisphere ^14^C calibration curve [111] (curve resolution set at 1 year). OxCal key words/commands (Chronological Query Language, CQL2: see https://c14.arch.ox.ac.uk/oxcalhelp/hlp_commands.html), such as Boundary, Date, Before, D_Sequence, Gap, Interval, LnN, Outlier Model, N, Phase, R_Combine, Sequence, are capitalized in our text. Individual outliers within site models were identified and down-weighted using the OxCal SSimple Outlier model within D_Sequence (tree-ring wiggle-match) sequences [66] and with the OxCal General Outlier model in the case of short-lived samples [115]. Dates on wood charcoal (not part of wiggle-matches) were analyzed with the OxCal Charcoal Outlier model applied to approximately allow for the likely in-built age involved in the dated sample [115]. The SSimple Outlier model was also used to assess weighted averages against the model. Samples on the identical material, including the same exact tree-rings, were combined following [116]. Samples are identified in the figures as M = maize (*Zea mays* ssp. *mays*), B = animal bone, and R = residue (food/organic residue). Other samples are given descriptions. Each site is modelled within an overall site OxCal Phase between start and end Boundaries. Based on ethnohistoric information we regard the occupation of each village as likely being within a total range of 0–30 or 0–40 years with average site occupation lengths somewhere likely in the range of 10–20 or to 25 or so years [62, 68, 117] (and see further discussion in Results below).

**Fig 2:**
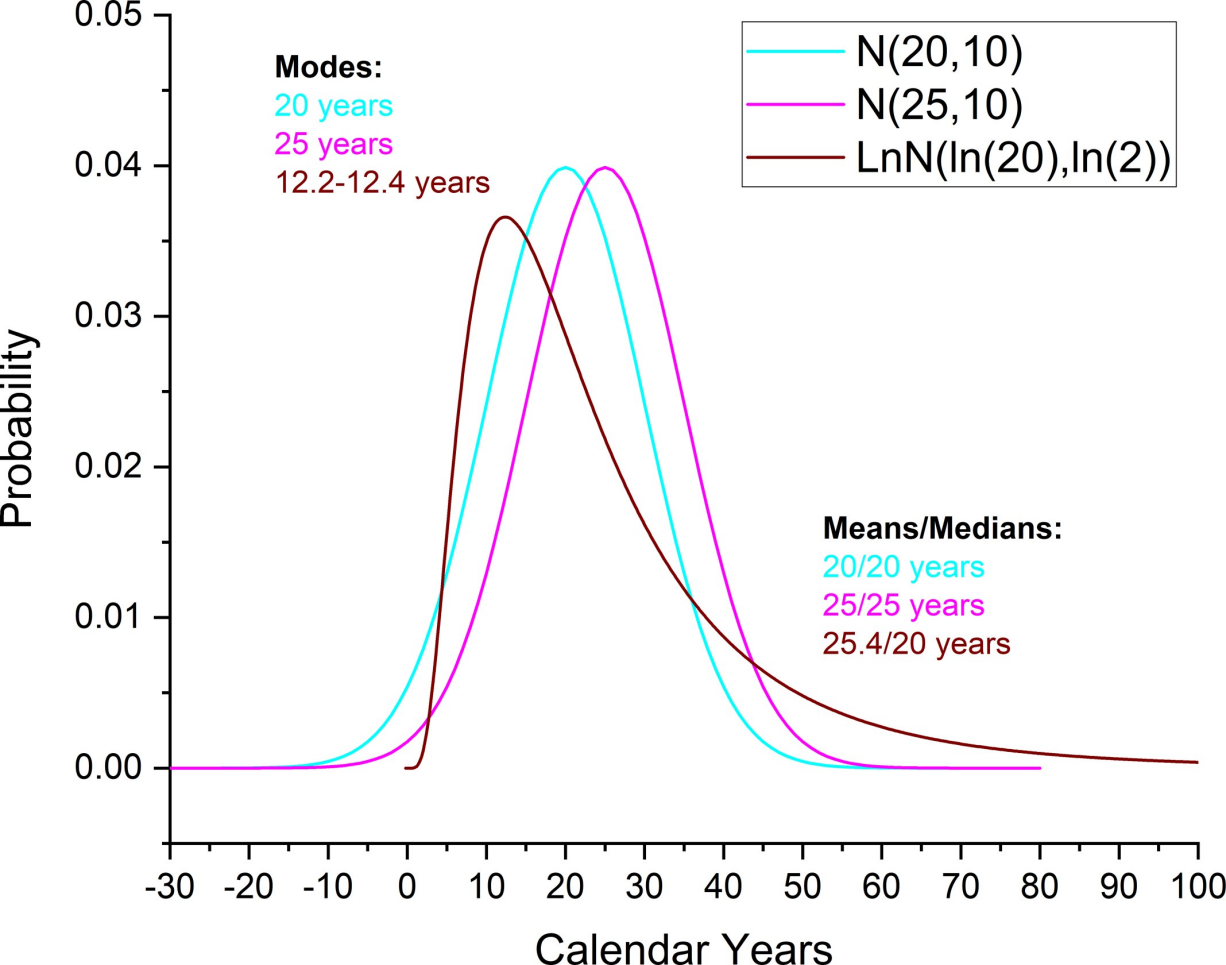
Visualization and comparison of the three different site duration constraint probability distributions used and applied in the different models via an interval query, constrained by respectively normal distributions of N(20,10), or N(25,10), or a Log Normal distribution of LnN(ln(20),ln(2)).

We therefore consider three constraints on the duration of each site between the start and end Boundaries via an Interval query with a constraint of (i) a Log Normal distribution of LnN(ln(20),ln(2)); or (ii) a Normal distribution of N(20,10); or (iii) a Normal distribution of N(25,10). In each case the site Model 1 uses the LnN constraint; the site Model 2 uses the N20,10 constraint; and the Model 3 uses the N25,10 constraint (see Results and S2 Table). Critically, the LnN or N constraint does not impose a hard upper limit; thus the dates from the site can overwhelm the prior if the assumption is not appropriate and so indicate a problem. In all cases satisfactory agreement is evident between the Posterior v. Prior distributions (OxCal Agreement values >60). Fig 2 shows and compares these three site duration probability assumptions. All have similar mean and median values (between 20 and 25 years), reflecting the ‘average’ site durations expected based on assessments of ethnohistoric and archaeological evidence [62, 68, 117]. The two Normal (or Gaussian) distributions then symmetrically allocate declining probability around these average/median values (of 20 and 25 years)—95.4% of the probability lies between 0–40 or 5–45 years, respectively. In contrast, the LnN distribution reflects an assumption that in the real world the Normal distribution may not be appropriate in a case like this. While the median or average lifetime of villages (as a set) may be around 20–25 years, many villages likely had a shorter lifetime (the mode value for the distribution used is between 12.2–12.4 years), just as some (few) villages may have had lifetimes that are longer, and a very few even much longer, than the ‘average’. The LnN distribution better accommodates this real-world range—95.4% of the probability lies between 2.36–65.04 years. In practice, however, the modelled results are relatively similar using all three models (since they are describing reasonably similar scenarios) [68] (see Results). We explain each of the four site contextual histories and their models and any specific site assumptions below by site.

#### Brigg’s Run

The ^14^C dated samples all from the site Phase–with no further information available as the material derives from a non-systematic avocational collection given to the New York State Museum–comprise four dates on short-lived samples (maize) belonging to the site Phase, and a wiggle-match series of dates on a 25-ring beech (*Fagus grandifolia*) sample ending in bark, reflecting a use date somewhere within the site Phase. There is relevant historical information ([39] at pp.252-259). The account of Van den Bogaert [118] states that by the time of his inspection visit in 1634–1635 there were only villages on the south side of the Mohawk River. Thus, this gives a *terminus ante quem* (TAQ) for the end of Brigg’s Run (on the north shore of the river) by 1635. Less definite but informative, is the rich assemblage of a range of materials recovered from Brigg’s Run which can only belong in the early 17^th^ century, with some items indeed likely only post-1614 (and the founding of Fort Nassau) and even post-1624 –though materials likely dated from the years 1626–1635 are not specifically observed in Snow’s [39] review. Overall possible dates are thus stated as within the range ca. 1600–1635 ([39] at pp. 252–259). Other scholars generally agree, but some place the date towards either the earlier or later portions of this overall range. For example, Rumrill ([119] at pp.9-10) suggests 1600–1615 whereas Bradley ([11] at pp.74, 80, 199 n.35) suggests 1624–1635. Such material culture-based assessments necessarily are approximate but are likely relatively robust when based on a range of material types and on multiple examples. One area of error or caveat could be that the European-dated material arrived at the site well after its initiation (rather than from the start). To be highly conservative, we therefore consider a possible 100% error on these expert assessments. Thus we regard the possible date range for the site as somewhere–with uniform probability–between 1565 to 1635 by limiting the start and end Boundaries for the site Phase with a constraint of U(1565, 1635). While leaving a liberal range available of ca. 70 years, this has the important effect of removing what would otherwise be possible dating probability in the ca. 1500–1560 region (due to calibration curve taphonomy)—but is clearly not plausible given the good historical associations with the early 17^th^ century.

#### Garoga

As noted above, there are two wood charcoal samples evidently from a late high lobe on the south side of F36 pit. Wiggle-match dates for the waney edge of a beech (*Fagus grandifolia*) sample and the last extant tree-ring from an elm (*Ulmus* sp.) sample are interpreted as providing a TAQ for the pit and so for Longhouse (House, H) 9 at the site (with the date on the waney edge placed as the latest available evidence). There are seven other dates from House 9. Y-1381 is wood charcoal that may well include substantial in-built age, which we accordingly model applying the OxCal Charcoal Outlier model. AA-8370 on maize from F37 pit is a 100% probability outlier versus the site model–and is too old–for unexplained reasons. Either it is an erroneous measurement or represents previous, much older, activity at the site area. The other dates (all on maize) come from the F2 pit. AA-7695 is a ca. 10% probability outlier in the model (whose date is slightly too old). We exclude AA-8370 and AA-7695. An intra-site Sequence for House 9 is modelled placing the data from the House before the wiggle match TAQ dates. A Difference query applied to the period between the start and end Boundaries for House 9 yields estimates for the duration of House 9 of 0–8 years at 68.3% hpd, and 0–21 years at 95.4% hpd. Other dates from Garoga come from pits in House 2, House 4, House 5 and House 12 and from F184 pit between House 1 and the stockade. We model the overall site Phase.

#### Klock

A small diameter elm (*Ulmus* sp.) branch preserving the entire stem from pith to bark with 25 rings (the last including latewood formation) came from what appears to be the last and final use of the Feature 84 pit–high-up in the pit just below plow-soil–and this last use is likely either approximately contemporaneous with, or after (potentially even well after), the last use (post-closure) of the associated Longhouse (House, H) 1 structure–since Feature 84 was under the area of a bed platform during the use of House 1 and thus this final activity in Feature 84 probably occurred after the removal of House 1. Thus the date on the waney edge of this sample offers a TAQ for House 1. The length of the ‘ante’ is not known. A tree-ring sequenced wiggle-match is available. Two ^14^C dates are on relative years (RY) 1001–1002 (innermost two rings of the sample: UCIAMS-226653 and 239720). Since these dates are on the identical tree-rings (and are very similar) we combine them as a weighted average. Another ^14^C date is on RY1010-1018 (UCIAMS-239721), ~12.5 years later, and a final ^14^C date is on RY1021-1025 (UCIAMS-226654), 9 years later again. The date of the last complete tree-ring (including the waney edge), RY1025, is 2 years after the mid-point of the RY1021-1025 sample.

**Fig 3:**
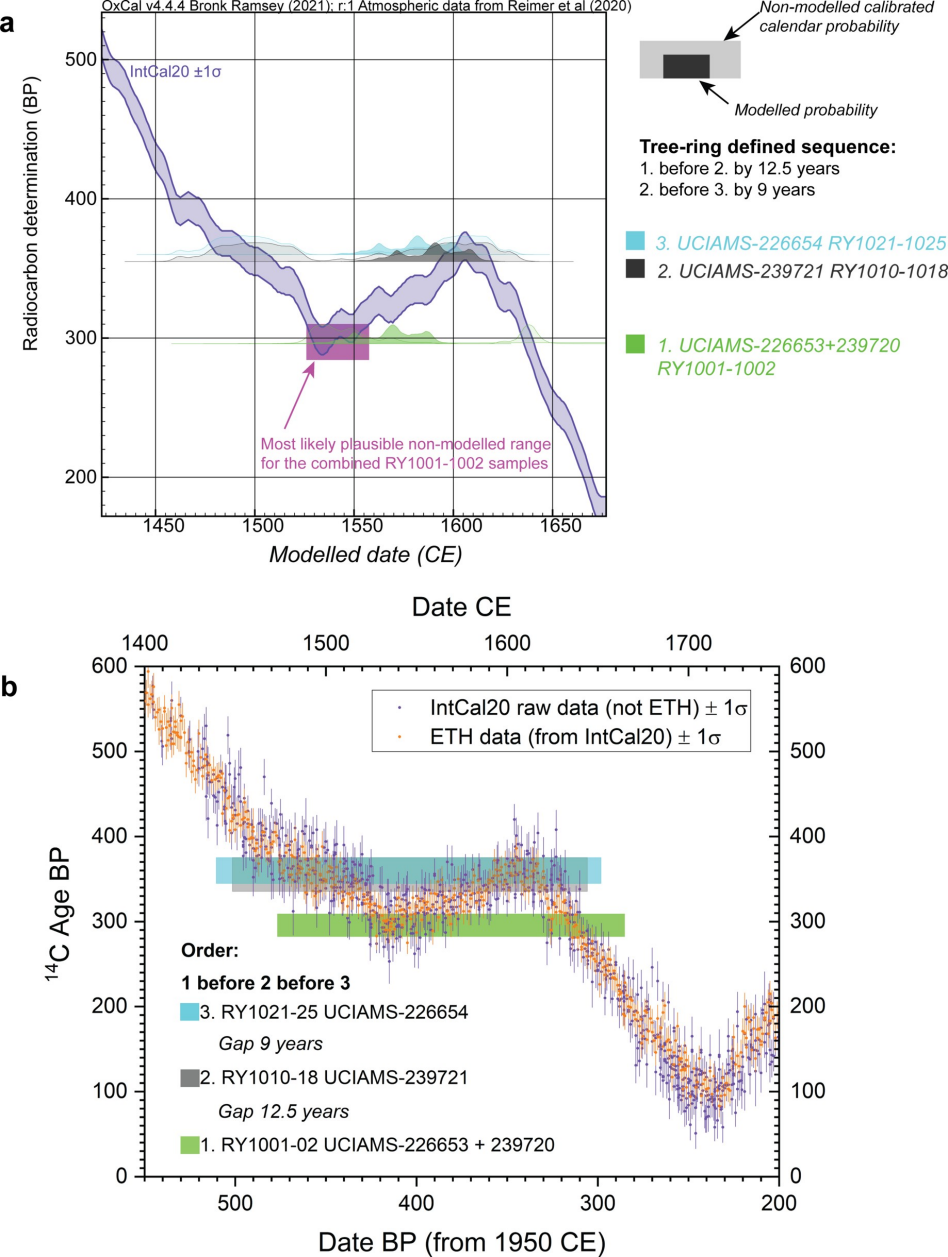
Relationships of the ^14^C dated tree-ring sequenced samples from the Klock Feature 84 *Ulmus* sp. branch with IntCal20. **a**. The laboratory stated data wiggle-matched against IntCal20. Two measurements for RY1001-1002 are combined as a satisfactory weighted average and there are then dates on RY1010-1018 and RY1021-1025. The likely non-modelled calibrated locations do not accommodate the known tree-ring spacing in detail. The two dates on RY1001-1002 identify 1527–1552 as the most likely placement (purple box). But this is not compatible with the other two dates given the tree-ring spacings (hence the expected, if the likely non-modelled dates on RY1001-1002 are correct, pink box is left floating in free space away from the IntCal20 curve). The wiggle-match, trying to accommodate all the dates therefore moves the modelled RY1001-1002 dates (solid green distribution) to a more recent placement (largely missing IntCal20 and hence achieving a poor OxCal Agreement value: 44.6<60) but allowing the other modelled dates (solid gray and solid cyan distributions) to be associated with IntCal20 (although the overall Amodel value is poor: 31<60). **b**. The raw IntCal20 dataset from which the modelled curve in **a**. is derived [111] highlighting the ETH dataset [108]–these raw data indicate greater possible variation which might allow the observed Klock data to fit better, including earlier, similar to the purple and pink boxes in **a**.

The wiggle-match is not entirely straightforward as the ^14^C dates on these samples offer a less than perfect fit against the calibration curve (see Fig 3A). The two dates on RY1001-1002 are very similar and the combined weighted average value yields a (non-modelled) specific date either 1527–1552 (50.6% probability of the 68.3% hpd range) or much later 1633–1642 (17.7% probability of the 68.3% hpd range). This suggests somewhere around 1527–1552 as the likely (plausible) age. The last ring should therefore be less than 24 years later: *before* 1551–1575. However, the other two dates indicate non-modelled ranges (ruling out impossible options *before* the RY1001-1002 date) later, either 1592–1619 (UCIAMS-239721) or 1580–1622 (UCIAMS-226654). The net outcome versus IntCal20, adding in the constraints of the tree-ring derived spacing between the samples and trying to compromise what appear to be somewhat non-compatible data, is that the wiggle-match is pushed later with the RY1001-1002 weighted average placed most likely 1564–1589 (59.9% of 68.3% hpd) *but* at the price a poor OxCal individual Agreement value of ~44.6 < 60, since the modelled placement no longer corresponds with most of the likely non-modelled calibrated probability. At first sight, this result appears of concern. But it is important to consider that the IntCal curve is a narrow, modelled probability band trying to best-describe a large set of raw ^14^C calibration data available in this period. For example, one high-quality annual resolution time series of recent data (ETH dataset) shows considerably greater structure in the 16^th^ century than represented in the smoothed IntCal20 record [108] (see Fig 3B). Overall, comparison of the ^14^C age ranges of the Klock branch samples versus the raw IntCal20 dataset suggests greater potential flexibility. If we consider just the wiggle-match of this *Ulmus* sp. branch, the OxCal A_model_ and A_overall_ values for the data as reported (Results below and S2 Table) are ~31<60 and ~46<60. If the measurement errors on each date are increased by 25% (rounded upwards), these values become ~46<60 and ~60. If the measurement errors are increased by 50% (rounded upwards), these values become ~61 and ~72. We thus consider the overall Klock model in two versions (a and b): (a) reported values for these data, and (b) an alternative wiggle match with the measurements errors on these data increased by 50%. The first (version (a)) yields A_model_ and A_overall_ values of ~53<60 and 66 with the *Ulmus* sp. RY1001-1002 sample with individual Agreement value ~42<60. The second (version (b)) yields A_model_ and A_overall_ values of ~70 and 79 with the *Ulmus* sp. RY1001-1002 sample with individual Agreement value ~68.

The last ring (including the waney edge) of the above *Ulmus* sp. branch sets a TAQ for House 1 at Klock. House 1 is represented by a temporal sequence of contexts and radiocarbon dates on these. A sample of the charred grass lining from the bottom of the Feature 84 pit (UCIAMS-239714) dates its construction or start of use. The lens of charred maize cobs with kernels attached (kernels: UCIAMS-239713, UCIAMS-218474; cob: UCIAMS-239712) date the primary use of the pit. We employ a weighted average ^14^C age for this apparently single episode/group. Another ^14^C date on a maize sample from Feature 116 pit is likely within this same earlier use horizon (UCIAMS-218476). There are then some ^14^C dates on samples and contexts from likely subsequent House 1 use. A date on maize (UCIAMS-190560) and a date deer bone (UCIAMS-190562) from hearths reflect use during the period of House 1. ISGS-A0326 is on maize from the Feature 50 pit, but, unlike the maize from the Feature 84 pit, this maize deposit was not at the bottom of the pit, and so probably represents use/filling after the pit was no longer serving its original/primary purpose and hence is from the main/later occupation versus initial/early occupation at the site. In addition to House 1 there are several other ^14^C dates on samples from other Klock contexts. One is an outlier that is clearly too old for unknown reasons (laboratory or sample–the measurement was run several decades ago) (AA-7204 on maize) which we exclude; the remaining dated samples belong to somewhere within the period of the Klock occupation (ISGS-A0523 organic residue, UCIAMS-190559 bone, AA-6418 maize, UCIAMS-190561 bone, UCIAMS-218475 maize and UCIAMS-239711 wood charcoal). We consider the date on organic residue as potentially including in-built age and thus apply the OxCal Charcoal outlier model to this date. There is one sub-grouping. Two samples come from Feature 135 pit between Houses 3 and 5: a sample of maize (UCIAMS-218475) and a small ash (*Fraxinus* sp.) twig with 13 tree-rings ending in waney edge/bark. UCIAMS-239711 dates tree-rings RY1009-1013 of this twig, thus we can model the date of the waney edge and use of this twig as 2 years after this date (mid-point treated as RY1011).

The overall Klock site Phase encompasses the House 1 Sequence (before the TAQ from the *Ulmus* branch’s dated waney edge) and the Phase of other dates. Integrated into the overall site model and data, whether we use the quoted ^14^C values for the *Ulmus* sp. samples (a), or those with a 50% measurement error increase (b), makes very little difference other than to the A_model_ values (~53 versus ~68/69) (considering Models 1a v. 1b and 2a v. 2b). The Date query for the overall Klock Phase is almost identical comparing the most likely 68.3% hpd ranges: (a) 1494/5-1521/3 and (b) 1496–1521.

#### Smith-Pagerie

Unburnt bone from Feature 127 (a hearth) in Longhouse (House, H) 4 likely represents post-site-occupation deposition (since we would expect bone to be burnt from use of the hearth during the site occupation period). This sample is thus a TAQ for the site occupation. The latest dated occupation context for the site is the maize from the Feature 80 hearth from Longhouse 2. We regard this as likely a TAQ for the other data from the site in that we would expect periodic cleaning of hearths during a site’s occupation. Four dates are available on samples which can only be placed as from activity within the site Phase represented by Longhouse 1: UCIAMS-190565, UCIAMS-190563 and UCIAMS-190566 on bone from Features 11, 25 and 40 in Longhouse (House) 1, and ISGS-A0528 on organic residue from Feature 15 in Longhouse 1. One date on maize from Feature 60 pit in Longhouse 5, UCIAMS-218493, is an unexplained, much too old outlier (~100% probability if placed in the site Phase with the other data). This could be a sample or laboratory issue but may also reflect earlier activity at the locus. The Feature 60 pit is relatively small and shallow (36” diameter and 24” deep: [76] at p.61) and may reflect limited earlier activity in the first half of the 15^th^ century (the date offers a calibrated calendar age range 1421–1447 at 95.4% probability). We exclude this date from the analysis. We also exclude UCIAMS-218491 from Feature 9 pit in Longhouse 1. We have no information on what material was in fact dated in the case (an error, clearly, which we are not able to retrospectively address). The δ^13^C value, -29.1‰, is also substantially different from all the others from the site, raising suspicions. The age is too recent and is a ~100% probability outlier if modelled in the site Phase. We thus do not include this date in the site model. This leaves the remaining Smith-Pagerie data from Feature 54 pit from Longhouse 1. As discussed above, maize samples from a mid-pit-depth deposit likely date use or residual material during the use period of the pit (UCIAMS-218490, AA-6419 and AA-7405). The context of the charcoal samples (a *Fagus grandifolia* sample to waney edge/bark, a *Betula* sp. sample to waney edge/bark, and a *Fagus grandifolia* sample to waney edge) indicates that the wiggle-matched dates for the waney edge of these samples set a TAQ for the other material in the pit–with these later fills and re-use of the pit quite likely dating after (even well after) the main occupation of the site (and earlier use of the lower part of Feature 54 pit). The three wiggle-matched samples indicate dates for this subsequent use, re-excavation, and re-filling of the pit as represented in the Dark Lens charcoal likely from the mid-16th century to the early 17^th^ century. Material higher in the pit fill may be even more recent (see comment above concerning the report in [114] at p.23).

#### OxCal models

OxCal runfiles for each of the four sites discussed above—Brigg’s Run, Garoga, Klock and Smith-Pagerie—are set out with descriptive notes in S2 File. The model for the analysis of 12 Mohawk River Valley sites, including these four sites, revising and elaborating previous work [65], is set out in S3 File. Note: we use a kIterations value of 3000 in these models, 100x the OxCal default, to ensure the reported models are likely representative. Typical results from several separate runs of each model are shown and reported. We note that very small variations occur between runs of such models. Only models with all values exhibiting individual Convergence values of ≥95 were used.

## Results

**Fig 4:**
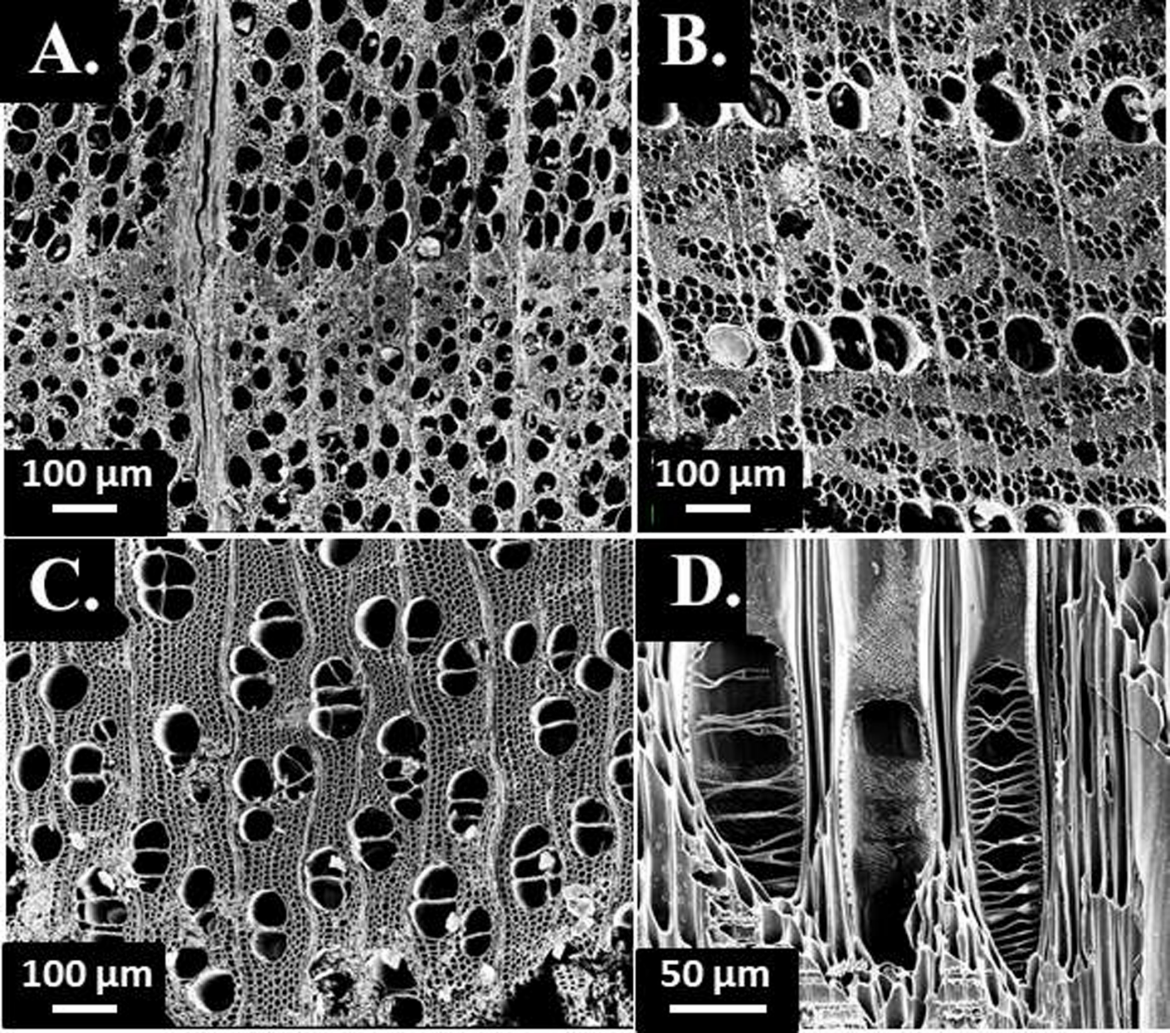
Example FSEM images of wood charcoal taxa sampled for dating, including transverse sections of A. *Fagus grandifolia* from Brigg’s Run (sample A2002.10AC.1.4.7.1), B. *Ulmus* sp. from Garoga (sample A-42354.E.1), C. *Betula* sp. from Smith-Pagerie (sample A-44757.1), and D. radial section of the same *Betula* sp. showing scalariform vessel perforation pits.

We considered each of the Smith-Pagerie, Klock, Garoga and Brigg’s Run sites in separate dating models. Previous assumptions and debates about site relationships among the Smith-Pagerie, Klock and Garoga sites (summarized above in Introduction) were specifically not included in our analysis, in order to obtain a neutral assessment based solely on the data available from each site, considered independently. Using the Bayesian chronological modelling approach as implemented in the OxCal software [58–60, 91, 92], we integrated the calibrated radiocarbon probabilities within each overall site Phase with the constraints available from various contextual and stratigraphic relationships based on archaeological observations from the site and between and from the samples themselves (whether short-lived samples, or samples with in-built age, and samples where tree-ring defined sequences could be used to refine the estimated age of the outermost tree-ring underneath the bark, corresponding to the year of the tree’s felling and human use): see Methods. No assumptions were made other than those related to each site in isolation. Identification and examination of wood-charcoal samples offering short tree-ring sequences (Fig 4) enabled the employment of ^14^C wiggle-matches to constrain and refine dating for each site [67, 68]; in particular, for three sites, tree-ring samples from late, final, or even subsequent use of pits at the sites–after careful review of available documentation (S1 File) – provided probable TAQ constraints. An OxCal Date query was made to estimate the chronological period between the start and end Boundaries for each overall site Phase and is used as an estimate for the site date overall, and an Interval query was made to establish the length of time for each overall site Phase likewise between the start and end Boundaries.

As briefly discussed above, the one key further assumption made concerns the likely overall period of occupation at these sites. According to the specific observations made in early ethnohistoric accounts, as well as assessments made on the basis of the archaeological evidence and resource use estimates, we regard the occupation of each village as being less than ~40 years (e.g. typical maximum range of 20–40 years estimated for the 15^th^ through early 17^th^ centuries [117]), and likely within a total range of 0–30 or 0–40 years, with average site occupation lengths somewhere in the range of ~10–30 or so years. A typical average of (i) ~20 years has been cited ([120] at p.120); or (ii) of ~25 years ([117] at p.49), with larger and more recent sites regarded as having occupation periods more towards the shorter end of these ranges (e.g. [62] at pp.66-67, [48, 68, 117]). We therefore consider three constraints on the duration of each site between the start and end Boundaries via an Interval query with a constraint of either a Log Normal distribution of LnN(ln(20),ln(2)) [68], or a Normal distribution of N(20,10) (derived from [120]) or a Normal distribution of N(25,10) (derived from [117]) (see Fig 2 above). In the case of each site, Model 1 uses the LnN constraint; Model 2 uses the N20,10 constraint; and Model 3 uses the N25,10 constraint. An important consideration is that neither the LnN or N constraint imposes a hard upper limit (in contrast with a uniform prior assumption), such that the data from each of the sites overwhelms the prior if the assumption used above is in fact not appropriate, and so indicate a problem. We find only very modest differences across all three of these models, and the Posterior probabilities correspond well with the Prior probabilities in each case indicating that data, assumptions and model are reasonably well aligned (S2 Table).

**Fig 5:**
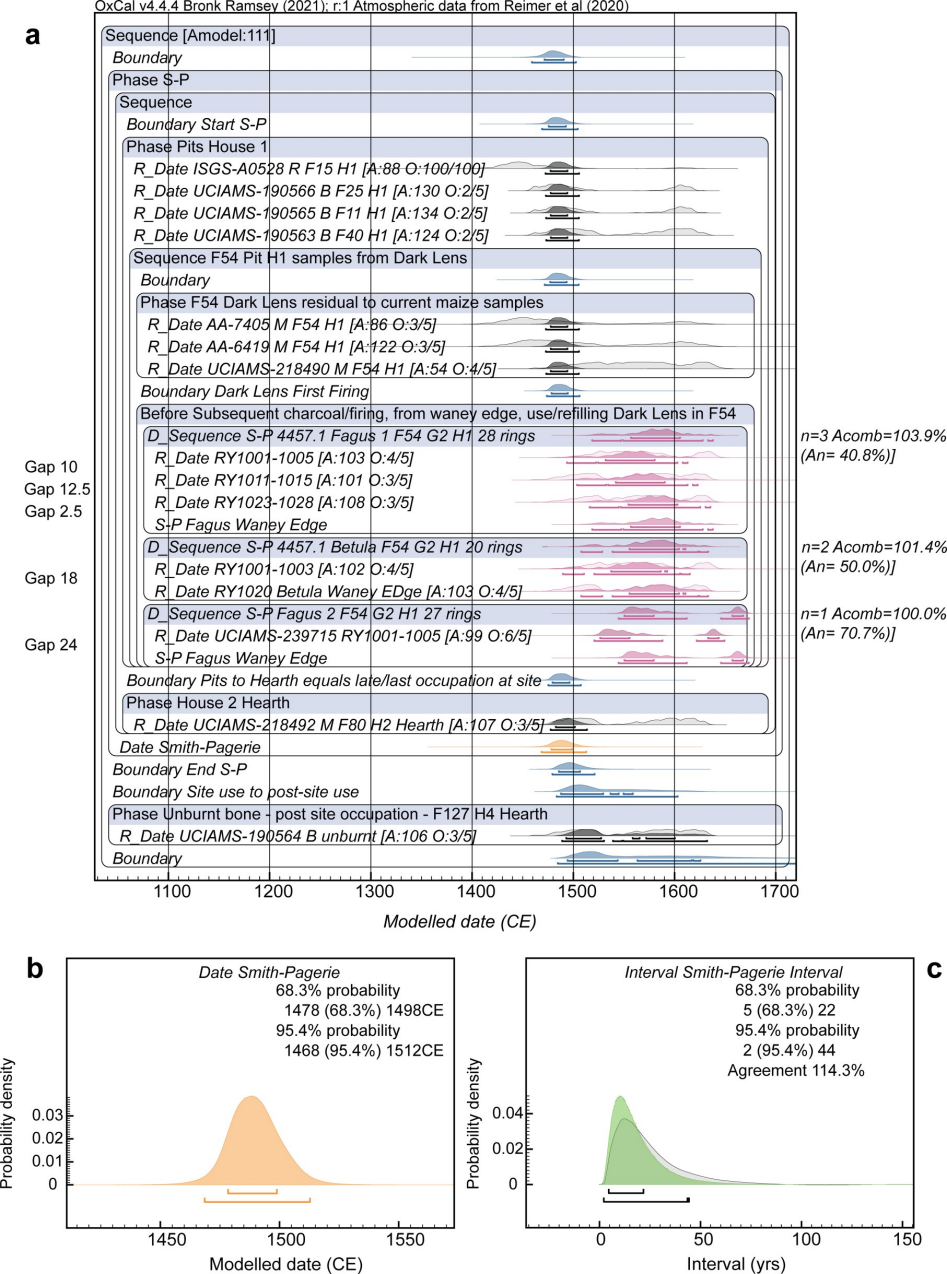
Dating model and results for Smith-Pagerie (S-P) Model 1. **a.** Site dating model with the OxCal key words and boxes describing the model exactly. Light-shaded distributions show the non-modelled calibrated calendar probabilities; the dark shaded distributions show the modelled probabilities applying the model. The lines under each distribution show the modelled 68.3% and 95.4% highest posterior density (hpd) ranges. The individual OxCal Agreement values (A) and the Outlier (O) values (Posterior/Prior) are shown (note in the case of the Charcoal Outlier model these are always 100/100). **b.** Detail from a. showing the Date query result for the overall Smith-Pagerie Phase. **c.** Interval query result, applying the LnN(ln(20),ln(2)) prior, showing the probability for the period of time between the start and end Boundaries for the site Phase in a. versus the prior (there is good agreement). Data from OxCal [91, 92, 115] version 4.4.4 of 2021 using IntCal20 [111] with curve resolution set at 1-calendar year.

**Fig 6:**
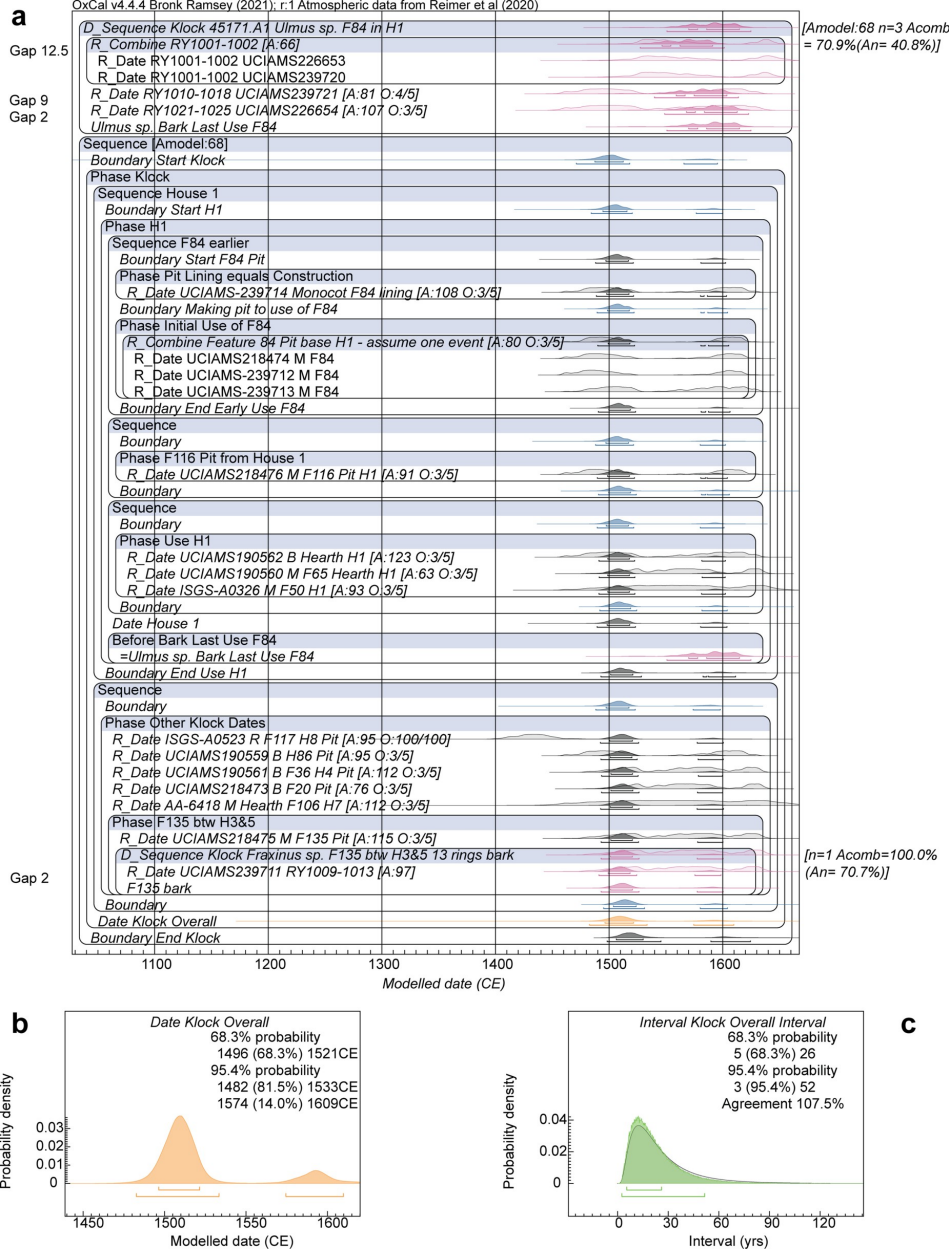
Dating model and results for Klock Model 1b. **a.** Site dating model. **b.** Detail of the Date query estimate for the overall Klock site Phase. **c.** Interval query result, applying the LnN(ln(20),ln(2)) prior, showing the probability for the period of time between the start and end Boundaries for the site Phase in a. versus the prior. For description of the figure, see the caption to Fig 5.

**Fig 7:**
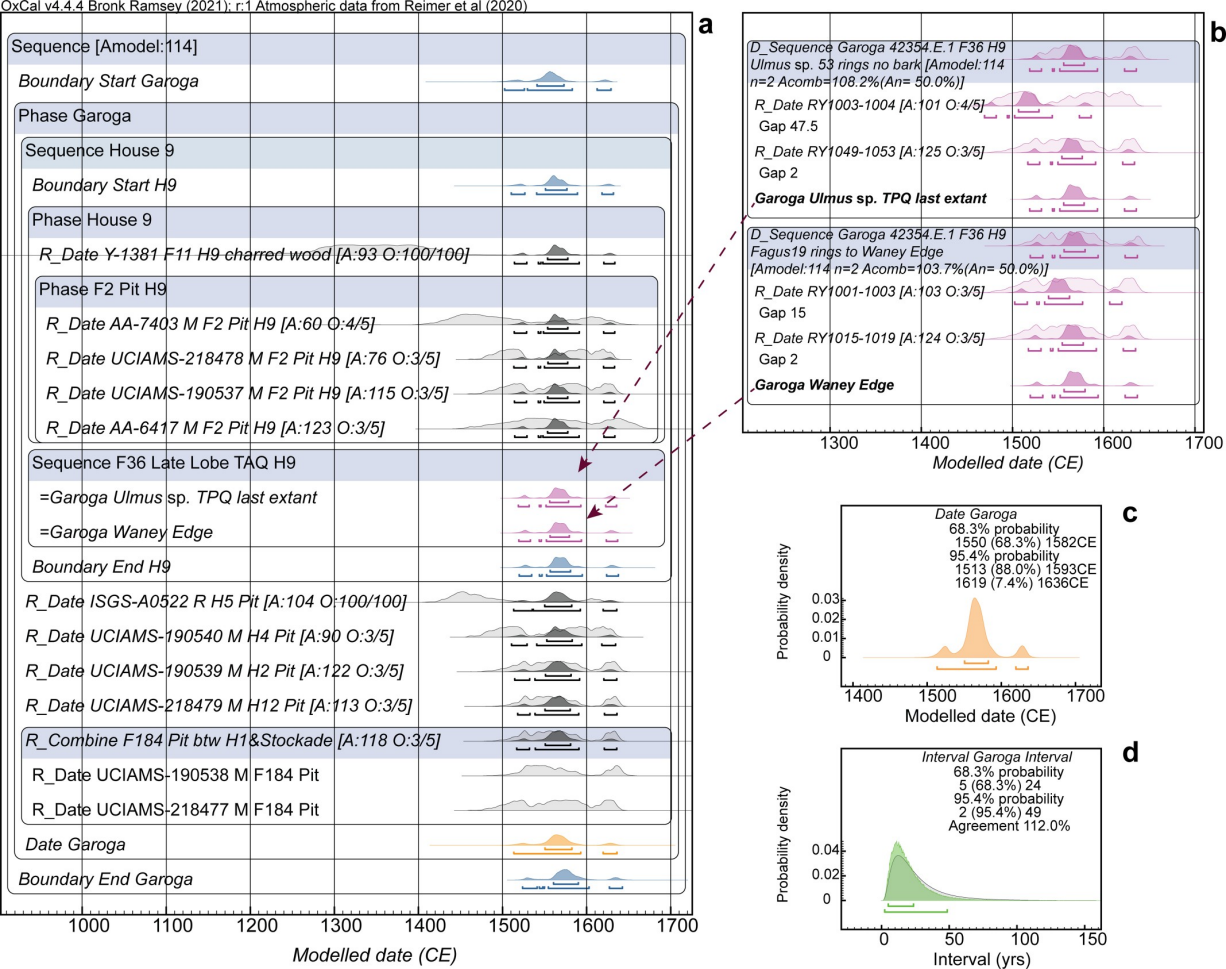
Dating model and results for Garoga Model 1. **a.** The site dating model. **b.** Two short wiggle-matches run on the late (end of use) lobe from the Feature 36 pit in Longhouse 9 and cross-referenced into the model in a. **c.** Detail of the Date query for the overall Garoga site Phase from a. **d.** Interval query result, applying the LnN(ln(20),ln(2)) prior, showing the probability for the period of time between the start and end Boundaries for the site Phase in a. versus the prior. For description of the figure, see the caption to Fig 5.

**Fig 8:**
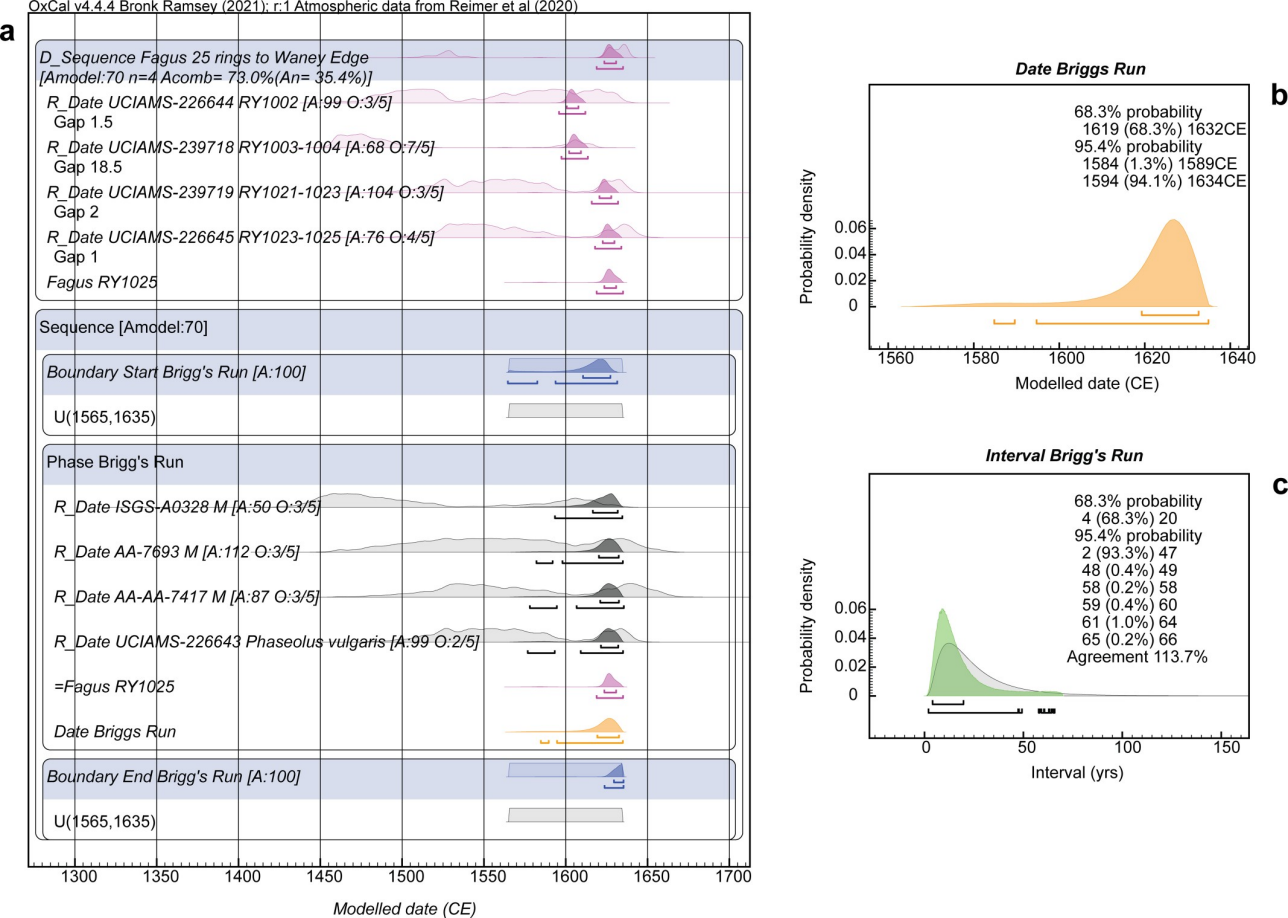
Dating model and results for the Brigg’s Run site Model 1. **a.** The site dating model. The site Phase is constrained (very conservatively) as starting and ending with uniform probability between 1565 and 1635 (see Methods). A short wiggle-match is cross-referenced as belonging to somewhere within the site Phase. No contextual/site information is available on the Brigg’s Run material (see Methods). **b.** Detail of the Date query estimate for the overall Brigg’s Run site Phase. **c.** Interval query result, applying the LnN(ln(20),ln(2)) prior, showing the probability for the period of time between the start and end Boundaries for the site Phase in a. versus the prior. For description of the figure, see the caption to Fig 5.

Figs 5–8 show the site dating models and the results for each of the Smith-Pagerie, Klock, Garoga and Brigg’s Run sites from the site Model 1 cases (LnN constraint). Table 1 lists a summary of the results from both Model 1 (LnN constraint) and Model 2 (N20,10 constraint) for each of the four sites and two alternatives for the tree-ring wiggle-match from the Klock site. The respective results are very similar (S2 Table shows comparisons of Models 1, 2 and 3 and some additional details). Thus, the particular choice of (broadly similar) constraint Prior is not decisive [68]. In each case, except one, the models offer satisfactory OxCal A_model_ and A_overall_ values (>60) and the likely date range of each site is evident with minimal realistic ambiguity. There is no ambiguity in any of the most likely 68.3% hpd ranges. The one exception regarding the A_model_ values is Klock Model 1a, Model 2a and Model 3a. As discussed in Methods (above), the wiggle-match of the dated tree-ring samples from the *Ulmus* sp. branch from Feature 84 is less than satisfactory given the available data and the IntCal20 smoothed calibration curve (Fig 3A). Either the laboratory data are overly precise (or slightly inaccurate), or it may well be that underlying greater high-frequency (annual-scale) variation in atmospheric ^14^C and calibration values is reflected in our data, as suggested by comparison with the raw ^14^C data from which IntCal20 is derived and especially the large recent ETH dataset [108] (Fig 3B). Klock Models 1b, 2b and 3b approximately allow for greater variation by increasing the measurement errors on each sample in this wiggle-match by 50%–this solves the OxCal Agreement issue but makes no substantive change to the likely date placement for the site.

**Table 1:** Summary of typical results for the site dating for the models for Smith-Pagerie, Klock, Garoga and Brigg’s Run from the respective site Models 1 (Figs 5–8) and Models 2 (and the a and b variations for Klock) (see Methods).

	A_m_	68.3% hpd	95.4% hpd		A_m_	68.3% hpd	95.4% hpd
**Smith-Pagerie Model 1**	111			**Smith-Pagerie Model 2**	108		
Boundary Start		1475–1492	1469–1504	Boundary Start		1475–1492	1469–1505
Date		1478–1498	1468–1512	Date		1478–1499	1469–1511
Boundary End		1485–1506	1479–1521	Boundary End		1486–1507	1479–1519
*Interval*		*5–22*	*2–44*	*Interval*		*7–27*	*0–35*
**Klock Model 1a**	53			**Klock Model 2a**	53		
Boundary Start		1485–1513	1472–1517 (77.5) 1563–1595 (18.0)	Boundary Start		1487–1510	1478–1517 (80.5) 1569–1593 (15.0)
Date		1494–1523	1483–1532 (77.2) 1573–1611 (18.2)	Date		1495–1521	1486–1530 (80.4) 1576–1606 (15.1)
Boundary End		1505–1531	1498–1543 (77.4) 1587–1624 (17.9) 1626–1627 (0.2)	Boundary End		1507–1529	1498–1538 (80.8) 1590–1615 (14.7)
*Interval*		*5–26*	*3–53*	*Interval*		*10–30*	*1–38*
**Klock Model 1b**	68			**Klock Model 2b**	69		
Boundary Start		1487–1512	1470–1517 (82.4) 1565–1594 (13.1)	Boundary Start		1487–1510	1478–1517 (82.0) 1578–1599 (13.5)
Date		1496–1521	1482–1533 (82.0) 1574–1610 (13.4)	Date		1496–1521	1485–1530 (81.8) 1576–1606 (13.7)
Boundary End		1503–1523	1495–1531 (82.7) 1580–1604 (12.7)	Boundary End		1507–1529	1498–1539 (82.2) 1591–1615 (13.2)
*Interval*		*5–27*	*2–53*	*Interval*		*10–30*	*2–39*
**Garoga Model 1**	114			**Garoga Model 2**	111		
Boundary Start		1541–1573	1502–1626 (10.4) 1529–1583 (77.5) 1612–1629 (7.6)	Boundary Start		1543–1571	1503–1524 (9.1) 1533–1581 (79.4) 1612–1628 (6.9)
Date		1550–1582	1513–1593 (88.0) 1619–1636 (7.4)	Date		1551–1581	1512–1534 (8.3) 1539–1593 (80.3) 1619–1635 (6.8)
Boundary End		1560–1590	1523–1541 (7.4) 1543–1547 (0.8) 1549–1549 (0.1) 1554–1602 (79.1) 1627–1642 (8.1)	Boundary End		1561–1588	1524–1543 (8.3) 1555–1600 (79.8) 1628–1643 (7.3)
*Interval*		*5–24*	*2–50*	*Interval*		*8–27*	*1–37*
**Brigg’s Run Model 1**	70			**Brigg’s Run Model 2**	67		
Boundary Start		1610–1627	1564–1582 (8.7) 1593–1631 (86.7)	Boundary Start		1608–1627	1570–1571 (0.1) 1588–1634 (95.3)
Date		1619–1632	1585–1588 (0.9) 1593–1634 (94.6)	Date		1619–1632	1599–1635
Boundary End		1629–1635	1623–1635	Boundary End		1629–1635	1622–1635
*Interval*		*4–20*	*2–46 (93*.*3) 47–48 (0*.*4) 59–65 (1*.*8)*	*Interval*		*4–22*	*0–33*

For further details and Model 3 results, see S2 Table. Note that every run of such models can produce very slightly results. Typical variations are in the ~0–2 year range; most variations occur in the less likely parts of the 95.4% hpd ranges. A_m_ = A_model_.

**Fig 9:**
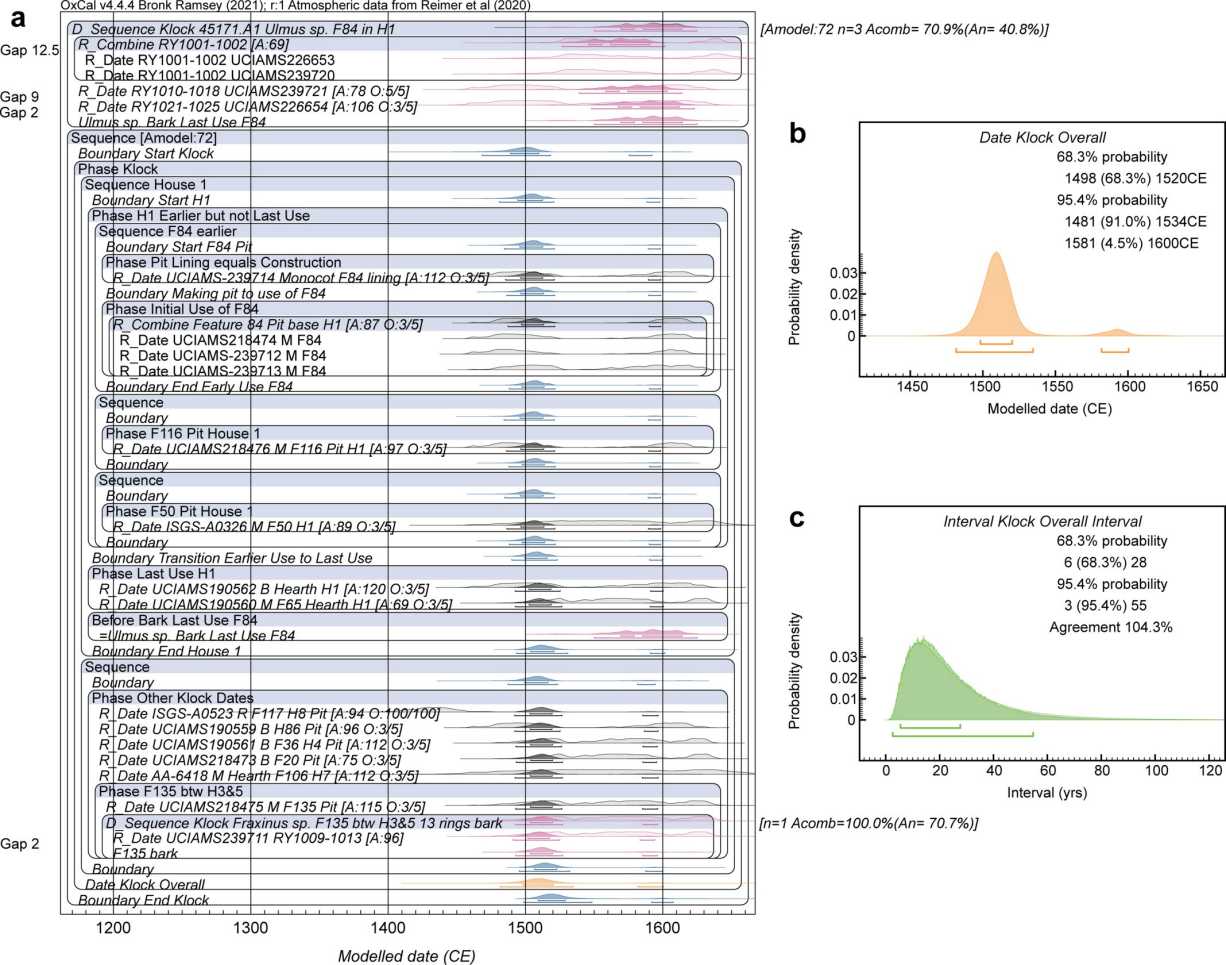
Alternative dating model and results for Klock Model 1b if revised to place the dates from hearth contexts as the latest use episode for House 1. **a.** Revised site dating model. **b.** Detail of the Date query estimate for the overall Klock site Phase from the revised model. **c.** Interval query result, applying the LnN(ln(20),ln(2)) prior, showing the probability for the period of time between the start and end Boundaries for the site Phase in a. versus the prior from the revised model. For description of the figure, see the caption to Fig 5. The alternative OxCal runfile code is listed in S2 File.

In the case of Klock, one other possible variation in assumptions seems worth consideration. It might be argued as plausible that the samples found from (we may assume) the last use(s) of the hearth feature in House 1 (CIAMS-190562 on bone and UCIAMS-190560 on maize) reflect the last use of House 1 and so set a TAQ for the earlier material from the house and pits therein. If we revise Klock Model 1b to reflect this additional assumption, placing the dates on material from the hearth as the latest in the house Sequence, it only further solidifies the preferred date range for the site identified in each of Models 1–3: see Fig 9. The overall Klock site Phase Date estimate in this model at 68.3% hpd is 1498–1520 (or 1481–1534 (91.0%) and 1581–1600 (4.5%) at 95.4% hpd).

A very few larger outliers were identified and excluded in the final site models employed (see Methods, S2 File). As discussed in Methods, some of these outliers may be laboratory outliers or problematic samples (especially UCIAMS-218491). In a few instances the data are from recent AMS ^14^C measurements and still yielded substantially older ages. In such circumstances, where no in-built age can be explained in terms of the sample (e.g., random wood charcoal or organic residue from a ceramic vessel), then it is possible that there was previous human activity at the locus—indeed, multiple uses of suitable, even strategic, loci through time have been observed or suggested in other work in the region (e.g. [39, 69]). It is notable that the modelled Intervals show good agreement between the calculated Posterior versus the Prior assumptions in each case–suggesting that the data do support and conform with the ethnographic and archaeological assessments of typical village occupation length. The likely date placements for the Smith-Pagerie, Klock and Garoga sites contradict some previously hypothesized site relationships and dates (as also does recent social network analysis: [80]). For example, the assessment of a site-community occupation sequence (oldest to youngest) of Garoga then Klock and then Smith-Pagerie by Funk and Kuhn ([76] at p.142) is challenged. The Bayesian chronological models for each of these sites in Figs 5–7 offer the basis for a well-resolved, independent, temporal framework.

**Fig 10:**
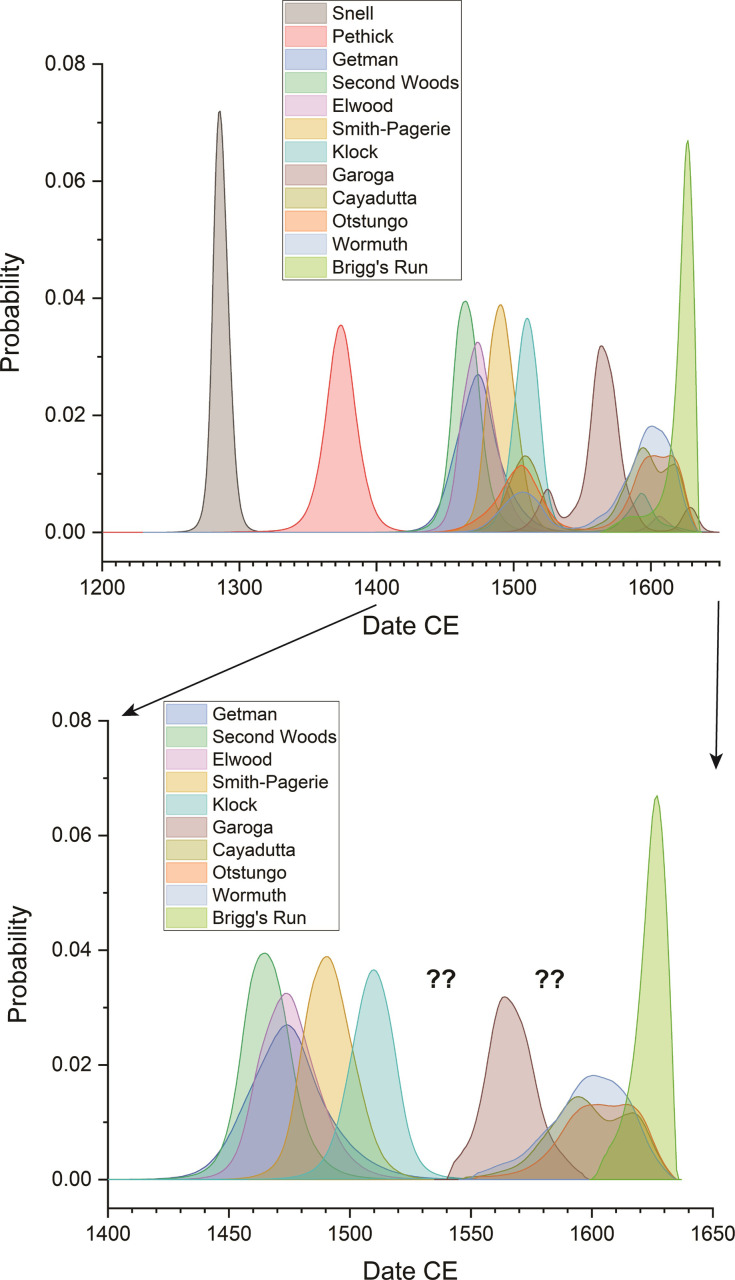
The modelled calendar dating probabilities for the Mohawk Valley sites in this study and adding eight other sites from previous work [65]. **a.** The dating probabilities for the 12 sites using the site models for Smith-Pagerie, Klock, Garoga and Brigg’s Run from Figs 5–8 and those for the other 8 sites from a re-run of the data in [65] but applying now the LnN(ln(20),ln(2)) prior to the Interval query in each case. **b.** Detail of the mid-15^th^ through early 17^th^ century period and removing instances of alternative less likely probability ranges in **a**. The ?? marks indicate two periods, based on less available evidence, where we may expect one or more other sites to date (before/after and contemporary with Garoga)–several other sites are known from the overall Mohawk Valley region that have so far not been part of recent dating efforts (see the set of sites in [80]).

We further considered the four sites dated in Figs 5–8 (Model 1 for each site) in terms of eight other Mohawk Valley sites discussed in recent work: the Snell, Pethick, Getman, Second Woods, Elwood, Cayadutta, Otstungo and Wormuth sites [65]. We did not include the Palatine Bridge site as this site has only two ^14^C dates and we did not run additional samples in this study. We re-ran the dating models available for these other eight sites from previous work ([65]–using the Model 2 version from that study) but incorporating an Interval query constraint of the form LnN(ln(20),ln(2)), as in the Model 1 constraints for Smith-Pagerie, Klock, Garoga and Brigg’s Run in the present study (Figs 5–8). We did not place the site set within an overall Phase but treated each site Phase as entirely independent. The site Date query results for all 12 sites are shown in Fig 10A and Table 2. In some cases observing the calendar dating probabilities, there is a clear most likely modelled age range (majority of the most likely probability) and then an alternative less likely range (with a minority of the most likely probability). In Fig 10B we show a detail of the 10 sites lying in the mid-15^th^ to early 17^th^ centuries and (subjectively and arbitrarily) remove the less likely alternative probability regions. This reveals a likely approximate time-frame for this set of 10 sites. We compare the date ranges achieved against one set of previous date estimates for 11 of these 12 sites in Table 2 in order to illustrate the instances of marked changes in temporal placements.

**Table 2:** Date query estimates for the overall site phases (period between the start and end Boundaries for the overall site phase) for the 12 Mohawk Valley sites shown in Fig 10A.

	68.3% hpd (i)	95.4% hpd	Dates in Snow [39] (ii)	Overlap (i) v. (ii)
**Snell**	1280–1292	1274–1299	1275–1350	Yes
**Pethick**	1362–1386	1346–1401	N/A	N/A
**Getman**	1456–1488	1441–1511	1400–1450	**No**
**Second Woods**	1455–1475	1443–1490	1450–1500	Yes
**Elwood**	1460–1486	1448–1506 (92.7) 1600–1612 (2.7)	1450–1500	Yes
**Smith-Pagerie**	1479–1500	1470–1513	1560–1580	**No**
**Klock**	1500–1521	1486–1532 (88.8) 1577–1599 (6.6)	1540–1565	**No**
**Garoga**	1550–1581	1512–1592 (91.0) 1621–1634 (4.4)	1525–1545	**No**
**Cayadutta**	1498–1518 (22.6) 1583–1622 (45.7)	1482–1529 (36.3) 1565–1631 (59.1)	1525–1545	**No**
**Otstungo**	1493–1516 (23.6) 1586–1624 (44.6)	1467–1534 (41.0) 1570–1631 (54.5)	1450–1525	**No**
**Wormuth**	1502–1510 (5.6) 1576–1622 (62.7)	1481–1527 (22.3) 1556–1630 (73.1)	1450–1525	**No**
**Brigg’s Run**	1619–1632	1584–1589 (0.9) 1593–1634 (94.6)	1614–1626	Yes

Note the ranges or probabilities for Smith-Pagerie, Klock, Garoga and Brigg’s Run vary very slightly from the Table 1 Model 1 results–and each run of such models sees very small variations. The likely date region, used to discriminate where ambiguity, for Fig 10B is the region around the underlined probabilities. The previous dates suggested for 11 of these sites by Snow [39] are also listed to indicate the changes (or not) involved with the current results.

For the first time, there is now a relatively clear and resolved (unambiguous) chronology for these Mohawk Valley sites. In this regard our findings here confirm, but importantly revise and refine, initial ^14^C based work [65]. The calendar age ranges we achieve for each of these village sites start to be sufficiently resolved as to be representative and informative of both the likely relatively short life-cycles of these settlements, and to allow us to begin to elucidate a human-scale, historical-level, approach to the investigation of the constituent social groups, their connectivities, and the complex set of entangled relationships involving place, climate-environment, agents, actions, events and outcomes from village to regional spatial scales ([50, 62, 80, 94, 121–123]). We note that there are additional sites in the Mohawk Valley region from this general era (e.g. [80]). It is likely some of these date within the same time range and, based on a current absence of evidence, we might especially suspect that some of these sites, at least, likely date in the periods before and after Garoga, since we currently have less evidence for dated sites at these times (marked by the **??** in Fig 10B), especially ~1520–1550, and perhaps also ~1570–1590. Our findings thus pinpoint targets for future research in the 16^th^ century, especially, and highlight how much we are yet to learn and to delineate if we are to narrate a more comprehensive history.

## Discussion

The period around the initial European presence and then invasion and settlement of northeastern North America in the 15^th^ through early 17^th^ centuries has conventionally been dated and described in terms of European history and the absence, presence and types of European trade goods. The first part of the title of a 1995 article perfectly captures the ingrained thought processes involved in most work until recently: “Untanglers of matters temporal and cultural: glass beads and the early contact period…” [120]. This predominance of Eurocentric thinking and approach is understandable. Previous limited engagement with, and direct knowledge of, the Indigenous viewpoint [6, 15, 17–20], and a focus therefore on the material culture record [16], rendered alternatives challenging until a refined independent dating approach became available. As noted in the Introduction and in other recent studies (e.g. [62, 63, 65]), until very recently, the standard archaeological dating technology, radiocarbon dating, was long regarded as less than helpful in this period due to the wide calendar age ranges obtained for individual age measurements because of the shape of the radiocarbon calibration curve (a reversal-plateau in the 16^th^ century). Thus, despite pioneering efforts to obtain large sets of radiocarbon dates, such as by [39] for the Mohawk Valley, the available material culture, and especially the European trade goods therein, continued to dominate assessments and thought frameworks into the beginning of the 21^st^ century. It is only very recently that the combination of modern AMS ^14^C dating, allowing dates on small samples and with good measurement precision, allied with Bayesian chronological methods incorporating prior knowledge informing and constraining temporal relationships between variously samples, different contexts at a site, and overall occupation duration, have enabled temporal resolution at the scale of Indigenous village life-cycles and the associated human lived experience and history (e.g. [63, 64, 68]). In particular, as demonstrated in our analyses of the Smith-Pagerie, Klock, Garoga and Brigg’s Run sites, we base our analysis on the single, individual, sites and their contextual and secure historical associations considered in isolation, without imposing either any assumptions concerning site relocation sequences or by achieving an order within a sequence of sites, when several sites may in fact have been partly or even wholly contemporaneous (a major improvement over a previous initial analysis [65]). This independence of analysis is essential–avoiding past archaeological assumptions of site relationships as much as possible–since recent social network analysis has raised questions over previous, relatively loosely derived, assumptions in this regard in several cases [50, 80].

As in other recent Bayesian chronological modelling work in northeastern North America employing substantial sets of AMS ^14^C dates, our findings presented in this study challenge, and in some cases contradict, previous conventional interpretations of site dates and associated regional chronologies based on artefact types and assemblages. At the specific level of the iconic set of three villages formally considered to belong to one community and successive occupations, ordered as Garoga, Klock, and Smith-Pagerie ([76] at p.142), it was almost inevitable that our independent dating using radiocarbon allied with tree-ring sequenced information identifies that the sites not only likely lie in the exact reverse temporal order, but also that their temporal placements are consistent with findings from social network analysis indicating that it is unlikely these villages represent the same community [80]. Past assumptions need to be rethought entirely.

Overall, it is evident through a comparison of the date estimates obtained in this study, versus those from a standard assessment from a quarter of a century ago [39] (Table 2), that the main area of change and revision is in the period related to initial European associations and presence in northeastern North America, that is: the later 15^th^ through 16^th^ centuries. Earlier periods were genuinely prehistoric, and the dates were based on available radiocarbon dates since that technology became available. Later periods, from the early-mid-17^th^ century onwards, fall into a much better documented era (e.g. [11, 21, 22]). However, in between, if we remove the cultural lens that assumes we should try to best employ European associations and their type, or their absence, as the critical temporal determinant, and instead employ direct, independent, absolute dating based on ^14^C wiggle-matching and AMS radiocarbon dates incorporated into Bayesian chronological models, we find in a number of cases a substantially different timeframe. In turn, this provides the basis to a very different basic history of lives and use of places in northeastern North America in the period from the late 15^th^ through 16^th^ centuries. This period is revealed as a long century of dramatic changes in many aspects of society and cultural landscapes across the region–relating to processes including settlement aggregation, violent conflict, population movements and the formation of political confederacies–that only now we can start to define and tease apart given accurate and reasonably precise resolution for settlement and community loci in both time and space [50, 62, 63, 64, 80]. An accurate and refined timescale offers a belated opportunity to reconsider and re-investigate the North American world that Europeans invaded from a more neutral viewpoint with the material culture considered in terms of its own time and place, and not through an imposed European-derived narrative. Such an accurate and independent timescale also provides the appropriate framework into which to include and employ oral history.

The relatively refined and high-resolution definition of time for the sites focused on in this study highlight that the presence or absence of initial and limited quantities of European trade goods often cannot be used satisfactorily to define chronology [62–65, 69]. Where there are extensive, replicated, and plural connections with European goods and typologies, then the radiocarbon-based analysis offers a date range that is compatible with the historical information. In our present study this describes the situation with the Brigg’s Run site, and in previous recent work this describes the findings for the Warminster site in Ontario, likely directly linked with Samuel de Champlain who spent the winter of 1615–1616 at the site [63, 64]. However, in other cases where no such plural, replicated, and multiple connections exist, it is clear that selective, or historically determinist assumptions, based on limited occurrences and their types, or absences, can be entirely misleading. Contrary previous assumptions, some sites (or at least individuals or groupings at some sites) in the Mohawk Valley acquired European goods (especially metals) early in the history of European involvement in North America.

Detailed contextual study and additional radiocarbon dates confirm previous indications that the artefacts determined to be of European iron and copper, the latter by pXRF analysis, at Smith-Pagerie are presently the earliest well-dated examples of European metal circulation in Northern Iroquoia [65] (Fig 5). The early date, likely in the last couple of decades of the 15^th^ century, highlights how very little we know about European contacts and involvement with northeastern North America outside the few well-known episodes of the long-told standard narrative, such as Cartier landing in the St. Lawrence in 1534 and the establishment of Fort Nassau in New York from 1614. Earlier Norse contact and settlement is attested around 1000, and some other links between then and the 15^th^ century are possible or hinted at [10, 124, 125]. History recounts Cabot reaching Newfoundland in 1497 [126], and a subsequent fishing boom, but it is evident that there must have been many, many other–but not necessarily official (royal sponsorships and similar) nor well-documented–interactions with the Indigenous populations of northeastern North America also in the period at least from the later 15^th^ through 16^th^ centuries [9, 10, 126–129], especially via fishing and whaling crews [127, 129], before the establishment of sustained European settlements in northeastern North America from Arcadia to Fort Nassau to Jamestown in the first years of the 17^th^ century (1604–1614). The recent re-discovery of William Weston and his voyage from Bristol to North America ~1499, is just one example of a rich, but largely hidden, history [130]. In correcting the past European derived timescale for Northeast North America in the later 15^th^ through 16^th^ centuries from direct archaeological evidence there is thus an irony. In doing this we also throw additional important light, in reverse, via the independent dating of early European materials at Indigenous sites in North America—like Smith-Pagerie, Klock and Garoga [65]—on an important, world-changing, but largely forgotten and hidden period of early modern Atlantic connectivity from which only a few famous names have retained their currency into our modern histories. There remains much still to learn about the beginnings of the early modern globalized world.

## Supporting information

S1 Table Radiocarbon dates from the Briggs’ Run, Garoga, Klock, and Smith-Pagerie sites used in the site models in S2 Table. Note: samples of *Zea Mays* ssp. *mays* are listed as “maize”. Similarly, we refer to white-tailed deer rather than *Odocoileus virginianus*. RY refers to Relative Year for tree-ring samples–this means the annual growth increments (tree-rings) within the sample. Note: the four *Ulmus* sp. samples listed as NYSM #45171.A1 are from the same small diameter branch sample. Two samples were mistakenly listed as *Fagus* sp. in the UCIAMS laboratory report but the samples sent (as part of two different sets of samples) were all *Ulmus* sp. and from the same branch. Wood charcoal is identified where it was part of the present study–samples and dates from previous work (e.g. the sample for date Y-1381) could not be identified if the relevant reports/publications did not provide this information. Missing δ^13^C values reflect our inability to find these in some older reports/publications. The δ^13^C values noted with an * were stated as estimated (versus measured) by the radiocarbon laboratory. The value replaced by ** was a case where the sample was too small for a separate IRMS assay; following normal UCIAMS laboratory protocols the δ^13^C was measured in the AMS for isotopic correction of the ^14^C age but this AMS-derived δ^13^C is not reported. Measurements in original reports converted to cm where previously in inches. Note: the reported date on a sample from Brigg’s Run, AA-8369 ([39] at p.259) that is considerably older than the other dating evidence from the site (material culture or radiocarbon) is not used and, assuming this sample is in fact from the exact same site, then, as proposed by [39], “it appears that once again a seventeenth-century Mohawk village was built on top of an earlier component, a characteristic of several late Mohawk village sites” [39, 65, 72, 76, 131, 132].
(DOCX)

S2 Table Comparison of summary of results from Model 1, LnN(ln(20),ln(2)), Interval query constraint, Model 2, N(20,10), Interval query constraint, and Model 3, N(25,10), Interval query constraint for the Smith-Pagerie, Klock, Garoga and Brigg’s Run sites and queries related to two specific features at these sites (House, H, 9 at Garoga and House, H, 1 at Klock). (DOCX)

S1 File Field drawings and transcribed notes for features described in the article text. Field drawings have been cropped to remove individual’s names. Note: in S1 File the measurements are given in feet (‘) and inches (”) as listed in the original site documentation and drawings (some reproduced below).
(DOCX)

S2 File OxCal runfiles for the models in Figs 5–8. Note: some of the labels/descriptions are edited for display in Figs 5–8. Note: *Zea mays* (maize) samples are identified as M, *Odocoileus virginianus* (white-tailed deer) samples are identified as B, and organic residue samples are identified as R. For details, see S1 Table. Note, for reasons of display space, samples of *Fagus grandifolia* (beech) are listed just as “Fagus”.
(DOCX)

S3 File OxCal runfile for the model in Fig 10. This model employs the site models (Model 1) for Smith-Pagerie, Klock (Model 1b), Garoga and Brigg’s Run from those listed in S2 File (and as used for Figs 5–8). The data and models for the other eight sites are taken from the Model 2 versions in [65] with the change that the Interval statement now includes the LnN(ln(20),ln(2)) constraint (and we now employ IntCal20 [111]). Otherwise, the data and models for each site are the same as previously published (thus these sites have a uniform probability constraint placed on the start and end Boundaries between 1150 and 1635 CE as explained in [65]. Note: *Zea mays* (maize) samples are identified as M, *Odocoileus virginianus* (white-tailed deer) samples are identified as B, and organic residue samples are identified as R. For details, see S1 Table and [65]. Note, for reasons of display space, samples of *Fagus grandifolia* (beech) are listed just as “Fagus”.
(DOCX)
