# Supplementary material for: Resolving Indigenous village occupations and social history across the long century of European permanent settlement in Northeastern North America: The Mohawk River Valley ~1450-1635 CE
Source: PLoS One. 2021 Oct 15;16(10):e0258555. doi: 10.1371/journal.pone.0258555 (PMC8519479; doi:10.1371/journal.pone.0258555)
Supplement: S1 Table — Note: samples of Zea Mays ssp. mays are listed as “maize”. Similarly, we refer to white-tailed deer rather than Odocoileus virginianus. RY refers to Relative Year for tree-ring samples–this means the annual growth increments (tree-rings) within the sample. Note: the four Ulmus sp. samples listed as NYSM #45171.A1 are from the same small diameter branch sample. Two samples were mistakenly listed as Fagus sp. in the UCIAMS laboratory report but the samples sent (as part of two different sets of samples) were all Ulmus sp. and from the same branch. Wood charcoal is identified where it was part of the present study–samples and dates from previous work (e.g. the sample for date Y-1381) could not be identified if the relevant reports/publications did not provide this information. Missing δ13C values reflect our inability to find these in some older reports/publications. The δ13C values noted with an * were stated as estimated (versus measured) by the radiocarbon laboratory. The value replaced by ** was a case where the sample was too small for a separate IRMS assay; following normal UCIAMS laboratory protocols the δ13C was measured in the AMS for isotopic correction of the 14C age but this AMS-derived δ13C is not reported. Measurements in original reports converted to cm where previously in inches. Note: the reported date on a sample from Brigg’s Run, AA-8369 ([39] at p.259) that is considerably older than the other dating evidence from the site (material culture or radiocarbon) is not used and, assuming this sample is in fact from the exact same site, then, as proposed by [39], “it appears that once again a seventeenth-century Mohawk village was built on top of an earlier component, a characteristic of several late Mohawk village sites” [39, 65, 72, 76, 131, 132]. (DOCX) [file pone.0258555.s001.docx]

**S1 Table. Radiocarbon dates from the Briggs’ Run, Garoga, Klock, and Smith-Pagerie sites used in the site models in Table S2.** Note: samples of *Zea Mays* are listed as “maize”. Similarly, we refer to white-tailed deer rather than *Odocoileus virginianus*. RY refers to Relative Year for tree-ring samples – this means the annual growth increments (tree-rings) within the sample. Note: the four *Ulmus* sp. samples listed as NYSM #45171.A1 are from the same small diameter branch sample. Two samples were mistakenly listed as *Fagus* sp. in the UCIAMS laboratory report but the samples sent (as part of two different sets of samples) were all *Ulmus* sp. and from the same branch. Wood charcoal is identified where it was part of the present study – samples and dates from previous work (e.g. the sample for date Y-1381) could not be identified if the relevant reports/publications did not provide this information. Missing δ^13^C values reflect our inability to find these in some older reports/publications. The δ^13^C values noted with an * were stated as estimated (versus measured) by the radiocarbon laboratory. The value replaced by ** was a case where the samples was too small for a separate IRMS assay; following normal UCIAMS laboratory protocols the δ^13^C was measured in the AMS for isotopic correction of the ^14^C age but this AMS-derived δ^13^C is not reported. Measurements in original reports converted to cm where previously in inches. Note: the reported date on a sample from Brigg’s Run, AA-8369 ([39] at p.259) that is considerably older than the other dating evidence from the site (material culture or radiocarbon) is not used and, assuming this sample is in fact from the exact same site, then, as proposed by [39], “it appears that once again a seventeenth-century Mohawk village was built on top of an earlier component, a characteristic of several late Mohawk village sites”.

| **Site** | **NYSM #** | **Material** | **Context** | | **Lab #** | **δ^13^C‰** | **^14^C age yrs BP** | **Source** |
| --- | --- | --- | --- | --- | --- | --- | --- | --- |
| Garoga | A-42332 | wood charcoal | Feature 11, cylindrical pit feature measuring 94 cm in diameter and 147 cm deep in the midline of Longhouse 1 ([76] at p.98). From ‘refuse’ layer in lower third of pit fill. | | Y-1381 | N/A | 640±100 | [72] at p.330 |
| Garoga | A-42355 | maize kernel | Feature 37, cylindrical pit measuring 94 cm in diameter and 160 cm deep within Longhouse 9 ([76] at p.99). No indication of where the specimen was collected in field notes. | | AA-8370 | -10* | 585±40 | [39] at p.164 |
| Garoga | A-42235F | maize kernel | Feature 2, pit measuring 106 x 122 cm in plan within Longhouse 9 ([76] at p.98). Field notes indicate a concentration of maize but do not indicate where in the fill it was located. | | AA-7695 | -11* | 431±49 | [39] at p.164 |
| Garoga | A-42826-2 | charred cooking residue adhering to pottery sherd interior | Feature 208, a pit measuring 61 cm deep within Longhouse 5 ([76] at p.107). No notes were found for this feature. | | ISGS-A0522 | -20.8 | 425±40 | [131] |
| Garoga | A-42235F | maize kernel | Feature 2, pit measuring 106 x 122 cm in plan within Longhouse 9 ([76] at p.98). Field notes indicate a concentration of maize but do not indicate where in the fill it was located. | | AA-7403 | -10* | 410±60 | [39] at p.164 |
| Garoga | A-42812 | maize kernel | Feature 193, a pit measuring 76 cm deep east of Longhouse 4 ([76] at p.107). Recovered from top of pit in lens of charred vegetal material. | | UCIAMS190540 | -8.0 | 345±20 | [65] |
| Garoga | A-42235-F | maize kernel | Feature 2, pit measuring 106 x 122 cm in plan within Longhouse 9 ([76] at p.98) | | UCIAMS218478 | -9.3 | 345±15 | [65] |
| Garoga | A-42354.E.1 | *Ulmus* sp. RY1003-1004 | Feature 36, a cylindrical pit measuring 122 cm in diameter and 147 cm deep within Longhouse 9 ([76] at p.99). From hearth-like deposit at top of feature. | | UCIAMS-226646 | -27.3 | 345±20 | This study |
| Garoga | A-42235F | maize kernel | Feature 2, pit measuring 106 x 122 cm in plan within Longhouse 9 ([76] at p.98). Field notes indicate a concentration of maize but do not indicate where in the fill it was located. | | UCIAMS190537 | -9.7 | 335±20 | [65] |
| Garoga | A-42801 | maize kernel | Feature 184, a pit measuring 213x168 cm in plan between Longhouse 1 and the stockade ([76] at p.106). Likely recovered from top of pit in dark charcoal-rich layer. | | UCIAMS218477 | -9.1 | 330±20 | [65] |
| Garoga | A-42354.E.1 | *Fagus grandifolia* RY1001-1003 | Feature 36., cylindrical pit measuring 122 cm in diameter and 147 cm deep within Longhouse 9 ([76] at p.99). From hearth-like deposit at top of feature. | | UCIAMS-226648 | -24.7 | 325±20 | This study |
| Garoga | A-42815 | maize kernel | Feature 196, a cylindrical pit measuring 183 cm in diameter and 168 cm deep within Longhouse 2 ([76] at p.107). Maize recovered from a layer of charred vegetal material at the top of the pit. | | UCIAMS190539 | -8.6 | 320±20 | [65] |
| Garoga | A-42354.E.1 | *Fagus* *grandifolia* RY1015-1019 | Feature 36, cylindrical pit measuring 122 cm in diameter and 147 cm deep within Longhouse 9 ([76] at p.99). From hearth-like deposit at top of feature. | | UCIAMS-226652 | -24.8 | 320±20 | This study |
| Garoga | A-42354.E.1 | *Ulmus* sp. RY1049-1053 | Feature 36., cylindrical pit measuring 122 cm in diameter and 147 cm deep within Longhouse 9 ([76] at p.99). From hearth-like deposit at top of feature. | | UCIAMS-226647 | -26.0 | 320±20 | This study |
| Garoga | A-72559 | maize kernel | Feature 103, a pit measuring 168 cm in diameter within Longhouse 12 ([76] at p.102). No notes were found for this feature. | | UCIAMS218479 | -8.6 | 315±15 | [65] |
| Garoga | A-42801 | maize kernel | Feature 184, a pit measuring 213x168 cm in plan between Longhouse 1 and the stockade ([76] at p.106). No indication where sample was recovered from fill. | | UCIAMS190538 | -8.4 | 305±20 | [65] |
| Garoga | A-42235F | maize kernel | Feature 2, pit measuring 106 x 122 cm in plan within Longhouse 9 ([76] at p.98). Field notes indicate a concentration of maize but do not indicate where in the fill it was located. | | AA-6417 | -10* | 300±50 | [39] at p.164 |
| Klock | A-44727 | maize kernel | Feature 106, a hearth measuring 91 x 61 cm in plan and 15 cm deep within Longhouse 7 ([76] at p.26). Sample may have been recovered from lowest stratum on the feature. | | AA-7404 | -10* | 520±75 | [39] at p.171 |
| Klock | A-45738-43 | charred cooking residue adhering to pottery sherd interior | Feature 117, a pit measuring 132 x 122 cm in plan and 130 cm deep within Longhouse 8 ([76] at p.26). From deposit in lower third of fill. | | ISGS-A0523 | -23.6 | 480±40 | [131] |
| Klock | 45171-A | maize cob | Feature 84, a pit feature measuring 107 x 152 cm in plan and 122 cm in depth in the midline of Longhouse 1 ([76] at p.25). From bottom of pit above lining. | | UCIAMS-239712 | -9.5 | 370±15 | This study |
| Klock | A-45171-A | maize kernel | Feature 84, a pit feature measuring 107 x 152 cm in plan and 122 cm in depth in the midline of Longhouse 1 ([76] at p.25). From bottom of pit above lining. | | UCIAMS--218474 | -8.8 | 365±15 | [65] |
| Klock | A-45737-A | maize kernel | Feature 116, a pit measuring 107x123 cm in plan and 64 cm deep within Longhouse 1 ([76] at p.26). No indication from where in the fill the sample was recovered. | | UCIAMS-218476 | -9.1 | 365±15 | [65] |
| Klock | A-45172-C | Unburned disarticulated white tailed deer bone | Feature 86, a pit measuring 117 x 107 cm in plan and 107 cm deep, outside the north wall of Longhouse 5 ([76] at p.25). No indication where in fill the sample was recovered. | | UCIAMS-190559 | -21.3 | 360±15 | [65] |
| Klock | A-45171 | monocot blades | Feature 84, a pit feature measuring 107 x 152 cm in plan and 122 cm in depth in the midline of Longhouse 1 ([76] at p.25). Grass blades from 107 cm depth at bottom of pit. | | UCIAMS-239714 | -24.0 | 360±15 | This study |
| Klock | 45171.A1 | *Ulmus* sp. RY1010-1018 | Feature 84, a pit feature measuring 107 x 152 cm in plan and 122 cm in depth in the midline of Longhouse 1 ([76] at p.25). From upper lens in post-use fill. | | UCIAMS-239721 | -25.8 | 360±15 | This study |
| Klock | A-45136-B | unburned disarticulated white tailed deer bone | Feature 3, a hearth measuring 108 cm in diameter and 15 cm deep within Longhouse 1 ([76] at p.21). Field notes do not indicate where in the hearth the sample was found. | | UCIAMS-190562 | -23.2 | 355±20 | [65] |
| Klock | A-45171.A.1 | *Ulmus* sp. RY1021-1025 | Feature 84, a pit feature measuring 107 x 152 cm in plan and 122 cm in depth in the midline of Longhouse 1 ([76] at p.25). From upper lens in post-use fill. | | UCIAMS-226654 | -24.5 | 355±20 | This study |
| Klock | 45171-A | maize cob | Feature 84, a pit feature measuring 107 x 152 cm in plan and 122 cm in depth in the midline of Longhouse 1 ([76] at p.25). From bottom of pit above lining. | | UCIAMS-239713 | -8.8 | 35015 | This study |
| Klock | A-45149-C | unburned disarticulated white tailed deer bone | Feature 36, a pit measuring 91 x 107 cm in plan east of Longhouse 4 ([76] at p.23). Field notes not located. | | UCIAMS-190561 | -22.6 | 335±15 | [65] |
| Klock | A-45743 | maize kernel | Feature 135, a pit measuring 123 cm in diameter located between Longhouses 3 and 5 ([76] at p.27). Entire pit was filled with fire-cracked rock deposited over a charred bark lining. Field notes indicate recovery of maize from the bark lining. | | UCIAMS-218475 | -9.5 | 335±20 | [65] |
| Klock | 45743.A.1 | *Fraxinus* sp. RY1009-1013 | Feature 135, a pit measuring 123 cm in diameter located between Longhouses 3 and 5 ([76] at p.27). Sample likely came from within the fire-cracked rock deposit. No specific mention of wood charcoal in field notes. | | UCIAMS-239711 | -30.5 | 330±20 | This study |
| Klock | A-45144-A | maize kernel | Feature 20, a pit measuring 122x152 cm in plan and 76 cm deep located outside Longhouse 5 ([76] at p.22). Sample was from a layer of charcoal and charred maize in the upper third of pit fill. | | UCIAMS-218473 | -9.3 | 325±15 | [65] |
| Klock | A-45163-C | unburned disarticulated white tailed deer bone | Feature 65, a hearth measuring 46 cm in diameter within Longhouse 1 ([76] at p.24). No indication of where in hearth the sample was recovered. | | UCIAMS-190560 | -22.1 | 325±15 | [65] |
| Klock | 45157-A | maize cob | Feature 50, a pit feature measuring 114 x 102 cm in plan and 107 cm in depth in the midline of Longhouse 1 ([76] at p.25). From upper lens in post-use fill. | | ISGS-A0326 | -9.0 | 317±38 | [132] |
| Klock | A-44727 | maize kernel | Feature 106, a hearth measuring 91 x 61 cm in plan and 15 cm deep within Longhouse 7 ([76] at p.26). No indication in field notes where in the hearth the sample was recovered. | | AA-6418 | -10* | 315±60 | [39] at p.171 |
| Klock | 45171.A1 | *Ulmus* sp. RY1001-1002 | Feature 84, a pit feature measuring 107 x 152 cm in plan and 122 cm in depth in the midline of Longhouse 1 ([76] at p.25). From upper lens in post-use fill. | | UCIAMS-239720 | -24.1 | 300±15 | This study |
| Klock | A-45171.A.1 | *Ulmus* sp. RY1001-1002 | Feature 84, a pit feature measuring 107 x 152 cm in plan and 122 cm in depth in the midline of Longhouse 1 ([76] at p.25). From upper lens in post-use fill. | | UCIAMS-226653 | -22.8 | 290±20 | This study |
| Smith-Pagerie | A-45604-A | maize kernel | Feature 60, a pit measuring 91 cm in diameter and 61 cm deep within Longhouse 5 ([76] at p.61). Excavation notes not located. | | UCIAMS-218493 | -8.9 | 480±15 | [65] |
| Smith-Pagerie | A-44728-13 | charred cooking residue adhering to pottery sherd interior | Feature 15, a pit measuring 152 x 122 cm in plan and 107 cm deep within Longhouse 1 ([76] at p.59). No indication where in the pit fill the sherd was recovered. | | ISGS-A0528 | -20.7 | 445±40 | [132] |
| Smith-Pagerie | A-44757 | maize kernel | Feature 54, a pit measuring 122 x 152 cm in diameter and 135 cm deep within Longhouse 1 ([76] at p.61). From dark-fill lens in middle of post-use fill. | | AA-7405 | -10* | 430±50 | [39] at p.180 |
| Smith-Pagerie | A-44757 | maize kernel | Feature 54, a pit measuring 122 x 152 cm in plan and 135 cm deep within Longhouse 1 ([76] at p.61). From dark-fill lens in middle of post-use fill. | | AA-6419 | -10* | 405±50 | [39] at p.180 |
| Smith-Pagerie | A-44736 | unburned disarticulated white tailed deer bone | Feature 25, a pit feature measuring 157 x 183 cm in plan and 102 cm deep within Longhouse 1 ([76] at p.60). No indication where in the pit fill the sample was recovered. | | UCIAMS1-90566 | -22.2 | 375±15 | [65] |
| Smith-Pagerie | A-44724 | unburned disarticulated white tailed deer bone | Feature 11, a pit measuring 107 cm in diameter and 152 cm deep in the midline of Longhouse 1 ([76] at p.59). Sample likely recovered from massive deposit filling upper two-thirds of the pit. | | UCIAMS-190565 | -22.6 | 370±15 | [65] |
| Smith-Pagerie | A-44746 | unburned disarticulated white tailed deer bone | Feature 40, a pit measuring 122 x 137 cm in plan and 122 cm deep within Longhouse 1 ([76] at p.60). Excavation notes not located. | | UCIAMS-190563 | -24.0 | 360±20 | [65] |
| Smith-Pagerie | A-45604-A | maize kernel | Feature 80, a hearth within Longhouse 2 ([76] at p.62). No indication of where in the fill the sample was recovered. | | UCIAMS2-18492 | -8.7 | 350±15 | [65] |
| Smith-Pagerie | A-44711 | unburned disarticulated white tailed deer bone | Feature 127, a hearth-like feature measuring 76 cm in diameter and 38 cm deep in the midline of Longhouse 4 ([76] at p.64). No indication of where in the fill the sample was recovered. | | UCIAMS-190564 | -23.4 | 345±20 | [65] |
| Smith-Pagerie | A-44757.1 | *Fagus grandifolia* RY1011-1015 | Feature 54, a pit measuring 122x152 cm in plan and 135 cm deep within Longhouse 1 ([76] at p.61). Collected from dark-stained maize-containing fill lens. | | UCIAMS-226656 | -23.4 | 340±20 | This study |
| Smith-Pagerie | A-44757.1 | *Betula* sp. RY1020 | Feature 54, a pit measuring 122x152 cm in plan and 135 cm deep within Longhouse 1 ([76] at p.61). Collected from dark-stained maize-containing fill lens. | | UCIAMS-239718 | -24.6 | 340±15 | This study |
| Smith-Pagerie | A-44757.1 | *Fagus grandifolia* RY1023-1028 | Feature 54, a pit measuring 122x152 cm in plan and 135 cm deep within Longhouse 1 ([76] at p.61). Collected from dark-stained maize-containing fill lens. | | UCIAMS-226657 | -23.3 | 330±20 | This study |
| Smith-Pagerie | A-70231 | maize kernel | Feature 54, a pit measuring 122x152 cm in plan and 135 cm deep within Longhouse 1 ([76] at p.61). From fill lens in middle of post-use fill. | | UCIAMS-218490 | ** | 325±30 | [65] |
| Smith-Pagerie | A-44757.1 | *Betula* sp. RY1001-1003 | Feature 54, a pit measuring 122x152 cm in plan and 135 cm deep within Longhouse 1 ([76] at p.61) Collected from dark-stained maize-containing fill lens. | | UCIAMS-239716 | -25.8 | 320±20 | This study |
| Smith-Pagerie | A-44757.1 | *Fagus grandifolia* RY1001-1005 | Feature 54, a pit measuring 122x152 cm in plan and 135 cm deep within Longhouse 1 ([76] at p.61). Collected from dark-stained maize-containing fill lens. | | UCIAMS-226655 | -22.4 | 315±20 | This study |
| Smith-Pagerie | A-44757.1 | *Fagus grandifolia* RY1001-1005 | Feature 54, a pit measuring 122x152 cm in plan and 135 cm deep within Longhouse 1 ([76] at p.61). Collected from dark-stained maize-containing fill lens. | | UCIAMS-239715 | -23.0 | 295±15 | This study |
| Smith-Pagerie | A-44722 | unknown | Feature 9, a pit measuring 183x168 cm in plan and 99 cm deep in the midline of Longhouse 1 ([76] at p.59). No indication of where in the fill the sample was recovered. | | UCIAMS-218491 | -29.1 | 265±15 | [65] |
| Brigg’s Run | A2002.10AC.14.7.1 | maize cob | Unidentified pit feature, avocational donation | ISGS-A0328 | | -9.7 | 401±38 | [132] |
| Brigg’s Run | A2002.10AC.14.9.1 | *Fagus grandifolia* RY1003-1004 | Unidentified pit feature, avocational collection | UCIAMS-239718 | | -24.6 | 385±15 | This study |
| Brigg’s Run | A2002.10AC.14.9.1 | *Fagus grandifolia* RY1002 | Unidentified pit feature, avocational collection | UCIAMS-226644 | | -24.9 | 345±20 | This study |
| Brigg’s Run | A2002.10AC.14.9.1 | *Fagus grandifolia* RY1021-1023 | Unidentified pit feature, avocational collection | UCIAMS-239719 | | -25.2 | 320±15 | This study |
| Brigg’s Run |  | maize kernel | Unidentified pit feature, avocational collection | AA-7693 | | -10.0* | 315±40 | [39] at p.259 |
| Brigg’s Run |  | maize kernel | Unidentified pit feature, avocational collection | UCIAMS-226643 | | -8.6 | 315±20 | This study |
| Brigg’s Run | A2002.10AC.14.9.1 | *Fagus grandifolia* RY1023-1025 | Unidentified pit feature, avocational collection | UCIAMS-226645 | | -24.9 | 305±20 | This study |
| Brigg’s Run |  | maize kernel | Unidentified pit feature, avocational collection | AA-7417 | | -10.0* | 290±37 | [39] at p.259 |
