# Supplementary material for: Resolving Indigenous village occupations and social history across the long century of European permanent settlement in Northeastern North America: The Mohawk River Valley ~1450-1635 CE
Source: PLoS One. 2021 Oct 15;16(10):e0258555. doi: 10.1371/journal.pone.0258555 (PMC8519479; doi:10.1371/journal.pone.0258555)
Supplement: S2 Table — (DOCX) [file pone.0258555.s002.docx]

**S2 Table. Comparison of summary of results from Model 1,** LnN(ln(20),ln(2))**, Interval query constraint, Model 2,** N(20,10)**, Interval query constraint, and Model 3,** N(25,10)**, Interval query constraint for the Smith-Pagerie, Klock, Garoga and Brigg’s Run sites and queries related to two specific features at these sites (House, H, 9 at Garoga and House, H, 1 at Klock).**

|  | **A_model_** | **68.3% hpd** | **95.4% hpd** |  | **A_model_** | **68.3% hpd** | **95.4% hpd** |  | **A_model_** | **68.3% hpd** | **95.4% hpd** |
| --- | --- | --- | --- | --- | --- | --- | --- | --- | --- | --- | --- |
| **Smith-Pagerie Model 1** | 111 |  |  | **Smith-Pagerie Model 2** | 108 |  |  | **Smith-Pagerie Model 3** | 108 |  |  |
| Boundary Start |  | 1475-1492 | 1469-1504 | Boundary Start |  | 1475-1492 | 1469-1505 | Boundary Start |  | 1475-1492 | 1469-1504 |
| Date |  | 1478-1498 | 1468-1512 | Date |  | 1478-1499 | 1469-1511 | Date |  | 1478-1499 | 1469-1511 |
| Boundary End |  | 1485-1506 | 1479-1521 | Boundary End |  | 1486-1507 | 1479-1519 | Boundary End |  | 1487-1507 | 1479-1519 |
| *Interval* |  | *5-22* | *2-44* | *Interval* |  | *7-27* | *0-35* | *Interval* |  | *7-27* | *0-35* |
| **Klock Model 1a** | 53 |  |  | **Klock Model 2a** | 53 |  |  | **Klock Model 3a** | 55 |  |  |
| Boundary Start |  | 1485-1513 | 1472-1517 (77.5) 1563-1595 (18.0) | Boundary Start |  | 1487-1510 | 1478-1517 (80.5) 1569-1593 (15.0) | Boundary Start |  | 1483-1510 | 1475-1516 (76.9) 1564-1593 (18.6) |
| Date H1 |  | 1497-1518 | 1489-1523 (77.7) 1579-1604 (17.8) | Date H1 |  | 1497-1518 | 1490-1522 (80.7) 1582-1603 (14.5) | Date H1 |  | 1496-1518 | 1489-1523 (76.9) 1579-1605 (18.6) |
| Date |  | 1494-1523 | 1483-1532 (77.2) 1573-1611 (18.2) | Date |  | 1495-1521 | 1486-1530 (80.4) 1576-1606 (15.1) | Date |  | 1492-1524 | 1484-1532 (76.7) 1574-1610 (18.7) |
| Boundary End |  | 1505-1531 | 1498-1543 (77.4) 1587-1624 (17.9)  1626-1627 (0.2) | Boundary End |  | 1507-1529 | 1498-1538 (80.8) 1590-1615 (14.7) | Boundary End |  | 1507-1533 | 1500-1541 (77.1) 1590-1620 (18.3) |
| *Interval* |  | *5-26* | *3-53* | *Interval* |  | *10-30* | *1-38* | *Interval* |  | *14-34* | *5-44* |
| **Klock Model 1b** | 68 |  |  | **Klock Model 2b** | 69 |  |  | **Klock Model 3b** | 71 |  |  |
| Boundary Start |  | 1487-1512 | 1470-1517 (82.4) 1565-1594 (13.1) | Boundary Start |  | 1487-1510 | 1478-1517 (82.0) 1569-1592 (13.4) | Boundary Start |  | 1485-1508 | 1474-1516 (84.2) 1567-1591 (11.2) |
| Date H1 |  | 1498-1518 | 1489-1523 (82.6) 1580-1603 (12.9) | Date H1 |  | 1498-1517 | 1489-1523 (81.9) 1581-1603 (13.5) | Date H1 |  | 1497-1516 | 1488-1523 (84.5) 1583-1603 (11.0) |
| Date |  | 1496-1521 | 1482-1533 (82.0) 1574-1610 (13.4) | Date |  | 1496-1521 | 1485-1530 (81.8) 1576-1606 (13.7) | Date |  | 1495-1521 | 1483-1532 (84.2) 1577-1607 (11.2) |
| Boundary End |  | 1506-1529 | 1497-1545 (82.4) 1589-1623 (13.1) | Boundary End |  | 1507-1529 | 1498-1539 (82.2) 1591-1614 (13.2) | Boundary End |  | 1509-1531 | 1500-1541 (84.5) 1593-1616 (10.9) |
| *Interval* |  | *5-27* | *2-53* | *Interval* |  | *10-30* | *2-39* | *Interval* |  | *14-34* | *5-44* |
| **Garoga Model 1** | 114 |  |  | **Garoga Model 2** | 111 |  |  | **Garoga Model 3** | 109 |  |  |
| Boundary Start |  | 1541-1573 | 1502-1626 (10.4) 1529-1583 (77.5) 1612-1629 (7.6) | Boundary Start |  | 1543-1571 | 1503-1524 (9.1) 1533-1581 (79.4) 1612-1628 (6.9) | Boundary Start |  | 1539-1569 | 1500-1524 (10.8) 1529-1578 (78.6) 1609-1625 (6.1) |
| *Duration H9* |  | *0-8* | *0-21* | *Duration H9* |  | *0-8* | *0-19* | *Duration H9* |  | *0-10* | *0-22* |
| Date |  | 1550-1582 | 1513-1593 (88.0) 1619-1636 (7.4) | Date |  | 1551-1581 | 1512-1534 (8.3) 1539-1593 (80.3) 1619-1635 (6.8) | Date |  | 1549-1582 | 1512-1593 (89.9) 1619-1635 (5.5) |
| Boundary End |  | 1560-1590 | 1523-1541 (7.4) 1543-1547 (0.8) 1549-1549 (0.1) 1554-1602 (79.1) 1627-1642 (8.1) | Boundary End |  | 1561-1588 | 1524-1543 (8.3) 1555-1600 (79.8) 1628-1643 (7.3) | Boundary End |  | 1563-1592 | 1525-1549 (10.1) 1555-1602 (79.1) 1629-1644 (6.3) |
| *Interval* |  | *5-24* | *2-50* | *Interval* |  | *8-27* | *1-37* | *Interval* |  | *13-33* | *4-43* |
| **Brigg’s Run Model 1** | 70 |  |  | **Brigg’s Run Model 2** | 67 |  |  | **Brigg’s Run Model 3** | 66 |  |  |
| Boundary Start |  | 1610-1627 | 1564-1582 (8.7) 1593-1631 (86.7) | Boundary Start |  | 1608-1627 | 1570-1571 (0.1) 1588-1634 (95.3) | Boundary Start |  | 1602-1624 | 1579-1631 |
| Date |  | 1619-1632 | 1585-1588 (0.9) 1593-1634 (94.6) | Date |  | 1619-1632 | 1599-1635 | Date |  | 1616-1632 | 1593-1634 |
| Boundary End |  | 1629-1635 | 1623-1635 | Boundary End |  | 1629-1635 | 1622-1635 | Boundary End |  | 1629-1635 | 1592-1593 (0.2) 1621-1635 (95.3) |
| *Interval* |  | *4-20* | *2-46 (93.3)*  *47-48 (0.4)*  *59-65 (1.8)* | *Interval* |  | *4-22* | *0-33* | *Interval* |  | *8-29* | *1-41* |
