# Supplementary material for: Resolving Indigenous village occupations and social history across the long century of European permanent settlement in Northeastern North America: The Mohawk River Valley ~1450-1635 CE
Source: PLoS One. 2021 Oct 15;16(10):e0258555. doi: 10.1371/journal.pone.0258555 (PMC8519479; doi:10.1371/journal.pone.0258555)
Supplement: S1 File — Note: in S1 File the measurements are given in feet (‘) and inches (”) as listed in the original site documentation and drawings (some reproduced below). (DOCX) [file pone.0258555.s003.docx]

**S1 File. Field drawings and transcribed notes for features described in the article text. Field drawings have been cropped to remove individual’s names.** Note: in Supplemental File S1 the measurements are given in feet (’) and inches (”) as listed in the original site documentation and drawings (some reproduced below).

***Garoga***

Pit 36 Garoga 8/14/61


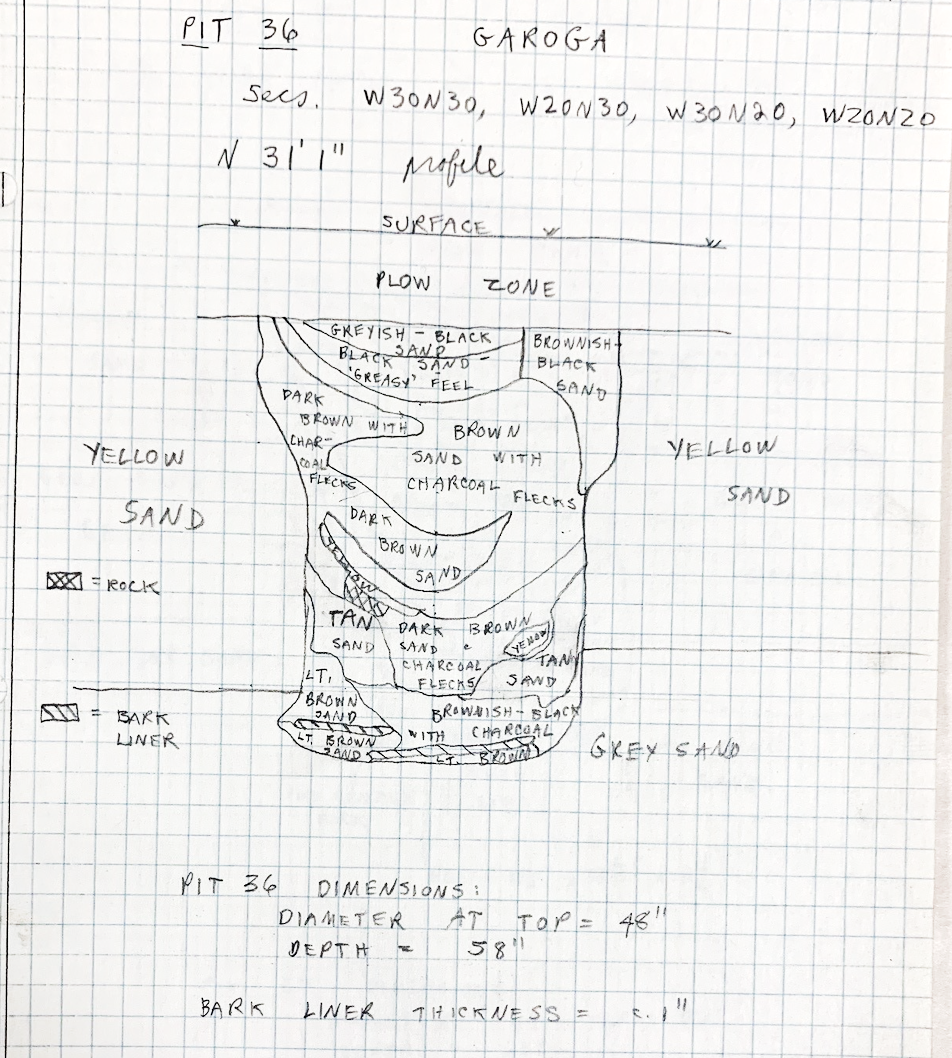


**Figure A**. Field-drawn profile of Garoga Feature 36 (1 square = 4 inches). Courtesy New York State Museum.


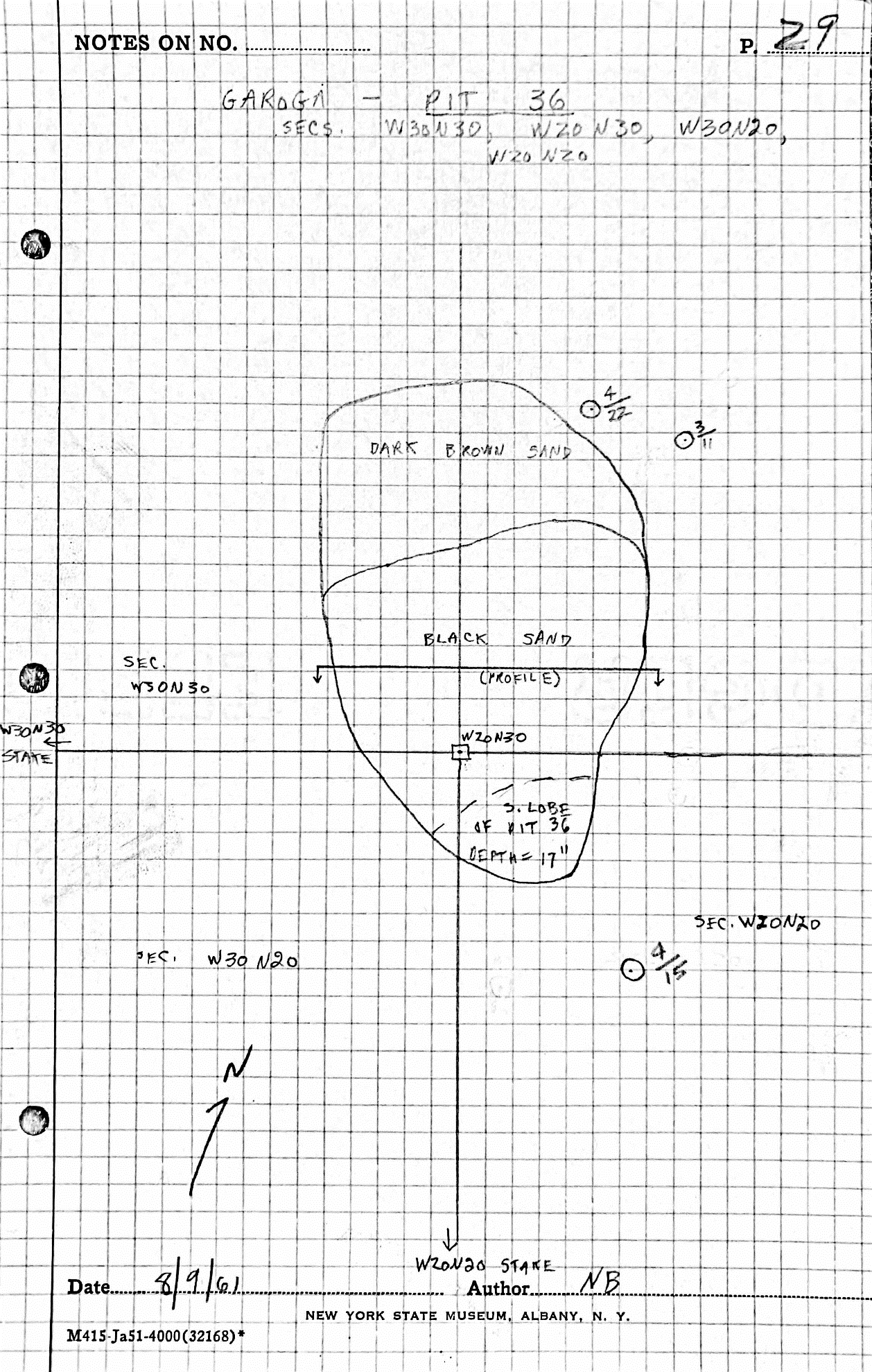


**Figure B**. Field plan map of Garoga Feature 36 (1 square = 3 inches). Courtesy New York State Museum).

Pit 36, which was 58" deep and 48" in diameter, was completed today. Less than 2 boxes of artifacts were found in the pit. Artifacts consisted of mainly pottery, but one beautiful complete projectile point of the Madison type was found in the south lobe of the pit at a depth of 15". This point measured c. 1¾" in length.

Pit 36 had a shallow lobe on its south site (see drawing of the top of the pit—horizontal) which reached a maximum depth of 17". Many fire cracked rocks, charcoal, ash, a few sherds, and one beaver jaw were found within this southern lobe. This lobe seemed to have been a fire area, where the above mentioned projectile point was probably accidentally dropped. The lobe consisted of sand mainly black in color.

On the horizontal drawing of Pit 36, dark brown sand and black sand can be seen at the top of the pit. The dark brown area intergraded with the black sand.

Pit 36 contained a definite bark liner near the bottom. The liner was unevenly placed. That is, it was not placed on the same level (see profile drawing), a photograph was taken of the bark liner on 8/10.

Corn kernels, acorns, and a few other types of seed were found within the bark liner.

The liner was about 1" in thickness and was collected in cigar boxes.

Pottery was found beneath the bark linter. Several fire cracked rocks were found immediately on top of the liner. Other stones found on the liner included a stone hoe measuring 9" x 8" x 1½" and a large rock (10" x 11" x 3½") which has a few chips taken off one side. This rock is quite heavy, but may have been used as a digging implement, as was probably the hoe just mentioned.

Some mammal bones were also found in Pit 36. One large piece of a skull was found above the bark liner.

***Klock***

Feature 50


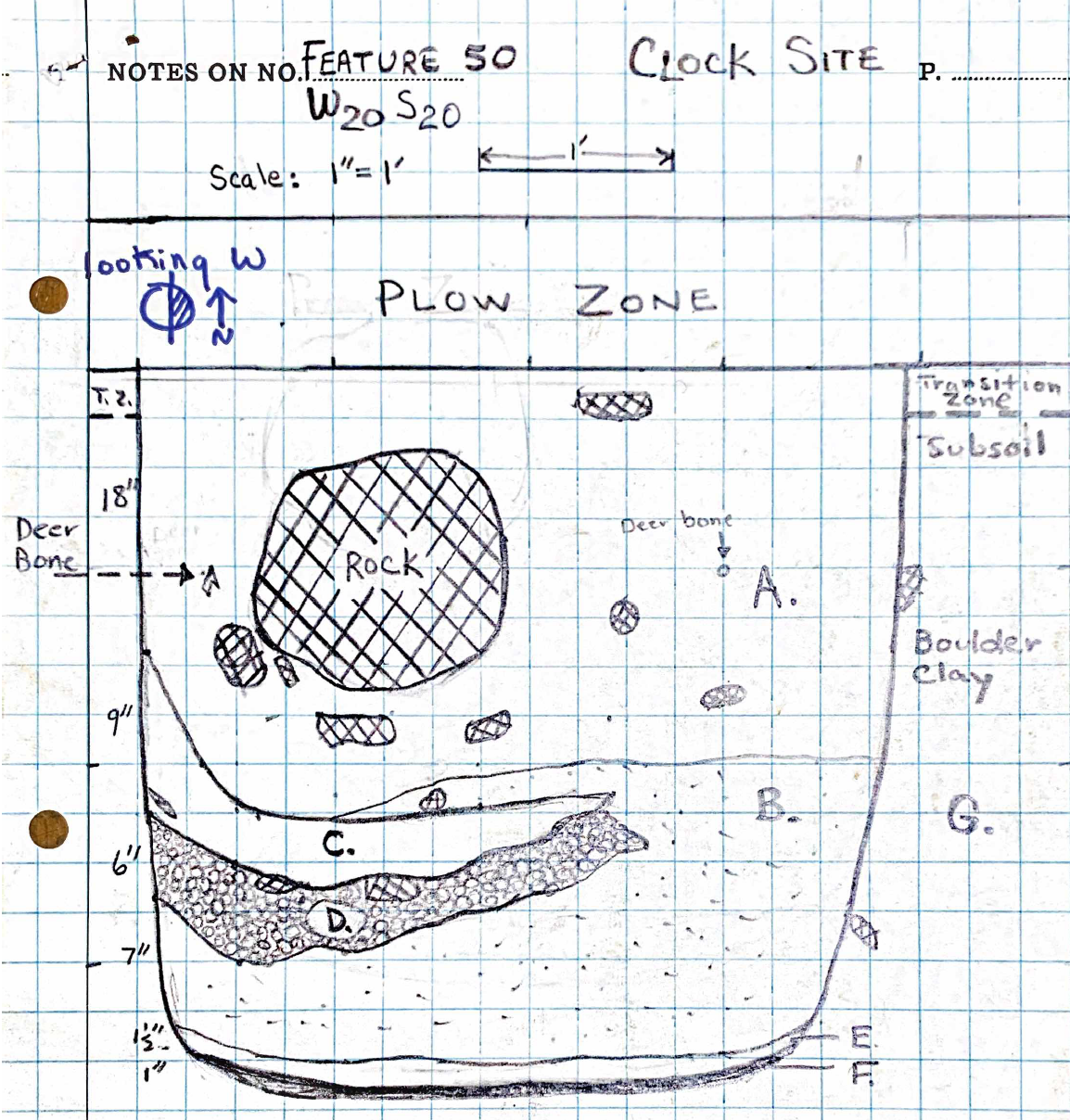


**Figure C**. Field-drawn profile of Klock Feature 50 (1 square = 3 inches). Courtesy New York State Museum.

A. Dark gray/brown to black/brown fill. 1 Artifact found—anvil hammerstone plus flint chips, charcoal, some bone and shell. Large rock must have been rolled in by the Mohawk and was not in situ.

B. Dark gray soil fill. Contained greater amounts of charcoal, flint ships, bone, shell, plus a scattering of a few pot sherds, carbonized corn, beans, bark, and wood.

C. Yellow ochre soil fill. Probably thrown in as a sanitation layer over D. A crude mudstone ornament was found in the bottom left corner of this fill.

D. Carbonized corn—black. This lens is composed entirely of corn. Some cobs were extracted with kernels still on them. This lens in connection with C seem to identify this feature as a storage pit. The corn either spoiled or was infested with vermin. The Indians probably burned this corn out and then threw in layer C to cover the burned corn. Some pot sherds and bark were found in close connection with the corn. This may have been containers for the corn.

E. Orange burned soil and fire cracked rocks.

F. Fiber layer—black. This marks the bottom of the pit. It consists of carbonized grass or weaving (mat) which was probably put in to line the pit.

G. Subsoil—yellow ochre in color—All boulder clay. Filled with decaying rocks including shale, mudstone, and a =rock that resembles hematite.

Depth of pit: 3'7"

Width at top 3'11"

Feature 50 was roughly circular at the surface with a diameter of four feet

The plow zone above it was 8 to 10 inches deep.

Feature 50 was used as a storage pit. A layer of carbonized corn and cobs was discovered in it. The corn in this pit either went sour or became infested with vermin. The Indians then burned out the pit and then in a sanitation layer of yellow ochre sand over the debris. The corn layer averaged about four inches thick.

The pit went down to a depth of 3' 7" where a carbonized layer of grass, and/or corn husk, and/or bark mat or weaving was discovered. This was the lining of the pit. This layer was about 1" thick.

The sides of the pit went almost straight down except for the northwest wall whick belled out at a depth of 10 inches. The bell was about five inches wide. A deer skull and some pot sherds were found in the bell. The skull showed signs of its antlers being broken off.

The pit contained a large number of stones and fire-cracked rocks, including 1 250 pound boulder.

Besides rocks and a large amount of corn kernels, cobs, and stalks, the pit contained:

DEER BONE-including a number of scapulas

Teeth—including beaver, dog, and deer

Carbonized beans and nuts

4 Madison points—either broken or unfinished

1 antler/bone awl—with grooving for decoration

1 crude mudstone ornament 1" thick & 1 ½" wide with a hole in it. Could have been worn on a thing

Miscellaneous small bones—bird and/or fish and/or small mammals

Pot sherds—both rim and body sherds including Fonda Incised and Cayadutta Incides

1 anvil hammerstone

10 hammer and/or boiling stones—which were found in close association

Approximately 15 cigar boxes of corn remains were removed from the pit.

At least 2 cigar boxes worth of individual kernels was not removed from the pit because it would have been too time consuming.

About 1/3 of the mat lining was saved and wrapped in aluminum foil. Because of its fragility it was impractical to save the entire lining.

Also found were small flint chips and a flint core from which flakes had been struck.

This pit appears to have been located beneath the beds of the longhouse in which it was located.

Feature 84


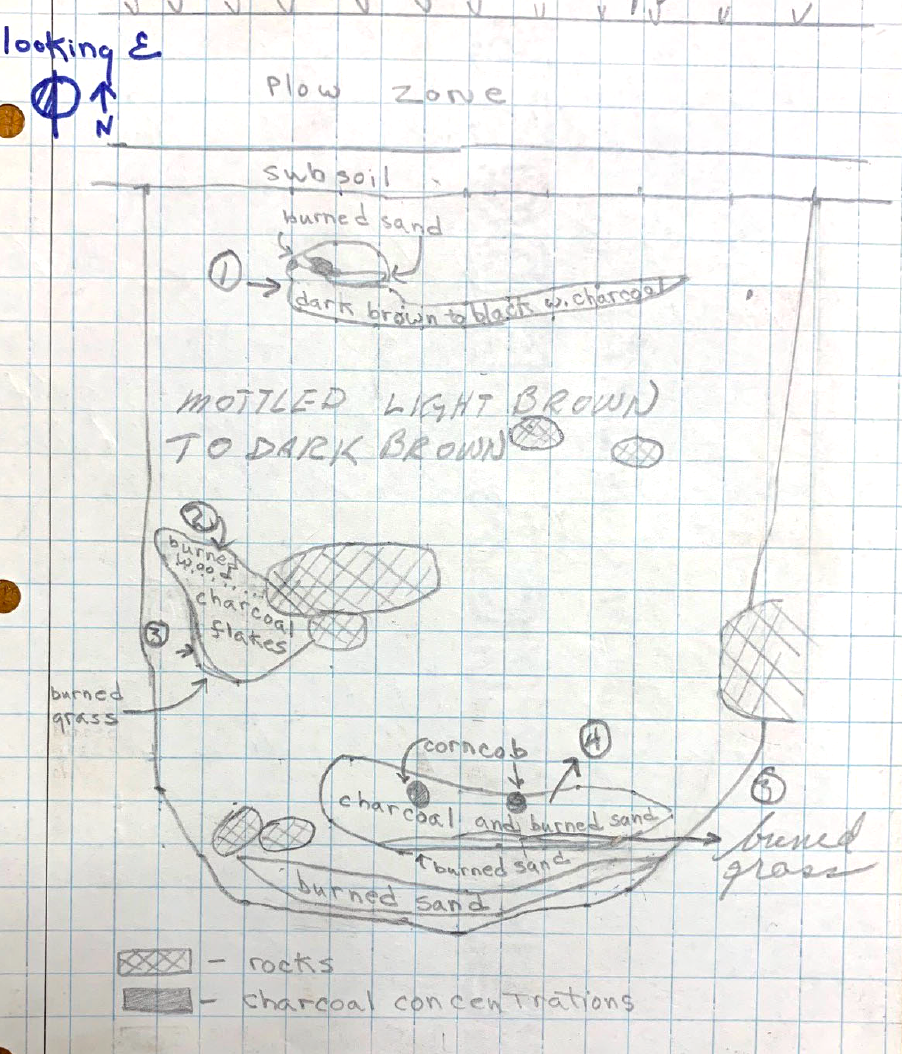


**Figure D**. Field-drawn profile of Klock Feature 84 (1 square = 3 inches). Courtesy New York State Museum.

Clock Site FEA. 84

8/11/69

1. Area may have been the top of pit which was compacted.

2. Contained pieces of partially burned bark (pit lining).

3. Contained burned grass, which may have been pit lining. [Note in container with foil-wrapped grass lining sample indicates: “Klock, F. 84, 8/6/69, 3 ½', grass”]

4. May have been hearth with cleanings. Contained two corn cobs w/ kernels still intact.

Sides and bottom of pit (as well as the interior) had many rocks 3" to 14" long (diameter).

Pit cross-sectioned in the N-S grid line direction or plane. Yielded few clam shells, much deer bone, rodent skull, and small and relatively few pot sherds.

Quite an amt. of corn and cob came from area 4. Pit surrounded by boulder clay and pit delineation was easy.

Contained one hammerstone some flint from a few [unintelligible] to several in’s long.

To be used in conjunction w/ x-sec. map of fea. 84.

1. Rock continued to be scattered throughout the pit; seemed to indicate less of a lining pattern than previously supposed. Fire cracked rock was fairly abundant; diameters of rocks varied from 2" to 8/9"

2. This half of the pit yielded 1 bone awl, 2 antler flakers; 1 back punch (?) made from bone or antler; 2 thumb-grooved hammerstones, much deer bone, some clam shell, few flint chips, and much pottery in the upper half of this half of the x-sec, thinning to very little in the lower quarter; some few bone bangles.

3. In the 3'5" area much corn still on the cob, some corn stalks; recovery of corn is est. at 20%. Found what seemed to be walnuts (wild) numbering 6 or 7.

4. lining of pit was most abundant on the east and N-E walls where it extended 2'3" to 3' 7" deep. Lining was a grass species and probably elm bark too.

5. Uncontaminated samples of grass lining, bark, and corn suitable for dating and wrapped in aluminum were saved.

6. Areas 2 to 4 had red burned sand adjacent to the burned bark and grass lining suggesting that the pit was burned out possibly because the contents had become vermin infested. Burned sand was found immediately above and below the lining.

7. The lowest area of burned sand in the x-sec. may have been hearth cleanings because although there was a concentration in the bottom x-sec. these thinned out and became spots of ash and burned sand at the same level.

Klock 1970

Feature 135. 7/26/70

This feature is clearly a pit, one of the few found in our 30' x 70' longhouse. The top is almost circular, 3'9" at its widest point and flat bottomed with a depth of 4'3".

In several ways this feature is of a strange nature. It contained a tremendous amount of fire-cracked rock (ca. 10 bushels) and the rock was all of larger size than is normally found. There was no lensing to the rock, and the quantity made x-sectioning impossible. The only notable lens was a fire-reddened sand layer which occurred at a depth of 3'8".

Aside from the strange quality and quantity of fire-cracked rock, the pit has all the symptoms of being a classic Iroquois storage/refuse feature. It has a lining of vegetable material, a good sample of which was saved. It contains other perishable material including corn & beans. Artifacts include two classic Iroquois points, two bone awls, 1 celt, 1 antler flaker, numerous sherds including the rim frag’s. from one pot, some small amount of shell, and a good amount of animal bone.

***Smith-Pagerie***

Feature 54

yellow wash


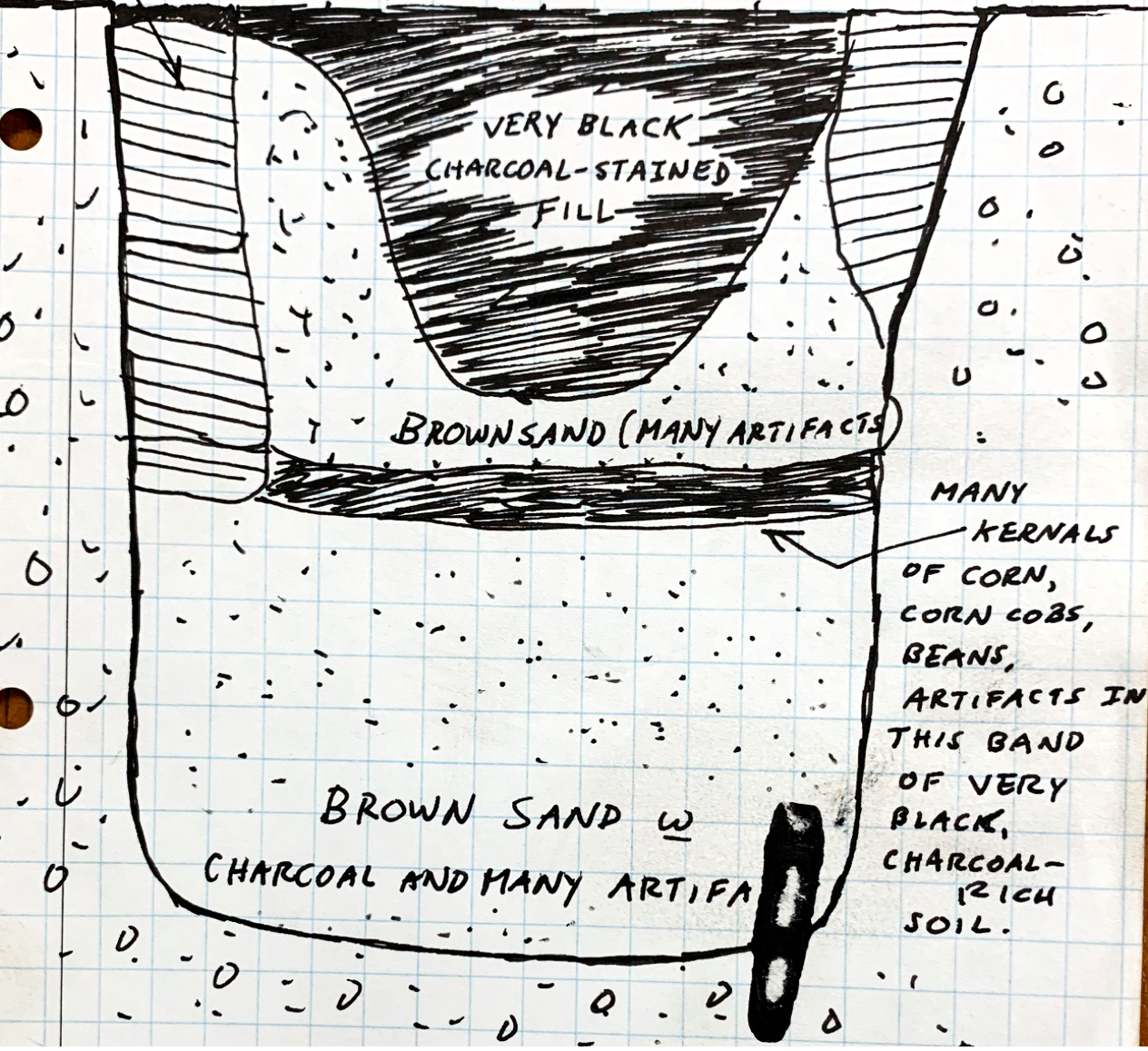


**Figure E**. Field-drawn profile of Smith-Pagerie Feature 54 (1 square= 3 inches). Courtesy New York State Museum.

23 August ‘68

Charcoal from feature 54, Smith Site.

The sample was gathered from 2'6"-3' deep into the feature.

The charcoal lumps were scattered throughout darkly stained (stained black) sand that produced numerous potsherds, corn and beans and many artifacts of bone and stone.

Use only the large lumps to date.

Feature 60. August 13, 1970.


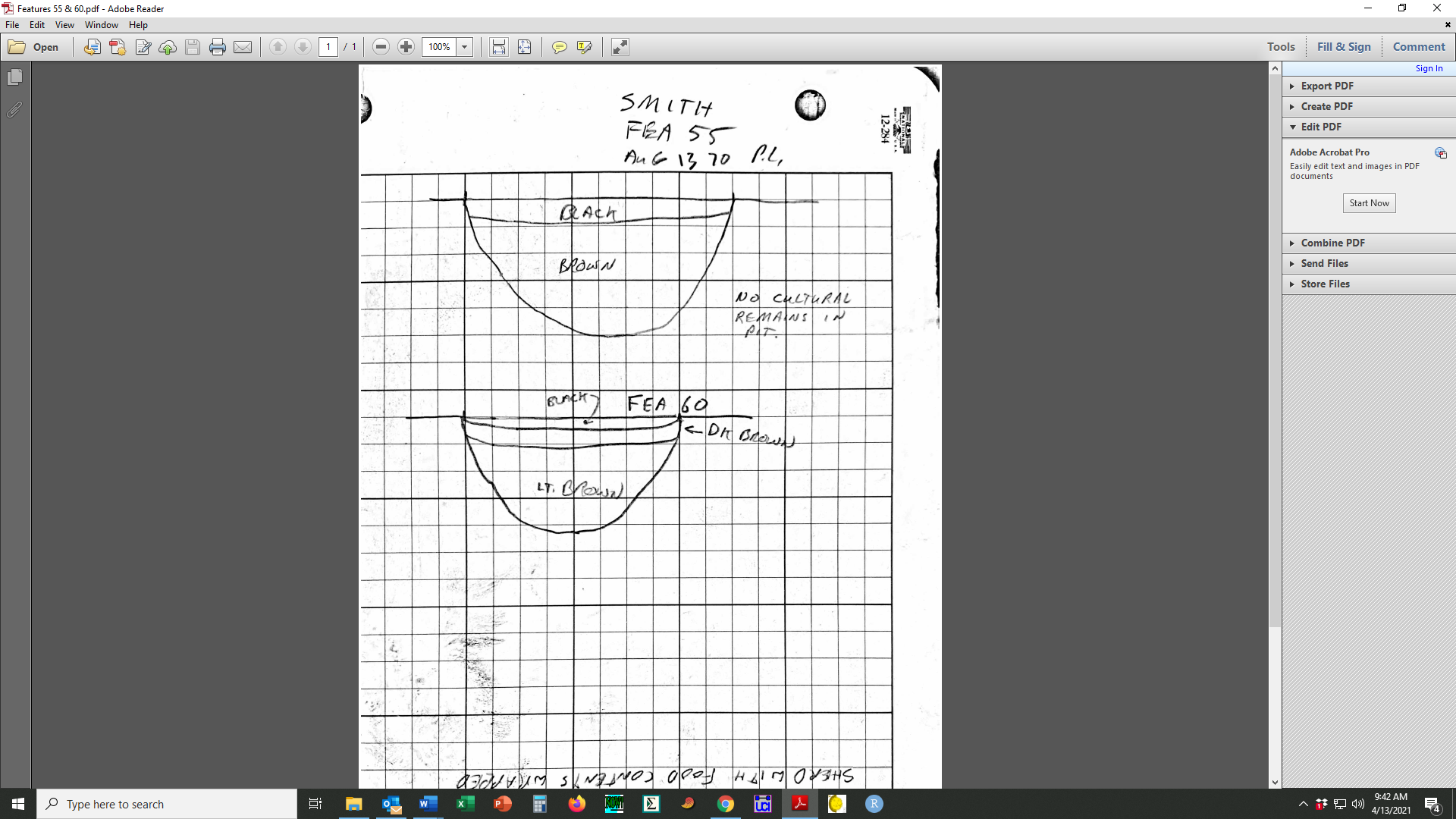


**Figure F**. Field-drawn profile of Smith-Pagerie Feature 54 (1 square = ~4.5 inches). Courtesy New York State Museum.

No field notes were found for this feature.
