# Supplementary material for: Resolving Indigenous village occupations and social history across the long century of European permanent settlement in Northeastern North America: The Mohawk River Valley ~1450-1635 CE
Source: PLoS One. 2021 Oct 15;16(10):e0258555. doi: 10.1371/journal.pone.0258555 (PMC8519479; doi:10.1371/journal.pone.0258555)
Supplement: S2 File — Note: some of the labels/descriptions are edited for display in Figs 5–8. Note: Zea mays (maize) samples are identified as M, Odocoileus virginianus (white-tailed deer) samples are identified as B, and organic residue samples are identified as R. For details, see S1 Table. Note, for reasons of display space, samples of Fagus grandifolia (beech) are listed just as “Fagus”. (DOCX) [file pone.0258555.s004.docx]

**S2 File. OxCal runfiles for the models in Figs. 5-8 and Fig. 9.** Note: some of the labels/descriptions are edited for display in Figs. 5-8. Note: *Zea mays* (maize) samples are identified as M, *Odocoileus virginianus* (white-tailed deer) samples are identified as B, and organic residue samples are identified as R. For details, see Table S1. Note, for reasons of display space, samples of *Fagus grandifolia* (beech) are listed just as “Fagus”.

***Fig. 5. Smith-Pagerie (S-P)***

Options()

{

Resolution=1;

kIterations=3000;

};

Plot()

{

Outlier_Model("General",T(5),U(0,4),"t");

Outlier_Model("Charcoal",Exp(1,-10,0),U(0,3),"t");

Outlier_Model("SSimple",N(0,2),0,"s");

Sequence()

{

Boundary();

Phase("S-P")

{

Sequence()

{

Boundary("Start S-P");

//Phase("F60 pit: initial/only use - assumed start/early in site occupation as clearly early")

//{

// R_Date("UCIAMS218493 M F60 Longhouse 5 Pit",480,15)

// {

// Outlier("General",0.05);

// };

//Small shallow pit 36" diameter and only 24" deep. Thus assume likely just one real use episode. Assume the maize gives a date for the start of the pit and assume - based on 14C - that this is early in (even start of) site/house history. But, even so, ~98% probability outlier. ~100% outlier if included in next Phase. Thus we exclude. Not known why a few decades too old. Separate, earlier activity?

// };

Phase("Pits House 1")

{

R_Date("ISGS-A0528 R F15 H1",445,40)

{

Outlier("Charcoal",1);

};

R_Date("UCIAMS190566 B F25 H1",375,15)

{

Outlier("General",0.05);

};

R_Date("UCIAMS190565 B F11 H1",370,15)

{

Outlier("General",0.05);

};

R_Date("UCIAMS190563 B F40 H1",360,20)

{

Outlier("General",0.05);

};

//R_Date("UCIAMS218491 Material?? F9 pit H1",265,15)

//{

// Outlier("General",0.05);

//};

//Exclude. 1. We do not know what material this sample comprised, so suspect. 2. the d13C value is rather different from all the others (site and whole Mohawk set) at -29.1. 3. If it is included in model, as is, it is a ~100% outlier. Far too recent for rest of site. Hence exclude.

Sequence("F54 samples from Dark Lens")

{

Boundary();

Phase("F54 Dark Lens residual to current maize samples")

{

R_Date("AA-7405 M F54 H1",430,50)

{

Outlier("General",0.05);

};

R_Date("AA-6419 M F54 H1",405,50)

{

Outlier("General",0.05);

};

R_Date("UCIAMS218490 M F54 H1",325,30)

{

Outlier("General",0.05);

};

};

Boundary("Dark Lens Firing");

Before("Before Date of firing, from bark/waney wedge, of Dark Lens in F54")

{

D_Sequence("S-P 4457.1 Fagus 1 F54 G2 H1 28 rings")

{

R_Date("RY1001-1005",315,20)

{

Outlier("SSimple",0.05);

};

Gap(10);

R_Date("RY1011-1015",340,20)

{

Outlier("SSimple",0.05);

};

Gap(12.5);

R_Date("RY1023-1028",330,20)

{

Outlier("SSimple",0.05);

};

Gap(2.5);

Date("S-P Fagus 1 Waney Edge");

};

D_Sequence("S-P 4457.1 Betula sp. F54 G2 H1 20 rings")

{

R_Date("RY1001-1003",320,20)

{

Outlier("SSimple",0.05);

};

Gap(18);

R_Date("RY1020 Betula sp. Bark",340,15)

{

Outlier("SSimple",0.05);

};

};

D_Sequence("S-P Fagus 2 F54 G2 H1 27 rings")

{

R_Date("UCIAMS239715 RY1001-1005",295,15)

{

Outlier("SSimple",0.05);

};

Gap(24);

Date("S-P Fagus 2 Waney Edge");

};

};

};

};

Boundary("Pits to Hearth, equals late/last occupation at site");

Phase("House 2 Hearth")

{

R_Date("UCIAMS218492 M F80 Longhouse 2 Hearth",350,15)

{

Outlier("General",0.05);

};

};

};

Date("Date Smith-Pagerie");

Interval("Interval Smith-Pagerie",LnN(ln(20),ln(2)));

};

Boundary("End S-P");

Boundary("Site use to post-site use");

Phase("Unburnt bone - post site occupation")

{

R_Date("UCIAMS190564 B unburnt F127 Longhouse 4 Hearth",345,20)

{

Outlier("General",0.05);

};

};

Boundary();

};

};

***Fig. 6. Klock.*** *Note: in some cases model runs had issues with (clearly spurious) late very minor possible probability (post early 17^th^ century), hence a simple solution is to add an overall very conservative date constraint to the end Boundary for the model, e.g. a uniform probability constraint specifying ages only between 1300 and 1700 changing the final Boundary line to Boundary("End Klock",U(1300,1700));*

Options()

{

Resolution=1;

kIterations=3000;

};

Plot("Klock")

{

Outlier_Model("General",T(5),U(0,4),"t");

Outlier_Model("Charcoal",Exp(1,-10,0),U(0,3),"t");

Outlier_Model("SSimple",N(0,2),0,"s");

//50% add to SDs in D_Sequence – see Methods

D_Sequence("Klock 45171.A1 Ulmus sp. F84 in H1")

{

Outlier ("SSimple",0.05);

R_Combine("RY1001-1002")

{

R_Date("RY1001-1002 UCIAMS226653",290,30)

{

Outlier ("SSimple",0.05);

};

R_Date("RY1001-1002 UCIAMS239720",300,23)

{

Outlier ("SSimple",0.05);

};

};

Gap(12.5);

R_Date("RY1010-1018 UCIAMS239721",360,23)

{

Outlier ("SSimple",0.05);

};

Gap(9);

R_Date("RY1021-1025 UCIAMS226654",355,30)

{

Outlier ("SSimple",0.05);

};

Gap(2);

Date("Ulmus sp. Bark Last Use F84");

};

Sequence()

{

Boundary("Start Klock");

Phase("Klock")

{

Sequence("House 1")

{

Boundary("Start H1");

Phase ("H1")

{

Sequence("F84 earlier")

{

Boundary("Start F84 Pit");

Phase("Pit Lining equals Construction")

{

R_Date("UCIAMS-239714 Monocot F84 lining",360,15)

{

Outlier("SSimple",0.05);

};

};

Boundary("Making pit to use of F84");

Phase("Initial Use of F84")

{

R_Combine("Feature 84 Pit base H1 - assume one event")

{

Outlier("General",0.05);

R_Date("UCIAMS218474 M F84",365,15)

{

Outlier("SSimple",0.05);

};

R_Date("UCIAMS-239712 M F84",370,15)

{

Outlier("SSimple",0.05);

};

R_Date("UCIAMS-239713 M F84",350,15)

{

Outlier("SSimple",0.05);

};

};

};

Boundary("End Early Use F84");

};

Sequence()

{

Boundary();

Phase("F116 Pit from House 1")

{

R_Date("UCIAMS218476 M F116 Pit H1",365,15)

{

Outlier("General",0.05);

};

};

Boundary();

};

Sequence()

{

Boundary();

Phase("Use H1")

{

R_Date("UCIAMS190562 B Hearth H1",355,20)

{

Outlier("General",0.05);

};

R_Date("UCIAMS190560 M F65 Hearth H1",325,15)

{

Outlier("General",0.05);

};

R_Date("ISGS-A0326 M F50 H1", 317, 38)

{

Outlier("General",0.05);

};

};

Boundary();

};

Date("Date House 1");

Before("Bark Last Use F84")

{

Date("=Ulmus sp. Bark Last Use F84");

};

};

Boundary("End Use H1");

};

Sequence()

{

Boundary();

Phase("Other Klock Dates")

{

R_Date("ISGS-A0523 R F117 H8 Pit",480,40)

{

Outlier("Charcoal",1);

};

R_Date("UCIAMS190559 B H86 Pit",360,15)

{

Outlier("General",0.05);

};

R_Date("UCIAMS190561 B F36 H4 Pit",335,15)

{

Outlier("General",0.05);

};

R_Date("UCIAMS218473 B F20 Pit",325,15)

{

Outlier("General",0.05);

};

R_Date("AA-6418 M Hearth F106 H7", 315, 60)

{

Outlier("General",0.05);

};

Phase("F135 btw H3&5")

{

R_Date("UCIAMS218475 M F135 Pit",335,20)

{

Outlier("General",0.05);

};

D_Sequence("Klock Fraxinus sp. F135 btw H3&5 13 rings bark 2nd list")

{

R_Date("UCIAMS239711 RY1009-1013",330,20);

Gap(2);

Date("F135 bark");

};

};

};

Boundary();

};

Date("Date Klock Overall");

Interval("Interval Klock Overall",LnN(ln(20),ln(2)));

};

Boundary("End Klock");

};

};

***Fig. 7. Garoga***

Options()

{

Resolution=1;

kIterations=3000;

};

Plot()

{

Outlier_Model("General",T(5),U(0,4),"t");

Outlier_Model("Charcoal",Exp(1,-10,0),U(0,3),"t");

Outlier_Model("SSimple",N(0,2),0,"s");

D_Sequence("Garoga 42354.E.1 F36 H9 Ulmus sp. 53 rings no bark")

{

R_Date("RY1003-1004",345,20)

{

Outlier ("SSimple",0.05);

};

Gap(47.5);

R_Date("RY1049-1053",320,20)

{

Outlier ("SSimple",0.05);

};

Gap(2);

Date("Garoga Ulmus sp. TPQ last extant");

};

D_Sequence("Garoga 42354.E.1 F36 H9 Fagus 19 rings to Waney Edge")

{

R_Date("RY1001-1003",325,20)

{

Outlier ("SSimple",0.05);

};

Gap(15);

R_Date("RY1015-1019",320,20)

{

Outlier ("SSimple",0.05);

};

Gap(2);

Date("Garoga Waney Edge");

};

Sequence()

{

Boundary("Start Garoga");

Phase("Garoga")

{

Sequence("House 9")

{

Boundary("Start H9");

Phase("House 9")

{

R_Date("Y-1381 F11 H9 charred wood",620,100)

{

Outlier("Charcoal",1);

};

//R_Date("AA-8370 M F37 Pit H9",585,40)

//{

// Outlier("General",0.05);

//};

//100% outlier when included and individual A value ca.5.4. Exclude.

Phase("F2 Pit H9")

{

// R_Date("AA-7695 M F2 Pit H9",431,39)

// {

// Outlier("General",0.05);

// };

//Outlier ca. 10% and individual A value ca. 8.2. Main reason even excluding AA-8370 that Model overall A value <60. Exclude.

//Model with outlier models applied achieves similar result re date estimate for site when these two dates left in, just poor Amodel value.

R_Date("AA-7403 M F2 Pit H9",410,60)

{

Outlier("General",0.05);

};

R_Date("UCIAMS218478 M F2 Pit H9",345,15)

{

Outlier("General",0.05);

};

R_Date("UCIAMS190537 M F2 Pit H9",335,20)

{

Outlier("General",0.05);

};

R_Date("AA-6417 M F2 Pit H9",300,50)

{

Outlier ("General",0.05);

};

};

};

Sequence("F36 Late Lobe TAQ H9")

{

Date("=Garoga Ulmus sp. TPQ last extant");

Date("=Garoga Waney Edge");

};

Boundary("End H9");

};

R_Date("ISGS-A0522 R H5 Pit",425,40)

{

Outlier("Charcoal",1);

};

R_Date("UCIAMS190540 M H4 Pit",345,20)

{

Outlier("General",0.05);

};

R_Date("UCIAMS190539 M H2 Pit",320,20)

{

Outlier("General",0.05);

};

R_Date("UCIAMS218479 M H12 Pit",315,15)

{

Outlier("General",0.05);

};

R_Combine("F184 Pit btw H1&Stockade")

{

Outlier("General",0.05);

R_Date("UCIAMS190538 M F184 Pit",305,20)

{

Outlier("SSimple",0.05);

};

R_Date("UCIAMS218477 M F184 Pit",330,20)

{

Outlier("SSimple",0.05);

};

};

Date("Date Garoga");

Interval("Interval Garoga",LnN(ln(20),ln(2)));

};

Boundary("End Garoga");

};

Difference("Duration H9","End H9","Start H9");

};

***Fig. 8. Briggs’s Run***

Options()

{

Resolution=1;

kIterations=3000;

};

Plot()

{

Outlier_Model("General",T(5),U(0,4),"t");

Outlier_Model("SSimple",N(0,2),0,"s");

D_Sequence("Fagus 25 rings to Waney Edge")

{

R_Date("UCIAMS226644 RY1002",345,20)

{

Outlier ("SSimple",0.05);

};

Gap(1.5);

R_Date("UCIAMS239718 RY1003-1004",385,15)

{

Outlier ("SSimple",0.05);

};

Gap(18.5);

R_Date("UCIAMS239719 RY1021-1023",320,15)

{

Outlier ("SSimple",0.05);

};

Gap(2);

R_Date("UCIAMS226645 RY1023-1025",305,20)

{

Outlier ("SSimple",0.05);

};

Gap(1);

Date("Fagus RY1025");

};

Sequence()

{

Boundary("Start Brigg's Run",U(1565,1635))

{

color="blue";

};

Phase("Brigg's Run")

{

R_Date("ISGSA0328 M",401,38)

{

Outlier("General",0.05);

};

R_Date("AA-7693 M",315,40)

{

Outlier("General",0.05);

};

R_Date("AA-AA-7417 M",290,37)

{

Outlier("General",0.05);

};

R_Date("UCIAMS226643 M",315,20)

{

Outlier("General",0.05);

};

Date("=Fagus RY1025");

Interval("Interval Brigg's Run",LnN(ln(20),ln(2)));

Date("Date Briggs Run")

{

color="orange";

};

};

Boundary("End Brigg's Run",U(1565,1635))

{

color="blue";

};

};

};

**Fig. 9. The alternative version of Klock Model 1b placing the samples from hearth contexts as representing latest use of House 1.**

Options()

{

Resolution=1;

kIterations=3000;

};

Plot("Klock")

{

Outlier_Model("General",T(5),U(0,4),"t");

Outlier_Model("Charcoal",Exp(1,-10,0),U(0,3),"t");

Outlier_Model("SSimple",N(0,2),0,"s");

//50% add to SDs in D_Sequence – see Methods

D_Sequence("Klock 45171.A1 Ulmus sp. F84 in H1")

{

Outlier ("SSimple",0.05);

R_Combine("RY1001-1002")

{

R_Date("RY1001-1002 UCIAMS226653",290,30)

{

Outlier ("SSimple",0.05);

};

R_Date("RY1001-1002 UCIAMS239720",300,23)

{

Outlier ("SSimple",0.05);

};

};

Gap(12.5);

R_Date("RY1010-1018 UCIAMS239721",360,23)

{

Outlier ("SSimple",0.05);

};

Gap(9);

R_Date("RY1021-1025 UCIAMS226654",355,30)

{

Outlier ("SSimple",0.05);

};

Gap(2);

Date("Ulmus sp. Bark Last Use F84");

};

Sequence()

{

Boundary("Start Klock");

Phase("Klock")

{

Sequence("House 1")

{

Boundary("Start H1");

Phase ("H1 Earlier but not Last Use")

{

Sequence("F84 earlier")

{

Boundary("Start F84 Pit");

Phase("Pit Lining equals Construction")

{

R_Date("UCIAMS-239714 Monocot F84 lining",360,15)

{

Outlier("SSimple",0.05);

};

};

Boundary("Making pit to use of F84");

Phase("Initial Use of F84")

{

R_Combine("Feature 84 Pit base H1 - assume one event")

{

Outlier("General",0.05);

R_Date("UCIAMS218474 M F84",365,15)

{

Outlier("SSimple",0.05);

};

R_Date("UCIAMS-239712 M F84",370,15)

{

Outlier("SSimple",0.05);

};

R_Date("UCIAMS-239713 M F84",350,15)

{

Outlier("SSimple",0.05);

};

};

};

Boundary("End Early Use F84");

};

Sequence()

{

Boundary();

Phase("F116 Pit House 1")

{

R_Date("UCIAMS218476 M F116 Pit H1",365,15)

{

Outlier("General",0.05);

};

};

Boundary();

};

Sequence()

{

Boundary();

Phase("F50 Pit House 1")

{

R_Date("ISGS-A0326 M F50 H1", 317, 38)

{

Outlier("General",0.05);

};

};

Boundary();

};

};

Boundary("Transition Earlier Use to Last Use");

Phase("Last Use H1")

{

R_Date("UCIAMS190562 B Hearth H1",355,20)

{

Outlier("General",0.05);

};

R_Date("UCIAMS190560 M F65 Hearth H1",325,15)

{

Outlier("General",0.05);

};

};

Before("Bark Last Use F84")

{

Date("=Ulmus sp. Bark Last Use F84");

};

Boundary("End House 1");

};

Sequence()

{

Boundary();

Phase("Other Klock Dates")

{

R_Date("ISGS-A0523 R F117 H8 Pit",480,40)

{

Outlier("Charcoal",1);

};

R_Date("UCIAMS190559 B H86 Pit",360,15)

{

Outlier("General",0.05);

};

R_Date("UCIAMS190561 B F36 H4 Pit",335,15)

{

Outlier("General",0.05);

};

R_Date("UCIAMS218473 B F20 Pit",325,15)

{

Outlier("General",0.05);

};

R_Date("AA-6418 M Hearth F106 H7", 315, 60)

{

Outlier("General",0.05);

};

Phase("F135 btw H3&5")

{

R_Date("UCIAMS218475 M F135 Pit",335,20)

{

Outlier("General",0.05);

};

D_Sequence("Klock Fraxinus sp. F135 btw H3&5 13 rings bark 2nd list")

{

R_Date("UCIAMS239711 RY1009-1013",330,20);

Gap(2);

Date("F135 bark");

};

};

};

Boundary();

};

Date("Date Klock Overall");

Interval("Interval Klock Overall",LnN(ln(20),ln(2)));

};

Boundary("End Klock");

};

};
