# Supplementary material for: Resolving Indigenous village occupations and social history across the long century of European permanent settlement in Northeastern North America: The Mohawk River Valley ~1450-1635 CE
Source: PLoS One. 2021 Oct 15;16(10):e0258555. doi: 10.1371/journal.pone.0258555 (PMC8519479; doi:10.1371/journal.pone.0258555)
Supplement: S3 File — This model employs the site models (Model 1) for Smith-Pagerie, Klock (Model 1b), Garoga and Brigg’s Run from those listed in S2 File (and as used for Figs 5–8). The data and models for the other eight sites are taken from the Model 2 versions in [65] with the change that the Interval statement now includes the LnN(ln(20),ln(2)) constraint (and we now employ IntCal20 [111]). Otherwise, the data and models for each site are the same as previously published (thus these sites have a uniform probability constraint placed on the start and end Boundaries between 1150 and 1635 CE as explained in [65]. Note: Zea mays (maize) samples are identified as M, Odocoileus virginianus (white-tailed deer) samples are identified as B, and organic residue samples are identified as R. For details, see S1 Table and [65]. Note, for reasons of display space, samples of Fagus grandifolia (beech) are listed just as “Fagus”. (DOCX) [file pone.0258555.s005.docx]

**S3 File. OxCal runfile for the model in Fig. 10.** This model employs the site models (Model 1) for Smith-Pagerie, Klock (Model 1b), Garoga and Brigg’s Run from those listed in File S2 (and as used for Figs. 5-8). The data and models for the other eight sites are taken from the Model 2 versions in [65] with the change that the Interval statement now includes the LnN(ln(20),ln(2)) constraint (and we now employ IntCal20 [111]). Otherwise, the data and models for each site are the same as previously published (thus these sites have a uniform probability constraint placed on the start and end Boundaries between 1150 and 1635 CE as explained in [65]. Note: *Zea mays* (maize) samples are identified as M, *Odocoileus virginianus* (white-tailed deer) samples are identified as B, and organic residue samples are identified as R. For details, see Table S1 and [65]. Note, for reasons of display space, samples of *Fagus grandifolia* (beech) are listed just as “Fagus”.

Options()

{

Resolution=1;

kIterations=3000;

};

Plot()

{

Outlier_Model("General",T(5),U(0,4),"t");

Outlier_Model("Charcoal",Exp(1,-10,0),U(0,3),"t");

Outlier_Model("SSimple",N(0,2),0,"s");

Sequence()

{

Boundary("Start Snell Pits",Date(U(1150,1635)));

Phase("Snell Pits")

{

R_Date("M-28 charred wood",1670,300)

{

Outlier("Charcoal",1);

};

R_Date("M-178 charred wood",1170,200)

{

Outlier("Charcoal",1);

};

R_Date("M-492 charred wood",794,200)

{

Outlier("Charcoal",1);

};

R_Date("UCIAMS190544 B",710,15)

{

Outlier("General",0.05);

};

R_Date("UCIAMS190542 B",710,20)

{

Outlier("General",0.05);

};

R_Date("UCIAMS190543 B",705,20)

{

Outlier("General",0.05);

};

R_Date("ISGS-A0327 M",691,39)

{

Outlier("General",0.05);

};

R_Date("UCIAMS192977 B",685,15)

{

Outlier("General",0.05);

};

Interval("Interval Snell",LnN(ln(20),ln(2)));

Date("Date Snell Pits");

};

Boundary("End Snell Pits",Date(U(1150,1635)));

};

Sequence()

{

Boundary("Start Pethick",Date(U(1150,1635)));

Phase("Pethick hearths or small pits")

{

R_Date("Beta 199857 C",730,70)

{

Outlier("Charcoal",1);

};

R_Date("Beta 198540 C",670,90)

{

Outlier("Charcoal",1);

};

R_Date("Beta 211490 C",560,40)

{

Outlier("Charcoal",1);

};

R_Date("Beta 22779 C",560,40)

{

Outlier("Charcoal",1);

};

R_Date("UCIAMS218494 M",675,15)

{

Outlier("General",0.05);

};

R_Date("UCIAMS218495 M",670,15)

{

Outlier("General",0.05);

};

R_Date("UCIAMS218496 M",595,20)

{

Outlier("General",0.05);

};

Interval("Interval Pethick",LnN(ln(20),ln(2)));

Date("Date Pethick");

};

Boundary("End Pethick",Date(U(1150,1635)));

};

Sequence()

{

Boundary("Start Second Woods",Date(U(1150,1635)));

Phase("Second Woods")

{

R_Combine("Feature 2 Shallow Pit Deposit - Assume One Event",8)

{

Outlier("General",0.05);

R_Date("UCIAMS190546 B Feature 2",405,15)

{

Outlier("Simple",0.05);

};

R_Date("UCIAMS190547 B Feature 2",375,15)

{

Outlier("Simple",0.05);

};

};

R_Date("UCIAMS190536 M",420,15)

{

Outlier("General",0.05);

};

R_Date("UCIAMS190535 M",380,20)

{

Outlier("General",0.05);

};

Interval("Interval Second Woods",LnN(ln(20),ln(2)));

Date("Date Second Woods");

};

Boundary("End Second Woods",Date(U(1150,1635)));

};

Sequence()

{

Boundary("Start Elwood",Date(U(1150,1635)));

Phase ("Elwood")

{

R_Date("UCIAMS190554 B Pit",420,20)

{

Outlier("General",0.05);

};

R_Date("AA-7410 M midden",409,49)

{

Outlier("General",0.05);

};

R_Date("AA-6425 M midden",380,50)

{

Outlier("General",0.05);

};

R_Date("UCIAMS190552 B Hearth", 380, 15)

{

Outlier("General",0.05);

};

R_Date("UCIAMS190553 B Hearth", 370, 20)

{

Outlier("General",0.05);

};

R_Date("AA-7697 M midden",288,49)

{

Outlier("General",0.05);

};

Interval("Interval Elwood",LnN(ln(20),ln(2)));

Date("Date Elwood");

};

Boundary("End Elwood",Date(U(1150,1635)));

};

Sequence()

{

Boundary ("Start Getman",Date(U(1150,1635)));

Phase("Getman")

{

R_Date("M-783 Charcoal context not stated TPQ",560,150)

{

Outlier("Charcoal",1);

};

R_Date("UCIAMS190557 House 3 Hearth B",450,20)

{

Outlier("General",0.05);

};

R_Combine("Feature 28 Pit Event",8)

{

Outlier("General",0.05);

R_Date("UCIAMS190558 House 3 B Pit",400,15)

{

Outlier("Simple",0.05);

};

R_Date("UCIAMS218481 House 3 M Pit",395,15)

{

Outlier("Simple",0.05);

};

};

R_Date("UCIAMS218482 General Midden M",405,15)

{

Outlier("General",0.05);

};

R_Date("UCIAMS218480 House 5 Pit M",400,15)

{

Outlier("General",0.05);

};

//R_Date("UCIAMS192976 House 1 Pit B",485,15)

//{

// Outlier("General",0.05);

//};

//cut as only larger outlier (>10%), at ca. 20% probablity

R_Date("UCIAMS192975 Pit B",355,15)

{

Outlier("General",0.05);

};

R_Date("UCIAMS190555 Pit B",345,20)

{

Outlier("General",0.05);

};

R_Date("UCIAMS190556 Pit B",330,20)

{

Outlier("General",0.05);

};

Interval("Interval Getman",LnN(ln(20),ln(2)));

Date("Date Getman");

};

Boundary("End Getman",Date(U(1150,1635)));

};

Sequence()

{

Boundary();

Phase("S-P")

{

Sequence()

{

Boundary("Start S-P");

//Phase("F60 pit: initial/only use - assumed start/early in site occupation")

//{

// R_Date("UCIAMS218493 M F60 Longhouse 5 Pit",480,15)

// {

// Outlier("General",0.05);

// };

//Small shallow pit 36" diameter and only 24" deep Funk & Kuhn (2003:61). Thus assume likely just ONE real use episode. Assume the Maize thus gives a date for the start of the pit and assume - based on 14C - that this is early in (even start of) site/house history. But, even so, as ca. start of the site, ca. 98% outlier. 100% outlier if included in next Phase. Thus exclude. Not known why a few decades too old.

// };

Phase("Majority of pits at site, some earlier, some later - thus range")

{

R_Date("ISGS-A0528 R F15 H1",445,40)

{

Outlier("Charcoal",1);

};

R_Date("UCIAMS190566 B F25 H1",375,15)

{

Outlier("General",0.05);

};

R_Date("UCIAMS190565 B F11 H1",370,15)

{

Outlier("General",0.05);

};

R_Date("UCIAMS190563 B F40 H1",360,20)

{

Outlier("General",0.05);

};

//R_Date("UCIAMS218491 Material?? F9 pit H1",265,15)

//{

// Outlier("General",0.05);

//};

//Exclude. 1. We do not know what material this sample comprised, so suspect. 2. the d13C value is rather different from all the others (site and whole Mohawk set) at -29.1. 3. If it is included in model as is it is a 100% outlier. Far too recent for rest of site. Hence exclude.

Sequence("F54 samples from Dark Lens")

{

//Older to current maize samples and then firing date for the end of the episode from the bark/waney edge dates. End of main occupation use of F54. Later pit (see section drawing) is subsequent.

Boundary();

Phase("F54 Dark Lens residual to current maize samples")

{

R_Date("AA-7405 M F54 H1",430,50)

{

Outlier("General",0.05);

};

R_Date("AA-6419 M F54 H1",405,50)

{

Outlier("General",0.05);

};

R_Date("UCIAMS218490 M F54 H1",325,30)

{

Outlier("General",0.05);

};

};

Boundary("Dark Lens Firing");

Before("Before Date of firing, from bark/waney wedge, of Dark Lens in F54")

{

D_Sequence("S-P 4457.1 Fagus 1 F54 G2 H1 28 rings")

{

R_Date("RY1001-1005",315,20)

{

Outlier("SSimple",0.05);

};

Gap(10);

R_Date("RY1011-1015",340,20)

{

Outlier("SSimple",0.05);

};

Gap(12.5);

R_Date("RY1023-1028",330,20)

{

Outlier("SSimple",0.05);

};

Gap(2.5);

Date("S-P Fagus Waney Edge");

};

D_Sequence("S-P 4457.1 Betula F54 G2 H1 20 rings")

{

R_Date("RY1001-1003 S-P",320,20)

{

Outlier("SSimple",0.05);

};

Gap(18);

R_Date("RY1020 Betula Bark",340,15)

{

Outlier("SSimple",0.05);

};

};

D_Sequence("S-P Fagus 2 F54 G2 H1 27 rings")

{

R_Date("UCIAMS239715 RY1001-1005",295,15)

{

Outlier("SSimple",0.05);

};

Gap(24);

Date("S-P Fagus Waney Edge");

};

};

};

};

Boundary("Pits to Hearth equals late/last occupation at site");

Phase("House 2 Hearth")

{

R_Date("UCIAMS218492 M F80 Longhouse 2 Hearth",350,15)

{

Outlier("General",0.05);

};

};

};

Date("Date Smith-Pagerie");

Interval("Interval Smith-Pagerie",LnN(ln(20),ln(2)));

};

Boundary("End S-P");

Boundary("Site use to post-site use");

Phase("Unburnt bone - post site occupation")

{

R_Date("UCIAMS190564 B unburnt F127 Longhouse 4 Hearth",345,20)

{

Outlier("General",0.05);

};

};

Boundary();

};

D_Sequence("Klock 45171.A1 Ulmus sp. F84 in H1")

{

Outlier ("SSimple",0.05);

R_Combine("RY1001-1002")

{

R_Date("RY1001-1002 UCIAMS226653",290,30)

{

Outlier ("SSimple",0.05);

};

R_Date("RY1001-1002 UCIAMS239720",300,23)

{

Outlier ("SSimple",0.05);

};

};

Gap(12.5);

R_Date("RY1010-1018 UCIAMS239721",360,23)

{

Outlier ("SSimple",0.05);

};

Gap(9);

R_Date("RY1021-1025 UCIAMS226654",355,30)

{

Outlier ("SSimple",0.05);

};

Gap(2);

Date("Ulmus sp. Bark Last Use F84");

};

Sequence()

{

Boundary("Start Klock");

Phase("Klock")

{

Sequence("House 1")

{

Boundary("Start H1");

Phase ("H1")

{

Sequence("F84 earlier")

{

Boundary("Start F84 Pit");

Phase("Pit Lining equals Construction")

{

R_Date("UCIAMS-239714 Monocot F84 lining",360,15)

{

Outlier("SSimple",0.05);

};

};

Boundary("Making pit to use of F84");

Phase("Initial Use of F84")

{

R_Combine("Feature 84 Pit base H1 - assume one event")

{

Outlier("General",0.05);

R_Date("UCIAMS218474 M F84",365,15)

{

Outlier("SSimple",0.05);

};

R_Date("UCIAMS-239712 M F84",370,15)

{

Outlier("SSimple",0.05);

};

R_Date("UCIAMS-239713 M F84",350,15)

{

Outlier("SSimple",0.05);

};

};

};

Boundary("End Early Use F84");

};

Sequence()

{

Boundary();

Phase("F116 Pit from House 1")

{

R_Date("UCIAMS218476 M F116 Pit H1",365,15)

{

Outlier("General",0.05);

};

};

Boundary();

};

Sequence()

{

Boundary();

Phase("Use H1")

{

R_Date("UCIAMS190562 B Hearth H1",355,20)

{

Outlier("General",0.05);

};

R_Date("UCIAMS190560 M F65 Hearth H1",325,15)

{

Outlier("General",0.05);

};

R_Date("ISGS-A0326 M F50 H1", 317, 38)

{

Outlier("General",0.05);

};

};

Boundary();

};

Date("Date House 1");

Before("Bark Last Use F84")

{

Date("=Ulmus sp. Bark Last Use F84");

};

};

Boundary("End Use H1");

};

Sequence()

{

Boundary();

Phase("Other Klock Dates")

{

R_Date("ISGS-A0523 R F117 H8 Pit",480,40)

{

Outlier("Charcoal",1);

};

R_Date("UCIAMS190559 B H86 Pit",360,15)

{

Outlier("General",0.05);

};

R_Date("UCIAMS190561 B F36 H4 Pit",335,15)

{

Outlier("General",0.05);

};

R_Date("UCIAMS218473 B F20 Pit",325,15)

{

Outlier("General",0.05);

};

R_Date("AA-6418 M Hearth F106 H7", 315, 60)

{

Outlier("General",0.05);

};

Phase("F135 btw H3&5")

{

R_Date("UCIAMS218475 M F135 Pit",335,20)

{

Outlier("General",0.05);

};

D_Sequence("Klock Fraxinus sp. F135 btw H3&5 13 rings bark 2nd list")

{

R_Date("UCIAMS239711 RY1009-1013",330,20);

Gap(2);

Date("F135 bark");

};

};

};

Boundary();

};

Date("Date Klock Overall");

Interval("Interval Klock Overall",LnN(ln(20),ln(2)));

};

Boundary("End Klock");

};

D_Sequence("Garoga 42354.E.1 F36 H9 Ulmus sp. 53 rings no bark")

{

R_Date("RY1003-1004",345,20)

{

Outlier ("SSimple",0.05);

};

Gap(47.5);

R_Date("RY1049-1053",320,20)

{

Outlier ("SSimple",0.05);

};

Gap(2);

Date("Garoga Ulmus sp. TPQ last extant");

};

D_Sequence("Garoga 42354.E.1 F36 H9 Fagus 19 rings to Waney Edge")

{

R_Date("RY1001-1003 G",325,20)

{

Outlier ("SSimple",0.05);

};

Gap(15);

R_Date("RY1015-1019",320,20)

{

Outlier ("SSimple",0.05);

};

Gap(2);

Date("Garoga Waney Edge");

};

Sequence()

{

Boundary("Start Garoga");

Phase("Garoga")

{

Sequence("House 9")

{

Boundary("Start H9");

Phase("House 9")

{

R_Date("Y-1381 F11 H9 charred wood",620,100)

{

Outlier("Charcoal",1);

};

//R_Date("AA-8370 M F37 Pit H9",585,40)

//{

// Outlier("General",0.05);

//};

//100% outlier when included and individual A value ca.5.4. Exclude.

Phase("F2 Pit H9")

{

// R_Date("AA-7695 M F2 Pit H9",431,39)

// {

// Outlier("General",0.05);

// };

//Outlier ca. 10% and individual A value ca. 8.2. Main reason even excluding AA-8370 that Model overall A value <60. Exclude.

//Model with outlier models applied achieves similar result re date estimate for site when these two dates left in, just poor Amodel value.

R_Date("AA-7403 M F2 Pit H9",410,60)

{

Outlier("General",0.05);

};

R_Date("UCIAMS218478 M F2 Pit H9",345,15)

{

Outlier("General",0.05);

};

R_Date("UCIAMS190537 M F2 Pit H9",335,20)

{

Outlier("General",0.05);

};

R_Date("AA-6417 M F2 Pit H9",300,50)

{

Outlier ("General",0.05);

};

};

};

Sequence("F36 Late Lobe TAQ H9")

{

Date("=Garoga Ulmus sp. TPQ last extant");

Date("=Garoga Waney Edge");

};

Boundary("End H9");

};

R_Date("ISGS-A0522 R H5 Pit",425,40)

{

Outlier("Charcoal",1);

};

R_Date("UCIAMS190540 M H4 Pit",345,20)

{

Outlier("General",0.05);

};

R_Date("UCIAMS190539 M H2 Pit",320,20)

{

Outlier("General",0.05);

};

R_Date("UCIAMS218479 M H12 Pit",315,15)

{

Outlier("General",0.05);

};

R_Combine("F184 Pit btw H1&Stockade")

{

Outlier("General",0.05);

R_Date("UCIAMS190538 M F184 Pit",305,20)

{

Outlier("SSimple",0.05);

};

R_Date("UCIAMS218477 M F184 Pit",330,20)

{

Outlier("SSimple",0.05);

};

};

Date("Date Garoga");

Interval("Interval Garoga",LnN(ln(20),ln(2)));

};

Boundary("End Garoga");

};

Sequence()

{

Boundary("Start Otstungo",Date(U(1150,1635)));

Phase("Otstungo House 1 Midden and Hearths")

{

R_Date("UCIAMS190551 Hearth B",355,20)

{

Outlier("General",0.05);

};

R_Date("UCIAMS190549 Hearth B",335,20)

{

Outlier("General",0.05);

};

R_Date("UCIAMS190550 Hearth B",310,15)

{

Outlier("General",0.05);

};

R_Date("UCIAMS190548 Hearth B",310,15)

{

Outlier("General",0.05);

};

R_Date("AA-7400 M",415,50)

{

Outlier("General",0.05);

};

R_Date("AA-7402 M",410,50)

{

Outlier("General",0.05);

};

R_Date("AA-6423 M",400,55)

{

Outlier("General",0.05);

};

R_Date("UCIAMS218483 M",400,15)

{

Outlier("General",0.05);

};

R_Date("AA-7398 M",380,55)

{

Outlier("General",0.05);

};

R_Date("UCIAMS218489 M",380,20)

{

Outlier("General",0.05);

};

R_Date("AA-7401 M",365,50)

{

Outlier("General",0.05);

};

R_Date("AA-7399 M",345,55)

{

Outlier("General",0.05);

};

R_Date("UCIAMS218487 M",340,15)

{

Outlier("General",0.05);

};

Interval("Interval Otstungo",LnN(ln(20),ln(2)));

Date("Date Otstungo");

};

Boundary("End Otstungo",Date(U(1150,1635)));

};

Sequence()

{

Boundary("Start Cayadutta Midden",Date(U(1150,1635)));

Phase("Cayadutta Midden")

{

//R_Date("AA-7689 M", 557, 58)

//{

// Outlier("General",0.05);

//};

//too old outlier, excluded

R_Date("AA-7690 M", 415, 56)

{

Outlier("General",0.05);

};

R_Date("AA-7407 M", 367, 52)

{

Outlier("General",0.05);

};

R_Date("AA-6421 M", 300, 50)

{

Outlier("General",0.05);

};

R_Date("UCIAMS-205965 M", 355, 20)

{

Outlier("General",0.05);

};

R_Date("UCIAMS-205966 M", 340, 15)

{

Outlier("General",0.05);

};

R_Date("UCIAMS-205967 M", 315, 15)

{

Outlier("General",0.05);

};

R_Date("UCIAMS-205968 M", 375, 15)

{

Outlier("General",0.05);

};

Interval("Interval Cayadutta Midden",LnN(ln(20),ln(2)));

Date("Date Cayadutta Midden");

};

Boundary("End Cayadutta Midden",Date(U(1150,1635)));

};

Sequence()

{

Boundary("Start Wormuth",Date(U(1150,1635)));

Phase("Wormuth")

{

R_Date("AA-6416 M",385,50)

{

Outlier("General",0.05);

};

R_Date("UCIAMS192700 M Pit",360,15)

{

Outlier("General",0.05);

};

R_Date("AA-6065 M",340,25)

{

Outlier("General",0.05);

};

R_Date("DIC-1176 Lowest Level Midden Charcoal",560,50)

{

Outlier("Charcoal",1);

};

R_Date("DIC-1177 Higher Up Midden Charcoal",250,50)

{

Outlier("Charcoal",1);

};

R_Date("DIC-1178 Fill of Burial #2",410,55)

{

Outlier("Charcoal",1);

};

Interval("Interval Wormuth",LnN(ln(20),ln(2)));

Date("Date Wormuth");

};

Boundary("End Wormuth",Date(U(1150,1635)));

};

D_Sequence("Fagus 25 rings to Waney Edge")

{

R_Date("UCIAMS226644 RY1002",345,20)

{

Outlier ("SSimple",0.05);

};

Gap(1.5);

R_Date("UCIAMS239718 RY1003-1004",385,15)

{

Outlier ("SSimple",0.05);

};

Gap(18.5);

R_Date("UCIAMS239719 RY1021-1023",320,15)

{

Outlier ("SSimple",0.05);

};

Gap(2);

R_Date("UCIAMS226645 RY1023-1025",305,20)

{

Outlier ("SSimple",0.05);

};

Gap(1);

Date("Fagus RY1025");

};

Sequence()

{

Boundary("Start Brigg's Run",U(1565,1635))

{

color="blue";

};

Phase("Brigg's Run")

{

R_Date("ISGSA0328 M",401,38)

{

Outlier("General",0.05);

};

R_Date("AA-7693 M",315,40)

{

Outlier("General",0.05);

};

R_Date("AA-AA-7417 M",290,37)

{

Outlier("General",0.05);

};

R_Date("UCIAMS226643 Phaseolus vulgaris",315,20)

{

Outlier("General",0.05);

};

Date("=Fagus RY1025");

Interval("Interval Brigg's Run",LnN(ln(20),ln(2)));

Date("Date Briggs Run")

{

color="orange";

};

};

Boundary("End Brigg's Run",U(1565,1635))

{

color="blue";

};

};

};
